# Determinants of short interpregnancy intervals in high-income countries: a systematic review

**DOI:** 10.1080/26410397.2025.2545699

**Published:** 2025-09-01

**Authors:** J. Dunne, D. Foo, J. Jancey, G. Pereira, B. Kefale, D. G. Belay, G. Dhamrait, A. T. Gebremedhin, K. B. Mruts, S. D. Nyadanu, A. Roy, G. A. Tessema

**Affiliations:** a Research Fellow, Curtin School of Population Health, Faculty of Health Sciences, Curtin University, Bentley, Australia; Research Fellow, enAble Institute, Curtin University, Bentley, Australia.; b Post-doctoral Research Associate, Yale School of the Environment, Yale University, New Haven, CT, USA; c Professor, Curtin School of Population Health, Faculty of Health Sciences, Curtin University, Bentley, Australia; d Professor, Curtin School of Population Health, Faculty of Health Sciences, Curtin University, Bentley, Australia; Professor, enAble Institute, Curtin University, Bentley, Australia; e Research Assistant, Curtin School of Population Health, Faculty of Health Sciences, Curtin University, Bentley, Australia; f Research Assistant, Curtin School of Population Health, Faculty of Health Sciences, Curtin University, Bentley, Australia; g Research Fellow, Queensland Brain Institute, University of Queensland, St Lucia, Australia; h Research Fellow, Curtin School of Population Health, Faculty of Health Sciences, Curtin University, Bentley, Australia; Research Fellow, School of Nursing and Midwifery, Edith Cowan University, Joondalup, Australia; i Research Assistant, Curtin School of Population Health, Faculty of Health Sciences, Curtin University, Bentley, Australia; j Research Fellow, Curtin School of Population Health, Faculty of Health Sciences, Curtin University, Bentley, Australia; k Research Fellow, School of Population Health, Faculty of Health Sciences, Curtin University, Bentley, Australia; l Associate Professor, Curtin School of Population Health, Faculty of Health Sciences, Curtin University, Bentley, Australia; Associate Professor, School of Public Health, University of Adelaide, Adelaide, Australia

**Keywords:** pregnancy spacing, interpregnancy intervals, birth spacing, contraception, pregnancy intention

## Abstract

Short interpregnancy intervals (IPIs) of <6–18 months are associated with adverse maternal and child outcomes. This study aimed to identify the individual, relationship, community, and societal factors that influence short IPIs in high-income countries. A comprehensive search was undertaken in CINAHL Plus, Ovid/EMBASE, Ovid/MEDLINE, Ovid/PsycINFO, ProQuest, PubMed, Scopus, Web of Science, and Google Scholar for articles published in English from 1st January 1990 to 26th October 2023. Studies were included if they reported an effect estimate of at least one determinant of pregnancy spacing in a high-income country. The quality of the included studies was assessed using the Johanna Briggs Institute Critical Appraisal Tool and Cochrane Risk Assessment Tool. Multi-level factors at the individual, relationship, community, and societal level were systematically identified through the socio-ecological model. This study is registered with PROSPERO (CRD42020176311). Of 2005 unique articles, 220 were identified for full-text review, and 55 met the inclusion criteria representing a total of 27,103,055 women from 13 high-income countries. All the included studies were deemed to be of moderate to high quality. Most of the studies reported determinants of short IPI at the individual level, with non-use of contraception the most common reported factor. Peer influence was a factor at the relationship level, and access to health care and reproductive services were impactful at the community and societal levels, respectively. Future research and efforts should support the development and implementation of policies and practices that support optimum pregnancy spacing from a comprehensive socio-ecological position.

## Plain language summary

Too short a space between births can lead to poor health outcomes for mothers and children. Factors that influence when and why women and families choose to space their children largely remain unknown, especially in high-income countries. This study aimed to identify and understand the factors that influence birth spacing. Rather than focus solely on women's individual actions, we took a socio-ecological approach. This meant looking at broader factors that influence family planning. These could include the influence of relationships; the availability of services within communities; and how government policy and legislation affect reproductive rights. The majority of the 55 studies included in this review focused on individual use of contraception on birth spacing. Other individual factors included the number of children women had, their intention to conceive, their educational level, and their age. At the relationship level, peer influence had a strong impact on birth spacing, particularly from male partners. Easy access to health care post pregnancy was influential in reducing short birth spacing at the community level. So were policies and legislation that support access to reproductive health services. The development of policies and practices that take a socio-ecological position can empower women and families to make decisions that can lead to better health outcomes for mothers and babies.

## Résumé

Un intervalle court de moins de 6 à 18 mois entre deux grossesses est associé à des conséquences néfastes pour la mère et l’enfant. Cette étude visait à identifier les facteurs individuels, relationnels, communautaires et sociétaux qui influencent un espacement court des grossesses dans des pays à revenu élevé. Une recherche exhaustive a été menée dans CINAHL Plus, Ovid/EMBASE, Ovid/MEDLINE, Ovid/PsycINFO, ProQuest, PubMed, Scopus, Web of Science et Google Scholar pour des articles publiés en anglais entre le 1^er^ janvier 1990 et le 26 octobre 2023. Les études ont été incluses si elles faisaient état d’une estimation de l’effet d’au moins un déterminant de l’espacement des grossesses dans un pays à revenu élevé. La qualité des études incluses a été évaluée avec l’outil d’évaluation critique du Johanna Briggs Institute et l’outil d’évaluation des risques de Cochrane. Les facteurs multiniveaux aux niveaux individuel, relationnel, communautaire et sociétal ont été identifiés de manière systématique grâce au modèle socioécologique. Cette étude est enregistrée dans PROSPERO (CRD42020176311). Sur 2005 articles uniques, 220 ont été sélectionnés pour un examen du texte intégral et 55 ont réuni les critères d’inclusion, représentant un total de 27 103 055 femmes issues de 13 pays à revenu élevé. Toutes les études incluses ont été considérées de qualité moyenne à élevée. La plupart des études ont fait état de déterminants d’un court intervalles entre les grossesses au niveau individuel, la non-utilisation d’une contraception étant le facteur le plus fréquemment cité. L’influence des pairs était un facteur au niveau relationnel, et l’accès aux soins de santé et aux services de santé reproductive a eu un impact aux niveaux communautaire et sociétal, respectivement. Les recherches et activités futures devraient soutenir l’élaboration et la mise en œuvre de politiques et pratiques favorisant un espacement optimal des grossesses dans une perspective socioécologique globale.

## Resumen

Los intervalos intergenésicos (IIG) cortos de <6 a 18 meses están asociados con resultados adversos para la salud materna e infantil. Este estudio buscaba identificar los factores individuales, de relación, comunitarios y sociales que influyen en los IIG cortos en países de altos ingresos. Se realizó una búsqueda exhaustiva en CINAHL Plus, Ovid/EMBASE, Ovid/MEDLINE, Ovid/PsycINFO, ProQuest, PubMed, Scopus, *Web of Science* y *Google Scholar* de artículos publicados en inglés entre el 1 de enero de 1990 y el 26 de octubre de 2023. Los estudios fueron incluidos si informaban una estimación del efecto de, como mínimo, un determinante de espaciamiento entre embarazos en un país de altos ingresos. Se evaluó la calidad de los estudios incluidos utilizando la Herramienta de Evaluación Crítica del Instituto Johanna Briggs y la Herramienta Cochrane de Evaluación de Riesgos. Por medio del modelo socioecológico se identificaron sistemáticamente factores multinivel a nivel individual, de relación, comunitario y social. Este estudio está registrado con PROSPERO (CRD42020176311). De 2005 artículos únicos, 220 fueron identificados para la revisión de texto completo y 55 reunían los criterios de inclusión, representando un total de 27,103,055 mujeres de 13 países de altos ingresos. Todos los estudios incluidos se consideraron de calidad moderada a alta. La mayoría de los estudios informaron determinantes de IIG cortos a nivel individual, y la ausencia de uso de anticonceptivos fue el factor informado con mayor frecuencia. La influencia de pares fue un factor a nivel de relación, y el acceso a servicios de salud y de salud reproductiva fue impactante a nivel comunitario y social, respectivamente. Futuros esfuerzos e investigaciones deberían apoyar la formulación y aplicación de políticas y prácticas de espaciamiento óptimo entre embarazos desde una perspectiva socioecológica integral.

## Introduction

Interpregnancy interval (IPI) is the time or spacing between the birth of a previous child and the conception of the next pregnancy. There is evidence that short IPIs (<6–18 months) are associated with increased risk of maternal health^1–3^ and adverse perinatal^4–8^ outcomes. The causal mechanism that underpins these associations remains unclear; however, a proposed hypothesis is that women conceiving shortly after birth do not get adequate time: (i) to replenish their nutritional reserve depleted from the prior pregnancy, (ii) to recover from infections that cause inflammatory disease such as endometritis,^9^ (iii) for uterine incisions to heal after caesarean section, and (iv) to lose excess weight gained during the prior pregnancy.^10,11^ Beyond immediate adverse maternal and perinatal health outcomes, short IPIs are also associated with long-term negative health outcomes including reduced child longevity,^12,13^ child behavioural and developmental outcomes,^14–16^ and familial disruptions.^17^ For example, a study in Finland found that couples with short IPI (<18 months) had up to 50% higher risk of divorce compared to couples whose children were born after an IPI of 48 months.^17^

In response to existing evidence, primarily conducted in low-and middle-income countries, the World Health Organization made a recommendation in 2005 that women should wait at least 24 months following a live birth for better maternal and child health outcomes.^18^ This recommendation aligns with the fundamental human right to decide freely and responsibly on the number and spacing of children, as recognised by the United Nations in the Convention on the Elimination of All Forms of Discrimination Against Women.^19^ While recent evidence argues that the WHO recommendation of a “24-month” waiting time could be longer, particularly in high-income countries,^18^ evidence continues to support the harmful role of short (<6–18 months) IPI on adverse perinatal and maternal outcomes.^20,21^ Considering the current evidence, in 2019 the American College of Obstetrics and Gynecology recommended women avoid attempting pregnancy within 6 months of giving birth and advised of the risks and benefits of conceiving again within 18 months. Similarly, Healthy People 2030 targets aim to achieve a 6% reduction in the number of pregnancies that occur less than 18 months after a previous birth in the United States (US).^22^ In the UK, the Faculty of Sexual & Reproductive Healthcare recommended women be advised of the risks of conceiving within <12 months of IPI after childbirth.^23^

IPI can be considered a modifiable health behaviour; yet the associated risk factors and determinants that influence IPIs are likely to be diverse. Ensuring women have access to information, education, and means to achieve their desired IPI is crucial for upholding reproductive rights and promoting health equity. Research to increase our understanding of the determinants of IPIs is critical to informing any health interventions to promote optimal IPIs for improved maternal, child and family outcomes. A recent systematic review focusing on low- and middle-income countries found that sociodemographic factors such as maternal education, socio-economic level, sex of the preceding child, and reproductive and health services factors such as previous pregnancy outcome, breastfeeding, and contraceptive use, were associated with short IPIs.^24^ Although factors influencing IPI could vary across regions, there is an absence of evidence synthesis on factors associated with short IPIs in high-income countries.

### Objectives

**Figure 1.**
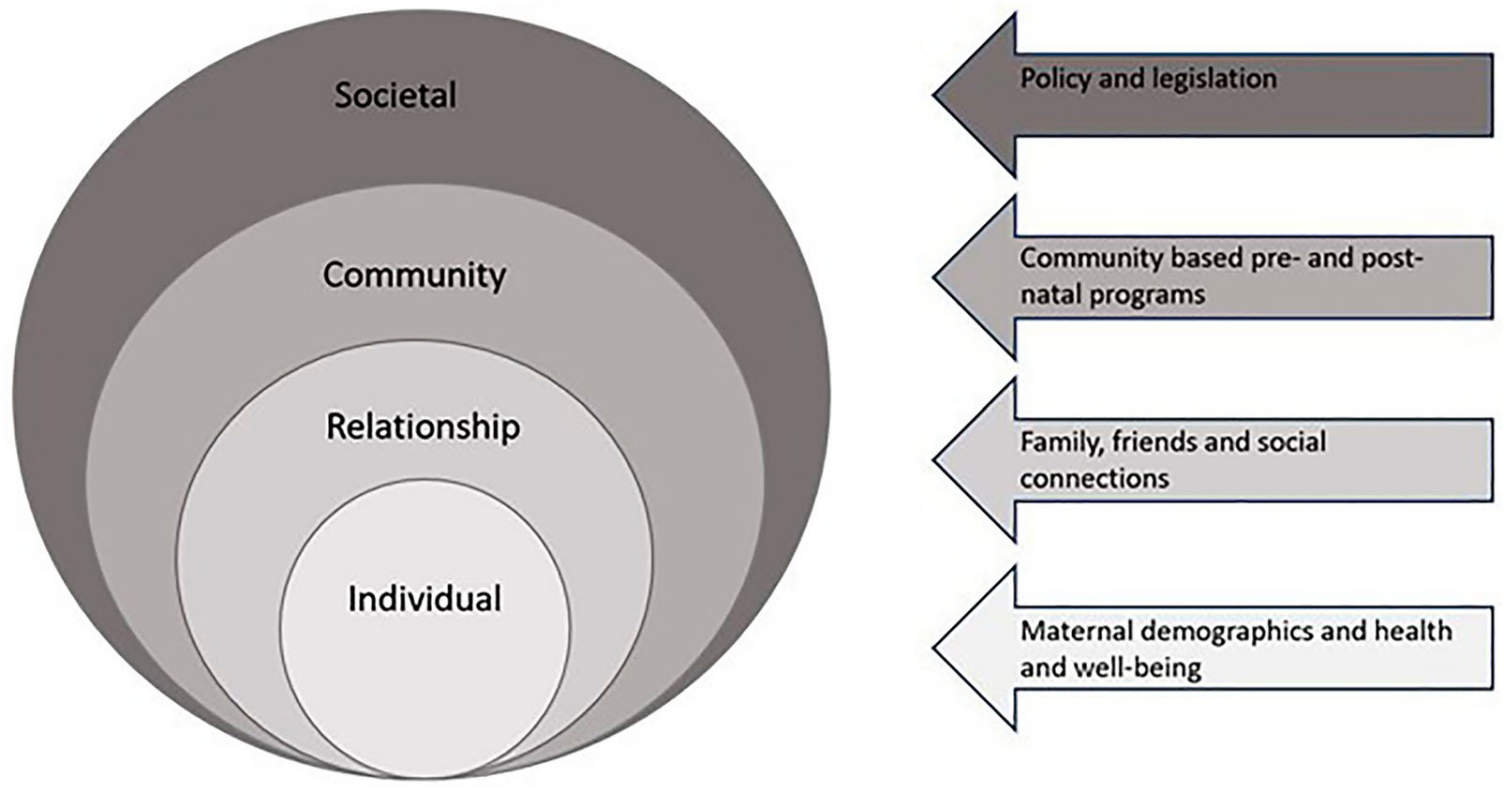
The socio-ecological model adapted from McLeroy et al^25^

The socio-ecological model acknowledges that there is a dynamic and complex interplay between individuals and their environment, acknowledging the level of factors that influence health behaviours^25^ (Figure 1). The model, originally developed by Bronfenbrenner's ecological systems theory,^26^ provides a framework to understand the multiple levels of influence on health behaviours. The levels of influence on short IPI may include individual level (personal and biological); relationship level factors (family and friends); community level factors (workplace, schools, and neighbourhoods); and societal level factors (policies and legislation). Consequently, this study aimed to comprehensively and systematically investigate the factors associated with short IPIs in high-income countries, applying a socio-ecological model^25^ to help inform the development of health interventions to reduce adverse outcomes associated with short IPIs.

## Methods

This systematic review followed the recommendations by the Preferred Reporting for Systematic Review and Meta-Analysis (PRISMA) reporting guidelines.^27^ The review protocol was prospectively registered in the International Prospective Registration of Systematic Reviews (PROSPERO) and is publicly available (Registration # CRD42020176311).

### Eligibility criteria, information sources, and search strategy

We conducted a comprehensive systematic search for peer-reviewed literature using eight electronic databases: CINAHL Plus, Ovid/EMBASE, Ovid/MEDLINE, Ovid/PsycINFO, ProQuest, PubMed, Scopus, and Web of Science, using a combination of medical subject headings, keywords, and search terms (see Supplementary Table 1). The initial databases were searched from 1st January 1990 until 19th July 2022, with an updated search conducted using the same search criteria on the 26th October 2023. Additionally, we searched Google Scholar for grey literature and undertook backward citation chaining of the reference list of the included studies for potentially relevant records that were not identified by the electronic search.

### Study selection criteria

Studies were eligible for inclusion if they satisfied four inclusion criteria: (i) study design criterion: observational (e.g. cross-sectional, case-control, nested case-control, case-cohort, and cohort studies) and experimental/intervention studies; (ii) effect estimate criterion: studies reporting measures of association (e.g. odds ratio, relative risk, risk difference, hazard ratio, beta-coefficient) with 95% confidence intervals (CI); (iii) country income group criterion: studies conducted in high-income countries according to the World Bank classification^28^; and (iv) outcome criterion: studies examining time between pregnancies or births as the outcome variable. For the outcome criterion, all included studies required women to have had at least two pregnancies to calculate IPIs. Studies were excluded if they were: published in languages other than English, not conducted in humans, and were reviews, editorials, commentaries, letters, case studies, or case series.

All unique records identified from the electronic database were imported into an Endnote library. In the first stage of the review, two reviewers (DF and JD) screened and reviewed the titles and abstracts of all identified records retrieved during the searches. Four reviewers performed the full-text review of the identified articles (DF, JD, KBM, BK). All reviewers made decisions regarding inclusion/exclusion and the primary reason for exclusion was documented. An additional reviewer (GAT) resolved any disagreements between the reviewers during each of the screening and review stages.

### Data extraction

For each included study, data were extracted by at least two reviewers (DF, JD, KBM, GD, BK) using a standardised data collection form adapted from Joanna Briggs Institute (JBI) Qualitative Data Extraction Tool,^29^ which included: author and year published, study aim, study design, sample size, geographic location, participant demographics, definition and ascertainment of exposure, outcome and adjustment variables, effect sizes and confidence intervals, and author acknowledged limitations.

### Assessment of risk of bias

Risk of bias for the included studies was independently assessed by at least two reviewers (DF, JD, KBM, GD, BK). For observational studies, we used the JBI Critical Appraisal tool to assess the risk of bias.^29^ Using this tool, studies were assessed for the risk of bias of observational cross-sectional, case-control, nested case-control, and cohort studies where relevant. For cross-sectional studies, eight items were checked as above, with studies rated “high” (0–4), “moderate” (5–7), and “low” (7–8). For case-control and cohort studies, 10 items were checked as “yes” (1), “unclear” (0), and “no” (0). The “yes” items were summed to total scores, which were rated “high” (0–5), “moderate” (6–8), and “low” (9–10) risk of bias. For intervention studies, we used the Cochrane risk-of-bias tool to assess the risk of bias.^30^ The Cochrane risk-of-bias tool addressed five major domains: selection bias (random sequence generation and allocation concealment), performance bias (blinding of participants and personnel and other potential threats to validity), detection bias (blinding of outcome assessment and other potential threats to validity), attrition bias (incomplete outcome data), and reporting bias (selective outcome reporting).^30^ Consistent with the Cochrane risk-of-bias tool scoring, articles were classified as low risk of bias, some concerns of bias, or high risk of bias.^30^

### Data synthesis

We performed a narrative synthesis and tabular presentation of all included studies underpinned by the socio-ecological model as a conceptual framework. A qualitative table was presented to illustrate the individual, relationship, community, and societal factors that investigated the determinants of IPIs. The extracted data were analysed using an deductive approach as recommended by the socio-ecological model.^25^ The key characteristics of the included studies and methodology details were also tabulated and discussed (Table 1).

**Table 1.** Socio-ecological factors of short interpregnancy intervals reported in the included studies (*n* = 55)

Socio-ecological level	Sub-level	Number of studies	First author (year of study)
Individual level			
Maternal demographics	Maternal age	7	Al-Rumhi^37^, Al-Nahedh^58^, Brown^48^, Cheslack-Postova^59^, Kaharuza^60^, Mishra^52^, White^55^
	Age at first birth	4	Al-Rumhi^37^, French^61^, Gemmill^57^, Iacobelli^46^
	Ethnicity/race	5	Delara^32^, Gemmill^57^, Krans^62^, Lewis^63^, White^55^
	Place of birth	3	Cheslack-Postova^59^, French^61^, Olorunsaiye^64^
	Immigration pathway	1	Olorunsaiye^64^
	Educational level	9	Abdel-Fattah^49^, Bennett^65^, Cheslack-Postova^59^, Gemmill^57^, Holowko^51^, Kaharuza^60^, Mishra^52^, Raneri^53^, White^55^
	Employment status	2	Abdel-Fattah^49^, Mishra^52^
	Socio-economic status	5	Abdel-Fattah^49^, Cheslack-Postova^59^, Gold^66^, Holowko^51^, Kaharuza^60^
	Financial stress	2	Holowko^51^, Mishra^52^
	Residence/location	2	Brown^48^, Mishra^52^
	Type of housing	1	Kaharuza^60^
	Relationship status	7	Cheslack-Postova^59^, Gemmill^57^, Kaharuza^60^, Mishra^52^, Raneri^53^, Waynforth^54^, White^55^
Physical health	Physical activity	1	Mishra^52^
	BMI	1	Mishra^52^
	Self-rated health	1	Mishra^52^
	History of chronic disease	2	Abdel-Fattah^49^, Krans^62^
	Lead poisoning	1	Lane^38^
	Blood borne virus status	2	Krans^62^, Lindsay^39^
Mental health	Self-rated mental health	1	Mishra^52^
	Depressive symptoms	2	Backley^92^, Bennett^65^, Gunst^45^ (postpartum depression)
	Psychiatric disorder	1	Krans^62^
	Schizophrenia	1	Gupta^67^
	Sleep disturbance	1	Gunst^45^
Sexual health	Contraceptive use	14	Bennett^65^, Brunson^74^, de Bocanegra^32^, de Bocanegra^68^, El-Kamary^56^, Gifford^69^, Harney^70^, Isquick^71^, Krans^62^, Lewis^63^, Liberty^72^, Mishra^52^, Stevens-Simons^73^, White^55^
	Menstrual cycle	1	Kaharuza^60^
	Pregnancy intention	6	Cha^40^, El-Kamary^56^, Gemmill^57^, Abdel-Fattah^49^, Kaharuza^60^, Lewis^63^, Raneri^53^
	Resumption of sexual intercourse	2	Knutson^75^, Lewis^63^
Reproductive and obstetric history	Fertility problems	1	Mishra^52^
	Year of birth	1	Albrechtsen^50^
	Parity	8	Al-Nahedh^58^, Abdel-Fattah^49^, Brown^48^, Gemmill^57^, Kaharuza^60^, Krans^62^, Waynforth^54^, White^55^
	Plurality	1	Waynforth^54^
	Previous perinatal loss	2	Al-Rumhi^37^, Mishra^52^
	Previous gestational diabetes mellitus	1	Ali^76^
	Previous breech presentation	2	Albrechtsen^50^, Smith^77^
	Previous preterm birth	1	Albrechtsen^50^
	Previous mode of delivery	4	Gottvall^47^, Albrechtsen^50^, Cheslack-Postova^59^, Smith^77^
	Perineal tear	1	Woolner^93^
	Substance use during previous pregnancy	3	Kaharuza^60^, Krans^62^, Mishra^52^
**Relationship level**			
Paternal characteristics	Occupation	1	Abdel-Fattah^49^
	Beliefs regarding birth spacing	1	Abdel-Fattah^49^
Grandparent characteristics	Educational level	1	Holowko^51^
**Family characteristics**	Sex ratio of children	2	Abdel-Fattah^49^, Waynforth^54^
	Previous death of child	2	Albrechtsen^50^, Plana-Ripoll^35^
	History of IPV	2	Raneri^53^, Rozario^79^
Peer influence	Friends influence	2	Raneri^53^, Reese^78^
	Family influence	1	Reese^78^
	Religion influence	1	Reese^78^
**Community level**			
	Access to primary care	1	Brown^48^
	Family planning services	3	de Bocanegra^32^, El-Kamary^56^, Kan^42^
	Home-visiting program	4	El-Kamary^56^, Goyal^34^, Rubin^81^, Yun^36^
	Prenatal care program	1	Heaman^80^
	Phone-based counselling	1	Katz^43^
**Societal level**			
	Insurance coverage/Medicaid	6	Arora^82^, Gemmill^57^, Liu^83^, Patel^84^, Steenland^44^, White^55^
	Hospital religious denomination	1	Caldwell^31^
	Receiving social assistance	1	Brown^48^

## Results

### Study selection and study characteristics

**Figure 2.**
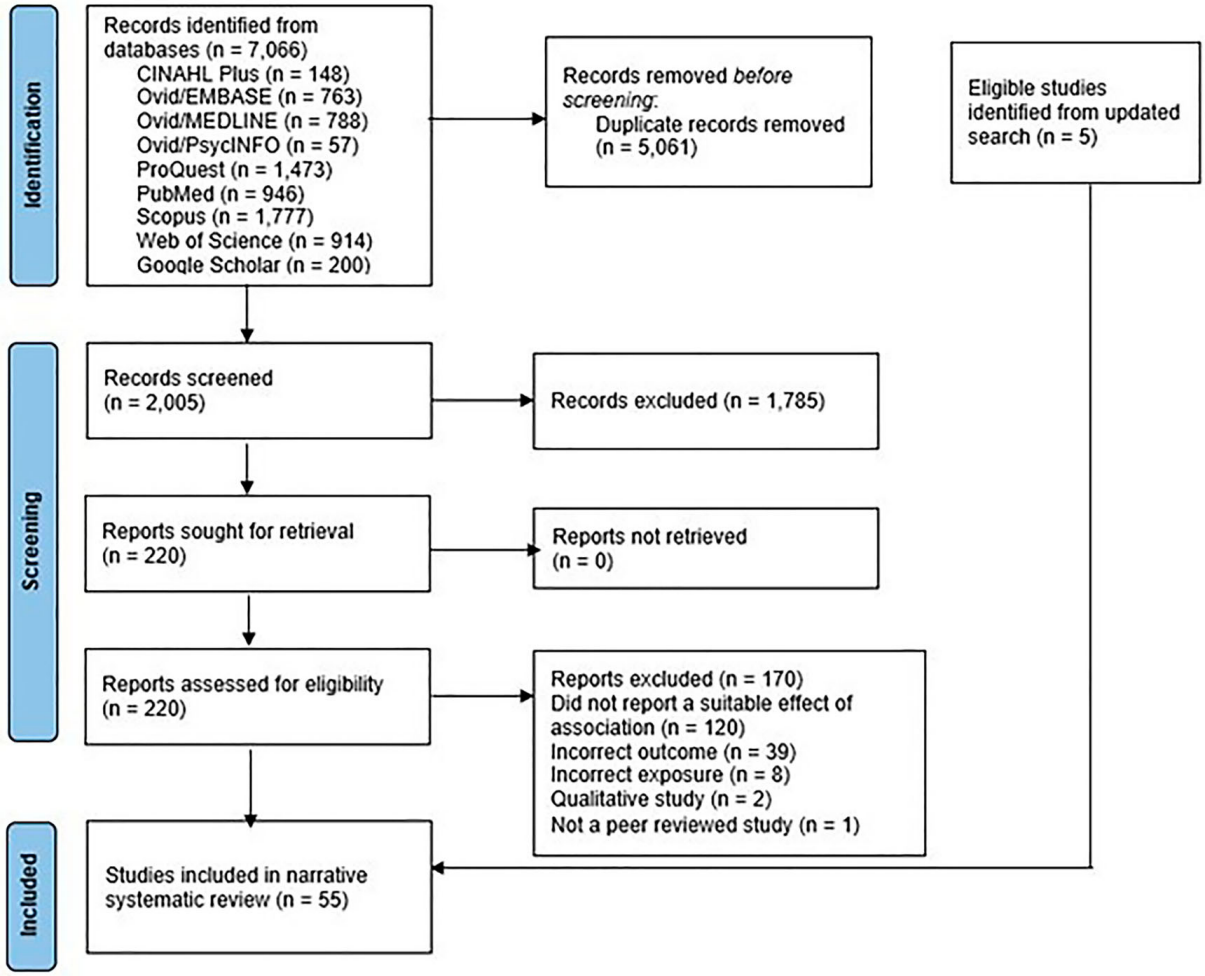
Systematic review of the literature examining the determinants of short interpregnancy intervals in high-income countries

A total of 55 articles met the inclusion criteria (Figure 2). The included studies were conducted between 1995 and 2023 with the majority conducted in the US (67%) (see Supplementary Table 2). Among these studies, 80% were cohort studies (*n* = 44), six were cross-sectional studies, three intervention studies, and two case-control studies. The participants in the included studies were parous women (*n* = 27,103,055) with sample sizes ranging from 249 to 14,873,995. With the exception of the three intervention studies, all participants completed a self-report questionnaire on the determinants of pregnancy spacing.

There was a diversity in the language used to describe measurements of the outcome (i.e. pregnancy spacing). IPI was the most common measure (*n* = 29) reported by the included studies. Within these studies, there were also variations in the definition of short IPIs, ranging from IPIs of ≤6 months^31–36^ to IPI <24 months.^31–37^ Thirteen studies reported birth-to-pregnancy (BTP) which is synonymous with IPI, of which three studies^38–40^ labelled IPI as a rapid repeat pregnancy (RRP). A further 13 studies used interbirth interval (IBI), defined as the time between successive live births,^41^ as an outcome measure with a short IBI ranging from an IBI of <12 months^42,43^ to an IBI of <48 months.^44^ The included studies used various comparative approaches. Most studies (76%, *n* = 42) compared different categories of IPI, with the primary comparison between short IPIs (using pre-defined thresholds of <6,<12, <18, or <24 months) and longer intervals. A smaller proportion (15%, *n* = 8) analysed IPI as a continuous variable, examining how various factors increased or decreased the interval length. The remaining studies (*n* = 5) employed survival analysis methods to analyse time-to-next pregnancy without predefining specific interval categories (see Supplementary Table 4). The majority of the studies reported a low risk of bias using the JBI critical appraisal tool,^29^ with three studies^39,45,46^ scoring as moderate risk. All three intervention studies^42,43,47^ were marked as low risk of bias using the Cochrane risk assessment.^30^

### Socio-ecological factors

Most of the included studies reported risk factors for short IPI at the individual level (65%). One study^48^ reported factors that influence pregnancy spacing across all four levels of the socio-ecological model, seven studies^49–55^ reported factors for the individual and relationship level, with three studies^32,56,57^ reporting factors in the individual and societal levels. In total there were 38 individual factors, covering domains related to maternal demographics (*n* = 12), physical health (*n* = 6), mental health (*n* = 5), sexual health (*n* = 4), and reproductive and obstetric history (*n* = 11). At the relationship level, there were nine factors, covering peer influence (*n* = 3), family characteristics (*n* = 3), paternal characteristics (*n* = 2), and grandparental characteristics (*n* = 1). At the community level, there were five factors representing access to health care, family planning, home-visiting program, prenatal care, and phone-based counselling services. At the societal level, there were three factors representing insurance coverage and Medicaid policy, receiving social assistance and hospital religious denomination.

#### Individual level

The majority of studies (*n* = 32) reported individual level factors associated with short IPI under six domains such as maternal demographics (*n* = 12), physical health (*n* = 6), mental health (*n* = 5), sexual health (*n* = 4), and reproductive and obstetric history (*n* = 11). Seven studies^37,48,52,55,58–60^ found that maternal age at birth was a determinant of pregnancy spacing, of which four studies^37,52,58,59^ reported advanced maternal age ≥ 35 years as a risk factor for shorter birth spacing, and three studies^37,48,55^ reported young maternal age (age <24 years) as a risk factor. Four studies^37,46,57,61^ found that being of a younger age at first birth was a risk for shorter birth spacing, with one study^46^ finding that mothers younger than <18 years at first singleton birth had almost three times the odds of having short (<18 months) IBI compared to mothers aged 18–29 years. Five studies^33,55,57,62,63^ described the influence of ethnicity on birth spacing, with one cohort study^33^ reporting that US women of Asian and Pacific Islander background had higher rates of IPI <6 months compared to Caucasian women (aOR 3.31 95% CI 2.70–4.10). Three studies^59,61,64^ found that place of birth had a strong influence on pregnancy spacing with varying effects dependent on the place of birth and country of residence. One study^64^ considered the immigration pathway as a determinant of IBI, finding that women who immigrated to the USA for education or employment purposes were at reduced risk of an IBI ≥36 months, compared to US-born residents (aOR 4.57 95% CI 1.57–9.58).

Nine studies^49,51–53,55,57,59,60,65^ investigated maternal educational status as a determinant for pregnancy spacing, all of which reported that increased educational attainment was associated with reduced risk of shorter pregnancy spacing. Two studies^49,52^ found that women engaged in employment have longer IBIs compared to women who were not employed. The influence of socio-economic status on pregnancy spacing varied across regions, with a Saudi Arabian^49^ study finding lower socio-economic status to be a protective factor for shorter IBI, an Australian study^51^ finding no effect, and two US studies^59,66^ reporting it as a risk factor for shorter IPIs. Women subjected to financial stress were less likely to experience shorter pregnancy spacing in two Australian studies.^51,52^ Residing in an urban^48^ or rural^52^ location had a marginal impact on pregnancy spacing; however, a Danish study^60^ found that type of housing was significant, with women residing in a flat having increased risk of a short IPI <9 months (aOR 1.72 95% CI 1.13–2.64) compared to women who resided in a bungalow. Of the seven studies^52–55,57,59,60^ that considered relationship status under maternal demographics, four^54,55,59,60^ reported no association between relationship status and pregnancy spacing.

Physical health factors were investigated under six sub-domains (physical activity; body mass index (BMI); self-rated health; history of chronic disease; lead poisoning; HIV status). An Australia-based study^52^ found that women who reported low physical activity were at increased risk of shorter IBI; however, women that were underweight or reported higher self-rated health scores had higher IBI. Two studies^39,62^ reported conflicting results on the influence of a chronic disease on birth spacing; one^39^ found that chronic disease was protective to short IBI (aHR 0.67 95% CI 0.41–0.68) while another study^62^ reported chronic hypertension a risk factor for IPI <18 months (OR 1.63 95% CI 1.08–2.46). There was no evidence of association between diabetes mellitus,^62^ HIV status,^39,62^ or Hepatitis C^62^ with pregnancy spacing. A US study^38^ found that lead poisoning during childhood was a significant factor in increasing the odds of an RRP (aOR 1.59 95% 1.04–2.43).

Mental health was examined under five sub-domains (self-rated mental health; depressive symptoms; psychiatric disorders; schizophrenia; sleep disturbance). No association was found between depressive symptoms,^45,65^ psychiatric disorders,^62^ and self-rated mental health^52^ with pregnancy spacing. However, in one study, a diagnosis of schizophrenia was found to be a risk factor for BTP of <12 months (aOR 1.31 95% CI 1.07–1.59).^67^

Sexual health was examined under four sub-domains (contraceptive use, menstrual cycle, pregnancy intention, and resumption of sexual intercourse), with contraceptive use being the most commonly studies factor (14 studies). Of the 14 studies^32,52,55,56,62,63,65,68–74^ that investigated the association between contraception use and pregnancy spacing, all indicated that the use of contraception was a protective factor for birth spacing. Five studies^55,62,68,71,74^ specifically examined the type of contraception, finding that the use of long-acting reversible contraceptive (LARC) methods was most effective in preventing short interpregnancy spacing. One study^60^ reported that an irregular menstrual cycle (aOR 1.69 95% CI 1.13–2.54) was associated with a risk of short IPI <9 months. The early resumption of sexual intercourse after the previous birth was also associated with shorter BTP.^63,75^ Of the six studies^49,53,56,57,60,63^ that investigated pregnancy intention as a determinant of pregnancy spacing, all found that unintended or mistimed pregnancies were associated with increased risks of short spacing between pregnancies.

Reproductive and obstetric history was examined under 11 sub-domains (fertility problems; previous perinatal loss; previous gestational diabetes; year of birth, parity, plurality, previous breech presentation, previous preterm birth, previous mode of delivery, perineal tear, and substance use during previous pregnancy). One study^52^ reported a minimal impact of fertility problems, previous history of miscarriage and a previous history of abortion on IBI. A previous perinatal loss was a significant predictor of a short IPI <24 months (aRR 0.36 95% CI 0.14–0.91).^37^ Having gestational diabetes mellitus in a previous pregnancy was a risk factor for short IPI (aOR 0.88 95% CI 0.82–0.94).^76^ Year of birth had a marginal influence on short IPI in one Norwegian historical study^50^ when comparing cohorts from 1967–1975 to 1976–1984. The influence of parity on pregnancy spacing was examined in eight studies,^48,49,54,55,57,58,60,62^ of which four^48,57,60,62^ reported that women with higher parity were at increased risk of subsequent shorter pregnancy spacing compared to women with lower parity. One study^50^ found that a previous preterm birth was a determinant for short IPI after a first birth (aHR 1.15 95% CI 1.13–1.15), but not after a second preterm birth. A previous breech birth^50,77^ was not a determinant of birth spacing, and two^50,59^ of the four studies^47,50,59,77^ that examined mode of delivery found that a previous caesarean section had a marginal influence of short pregnancy spacing. The three studies^52,60,62^ that examined substance use (smoking, alcohol, illicit drug use, and medication) during a previous pregnancy as a determinant of pregnancy spacing found no evidence of influence on birth spacing.

#### Relationships level

A total of eight studies^35,49–51,53,54,78,79^ investigated relationship level factors for short IPI under four sub-domains (partner characteristic, grandparent characteristics, family characteristics, and peer influence). Under partner characteristics, one study^49^ undertaken in Saudi Arabia found that the husband’s occupation (non-military positions vs. military officer) and beliefs of pregnancy spacing (don’t mind or encouraging vs. disagree strongly) were protective of short IBIs. A lower educational level attained by grandparents (≤10 educational years vs. completed college) was protective against shorter BTP <18 months (aOR 1.18 (95% CI 1. 4–2.5)) in an Australian study.^51^ Under the sub-domain of family characteristics, the previous death of a child was determined as a risk factor for shorter IPI in two Scandinavian studies.^35,50^ Studies that considered sex ratio found conflicting results, with one study^49^ finding a shorter IBI when the first-born child was male compared to one male: one female (aHR 1.50 95% CI 1.15–1.96), while another study^54^ in the UK found no association between sex ratio and IBIs. Intimate partner violence (IPV) was a risk factor for shorter pregnancy spacing in two studies,^53,79^ with one study^53^ reporting that IPV within 3 months of previous birth increased BTP intervals <24 months (OR 1.85 95% CI 1.18–2.88). Under peer influence, two studies^53,78^ determined that friends had an impact on pregnancy spacing, with one of these studies^53^ reporting that having friends that were adolescent mothers at the previous birth greatly increased the odds of a BTP <24 months (OR 1.52 95% CI 1.03–2.26). Another study^78^ found that adolescents who had a stronger relationship with their parents frequently attended church-based youth activities and were attached to church-based institutions were more likely to have reduced odds of a RRP.

#### Community level

Nine studies^34,36,42,43,48,56,68,80,81^ investigated community level factors for birth spacing under six sub-domains (access to primary care, family planning services, home-visiting program, prenatal care programmes and phone-based counselling). No continuity of primary care (aRR 1.16 95% CI1.14–1.19) was a risk factor for the RRP <12 months for women with intellectual and developmental disabilities in Cananda.^48^ Another Canadian study^80^ found that inadequate access to prenatal care was associated with increased odds of a short IPI <12 months (aOR 1.33 95% CI 1.12–1.43). A study^43^ of a phone-based counselling intervention conducted in the USA reported mixed results for short IBI (<12 months), finding that adolescent mothers aged 18–19 were less receptive to the intervention in preventing short IBI compared to mothers aged 15–17 years. Of the three studies^42,56,68^ that evaluated access to family planning services at a community level in the USA, two found^42,68^ that participation in family planning programmes was protective of shorter spacing between pregnancies. However, one study^56^ reported that 20% of participants in a home-visiting family planning program had an RRP within 24 months, significantly higher than the US national average of 11%. A further three studies^34,36,81^ considered home-visiting programmes at the community level, all of which found that access to home-visiting programme were protective to pregnancy spacing.

#### Societal level

Eight studies^31,44,48,55,57,82–84^ investigated societal factors for short IPIs of which six examined the influence of Medicaid enrolment on pregnancy spacing and birth outcomes in the USA. One study^57^ that investigated the prevalence and correlates of short IPI in the USA found that women who had Medicaid were more likely to have a mistimed (aOR 4.43 95% CI 3.07–6.39) or unintended (aOR 4.78 95% CI 3.36–6.86) pregnancy within an IPI of <18 months compared to women who had intended pregnancies. Supporting this finding, a 2018 study,^82^ which assessed the fulfilment of postpartum sterilisation requests between women with Medicaid versus women with private health insurance, found that women who had Medicaid access were more likely to have a shorter IPI <12 months (RR 2.57 95% CI 1.10–6.0). Two studies^83,84^ examined the impact of 2014 expansion of Medicaid to improve access to reproductive health care for low-income women in selected jurisdictions in the USA; however, the findings were inconsistent. The first^84^ reported a decrease in short IPIs <12 months in US states that participated in the Medicaid expansion while reporting an increase in short IPIs in non-participating states. By contrast, Liu et al.^83^ reported no significant association between short IPIs <12 months in jurisdictions that adopted the Medicaid expansion compared to non-expansion jurisdictions. Another study^44^ examined the association between a Medicaid program to reimburse hospitals for immediate LARC on infant health, with the findings suggesting that this policy change may have directly influenced the declining trends for short IBIs <15 months. Another study^31^ examined the influence of a religion at a societal level, finding that delivery at a Catholic hospital (which restricts access to comprehensive reproductive health services) was associated with an increased risk of short IPIs <6 months (OR 1.14 95% CI 1.07–1.21). The final study^48^ based in Canada found that the lack of access to adequate social assistance was a risk factor for RRP <12 months (aRR 1.71 95% CI 1.65–1.77) for women living with intellectual and developmental disabilities.

## Discussion

This systematic review examined the determinants of short IPIs in high-income countries through a socio-ecological lens. Our key finding was that most research focuses on individual level factors, with contraceptive use being the most commonly reported determinant across the included studies. Research that considers a too narrow view of influential factors precludes the development of effective multi-level and multi-pronged approaches that respond to the determinants of pregnancy spacing. Addressing these determinants is not only a public health imperative but also essential for upholding women's reproductive rights and autonomy. We identified factors at multiple levels of the socio-ecological model, though with diminishing evidence as we moved from individual to relationship, community, and societal levels. This multi-level understanding can provide health professionals and policy makers with information to guide the development of practice and policy to support optimum IPIs and reduce adverse outcomes associated with too short IPIs.

Most of the included studies reported factors at the individual level, reflecting a predominantly individualised approach to address short IPIs in high-income countries. The association between advanced maternal age (≥35 years) and shorter birth spacing identified in our included studies^37,52,58,59^ aligns with established demographic research^85^ which demonstrates the interaction of reproductive time pressures with a competing age-related decline in fecundity. This is particularly relevant given the well-documented global trend towards delayed childbearing in high-income countries. Conversely, younger women were at increased risk for short IPIs (<24 years)^37,46,48,55,57,61^, paralleling previous research^86^ which has found high rates of rapid repeat pregnancy among adolescents and young adults.

Drivers of this included unintended pregnancies,^87^ which can be prevented by appropriate use of contraception methods, which emerged as the most consistently researched factor for short IPIs, as identified by 14^32,52,55,56,62,63,65,68–74^ of the included studies. Long-acting reversible contraceptive methods, such as contraceptive implants and the intrauterine device, may be of interest to women who seek to delay subsequent pregnancies, as they require minimal user effort while providing effective contraceptive coverage.^55^ However, barriers to accessing contraception include affordability and healthcare availability, with a recent policy analysis^88^ reporting that these barriers disproportionately impact those already marginalised by factors, such as age,^55,57^ socio-economic status,^59,66^ race/ethnicity^33,55,57,62^, rurality,^48^ education level,^53,55,57,59,65^ and exposure to violence.^53,55,57,59,79,89^ Comprehensive contraceptive counselling during both the antenatal and postnatal periods remains critical for achieving optimal birth spacing while respecting women's reproductive autonomy and rights to make informed decisions about their bodies and families.

The socio-ecological model considers factors beyond the individual that influence health behaviours, including relationships, the community and societal policies. Women with more healthcare contact were more likely to have reduced risks of shorter pregnancy spacing.^34,36,42,43,48,56,68,80,81^ An exception was a US home-visiting programme^56^ that resulted in increased shorter IPIs among participants. The authors acknowledged that delaying recruitment impacted important family planning discussions, potentially missing a crucial postpartum opportunity. For low-income women residing in high-income countries without universal health coverage, there are increased barriers in accessing sexual and reproductive health care services.^88^ These barriers represent not only health inequities but also infringements on women's fundamental rights to reproductive healthcare. Six US-based studies^44,55,57,82–84^ demonstrated that access to Medicaid reduced risk for closer pregnancy spacing. Policies and legislation which remove barriers to ante- and postnatal programmes and increase access to family planning and contraception methods, reduce the burden of adverse outcomes associated with short IPIs on families in high-income countries.

Relationships with family, friends, and peers can have a strong impact on pregnancy spacing, though few studies in this review considered these factors. Partners had a strong influence on pregnancy spacing,^40,49^ suggesting that the active engagement of partners in ante- and postnatal programmes may reduce discordant beliefs around family planning. Religious values can influence IPIs, both positively^78^ by reducing rapid repeat pregnancy in young US adults, and negatively by prohibiting access to contraception.^31,49^ Peer influence, particularly on younger women^53^ and IPV^53,79^ are risk factors for short IPI, highlighting that some women may have reduced determination in pregnancy planning.^90^ However, targeted approaches can support women, such as identifying and supporting those at risk of IPV in early pregnancy.^91^ Overall, the impact of relationships underscores the need for comprehensive strategies that address social and cultural factors in pregnancy spacing while protecting women's rights to make reproductive decisions free from coercion or violence.

### Strengths and limitations

This systematic review has several strengths, including a comprehensive approach to examining determinants of short IPIs through a socio-ecological framework and its rigorous methodology following PRISMA guidelines. However, important limitations must be acknowledged. A key challenge relates to the heterogeneity of study designs included in our review. While cohort studies can provide evidence for causal relationships under certain conditions, establishing causality was not the primary aim of this review. The predominance of cohort studies (80% of included studies) provides valuable insights into associations between various factors and IPIs, though interpretation should consider potential unmeasured confounding factors. The few intervention studies included complemented this evidence but were limited in number.

Generalisability of our findings presents another limitation, as 67% of included studies were conducted in the US, potentially limiting applicability to other high-income countries with different healthcare systems, social policies, and cultural contexts. Sample characteristics across studies also varied considerably, with some focusing on specific populations (e.g. adolescents, women with particular health conditions), further constraining generalisability. This review was limited to English-language publications from high-income countries, which may have excluded relevant research conducted in other languages or healthcare settings with different resource contexts. For example, regional differences in reproductive healthcare access, particularly between countries with and without universal healthcare, likely influence the relative importance of various determinants.

Although there is clear evidence that shorter IPIs are associated with increased adverse outcomes, there remains no clear definition of what constitutes a short IPI, with the included studies accounted IPI ranging from ≤24 months^31–36^ to as short as ≤6 months.^31–37^ Consequently undertaking a meta-analysis was not possible. The heterogeneity in analytical approach, with most studies using categorical thresholds, fewer studies using continuous measures, and some employing survival analysis, limited direct comparisons between studies. Additionally, most studies required women to have had at least two pregnancies, introducing potential selection bias by excluding those who chose to have only one child or faced secondary infertility. Further challenges included inconsistent handling of pregnancy losses in IPI calculations, varying definitions of the start and end points for measurement (birth-to-conception vs. birth-to-birth), and limited ability to establish causality, particularly in retrospective studies. A more uniform definition of IPI would enable improved comparison of the influence of determinants across studies, with the potential for better informed future health interventions.

These methodological limitations may affect our conclusions by potentially overemphasising factors that are more easily measured in retrospective designs (e.g. demographic characteristics) while under-representing complex psychosocial or structural determinants that require more nuanced prospective assessment. Despite these limitations, this review provides valuable insights into the multi-level factors influencing IPIs in high-income countries and highlights important gaps for future research.

### Clinical implications

Our findings have important clinical implications for healthcare providers involved in women's reproductive health management, who are in a position to offer targeted education on optimal birth spacing during the antenatal and postpartum period. This education is especially important given the evidence of significant knowledge gaps among women regarding optimal birth spacing intervals, particularly for women identified at higher risk of short IPI (such as young women, women over 35, those with lower education levels, and those experiencing intimate partner violence).

Socio-economic barriers significantly impact contraceptive access and use; therefore, connecting women with available resources and assistance programs is an essential component of care. For adolescent mothers specifically, our review points to the powerful influence of peer relationships on birth spacing decisions. Establishing peer support networks and specialised programmes for young mothers represents an evidence-based approach to addressing the unique needs of this population.

Access to effective and appropriate contraception is a priority. However, this approach should occur in an environment that respects women's preferences for family planning, within a rights-based framework that emphasises informed decision-making and reproductive autonomy. These systems that service women need to be inclusive, considering the individual and relationships, including partner involvement in family planning discussions, while also acknowledging spiritual and cultural influences on birth spacing decisions.

### Future research directions

We found that partner influence was a significant factor in birth spacing, yet research examining relationship dynamics in reproductive decision-making remains limited. Studies exploring how power dynamics, communication patterns, and partner preferences affect birth spacing would enhance our understanding of these interpersonal factors. Additionally, cultural and religious influences on family planning decisions were also identified as important but understudied factors that warrant more dedicated research.

Policy research also needs greater attention, particularly studies focusing on evaluating how different healthcare financing models and access policies impact birth spacing. There are future opportunities potentially for the trialling of technology-based interventions, with the application of mobile health, telehealth counselling, and digital education tools to support women's reproductive autonomy across various settings.

Overall, there is a critical need for well-designed prospective longitudinal studies that can better inform causal relationships between potential determinants and birth spacing outcomes, moving beyond the predominantly observational evidence that is currently available. Specifically, studies that evaluate integrated approaches, considering factors at multiple levels of the socio-ecological model may provide valuable insights into how to more effectively promote optimal birth spacing.

## Conclusion

This was the first review to take a multi-dimensional approach to identifying the factors that influence birth spacing in high-income countries. By applying the socio-ecological model, we were able to identify a range of factors that drive shorter IPIs, providing a theoretical informed knowledge base for policy makers and health professionals to reconsider current practices, advocate for, and develop policies to improve birth spacing in high-income countries. Based on our comprehensive analysis using the socio-ecological model, we recommend increased research into factors beyond the individual factors that influence IPIs, so this issue can be more comprehensively understood. There should be consideration of education to inform women, their families, and communities, of the associated risks of short IPIs, so that they can make informed choices about pregnancies; and policies and practices that support access to reproductive services and contraception within communities. The factors identified within this review are a step towards providing policy makers, maternal health service providers, and public health practitioners with information to help inform interventions to promote healthy IPIs; with the long-term aim of reducing or eliminating any disparities that contribute to short IPIs within high-income countries.

## Author contributions

Conceptualisation: JD, DF, GP, GAT. Methodology: JD, DF, GAT. Formal analysis: JD, DF, BK, DGB, GD, KBM, GAT. Writing – original draft: JD, GAT. Writing – review and editing: JD, JJ, GP, BK, DGB, GD, ATG, KBM, SDN, AR, GAT. Validation: JD, DF, JJ, GP, BK, DGP, GD, GAT. Visualisation: JD, GAT. Supervision: JD, GAT. Project administration: JD, DF, GAT. Funding: GP, GAT.

## Supplementary Material

Supplementary data

## Provenance statement

The article was not commissioned and went through external peer review.
