# Supplementary material for: Determinants of short interpregnancy intervals in high-income countries: a systematic review
Source: Sex Reprod Health Matters. 2025 Sep 1;33(1):2545699. doi: 10.1080/26410397.2025.2545699 (PMC12498375; doi:10.1080/26410397.2025.2545699)
Supplement: Supplementary data [file ZRHM_A_2545699_SM2224.docx]

**A systematic review of the determinants of short interpregnancy intervals in high-income countries**

**Authors:** Jennifer Dunne, Damien Foo, Jonine Jancey, Gavin Pereira, Bereket Kefale, Daniel G Belay, Gursimran Dhamrait, Amanuel T Gebremedhin, , Kalayu Bhrane Mruts, Sylvester D Nyadanu, Aditi Roy, Gizachew A Tessema

**Supplementary materials content:**

[Supplementary table 1. Preferred Reporting for Systematic Review and Meta-Analysis (PRISMA) 2020 completed checklist 2](#_Toc164491826)

[Supplementary table 2. Systematic search of the literature related to diabetic and hypertensive disorders following an early pregnancy loss by search engine. 5](#_Toc164491827)

[Supplementary table 3. Records excluded at full-text screening with reasons (n=170) 12](#_Toc164491828)

[Supplementary table 4. Summary of the characteristics of studies reporting determinants of interpregnancy intervals in high-income countries (n=55). 23](#_Toc164491829)

[Supplementary table 5. JBI Risk of bias assessment for cross-sectional studies (n=6) 74](#_Toc164491830)

[Supplementary table 6. JBI Risk of bias assessment for case-control studies (n=2) 75](#_Toc164491831)

[Supplementary table 7. JBI Risk of bias assessment for cohort studies (n=44) 76](#_Toc164491832)

[Supplementary table 8. Cochrane risk of bias tool assessment for intervention studies (n=3) 80](#_Toc164491833)

Supplementary table 1. Preferred Reporting for Systematic Review and Meta-Analysis (PRISMA) 2020 completed checklist

| **Section and Topic** | **Item #** | **Checklist item** | **Location where item is reported** |
| --- | --- | --- | --- |
| **TITLE** | | |  |
| Title | 1 | Identify the report as a systematic review. | Pg 1, line 1 |
| **ABSTRACT** | | |  |
| Abstract | 2 | See the PRISMA 2020 for Abstracts checklist. | Pg 2, line 1 |
| **INTRODUCTION** | | |  |
| Rationale | 3 | Describe the rationale for the review in the context of existing knowledge. | Pg 3, line 26 |
| Objectives | 4 | Provide an explicit statement of the objective(s) or question(s) the review addresses. | Pg 4, line 9 |
| **METHODS** | | |  |
| Eligibility criteria | 5 | Specify the inclusion and exclusion criteria for the review and how studies were grouped for the syntheses. | Pg 4, line 27 |
| Information sources | 6 | Specify all databases, registers, websites, organisations, reference lists and other sources searched or consulted to identify studies. Specify the date when each source was last searched or consulted. | Pg 4, line 18 |
| Search strategy | 7 | Present the full search strategies for all databases, registers and websites, including any filters and limits used. | Supp T S2 |
| Selection process | 8 | Specify the methods used to decide whether a study met the inclusion criteria of the review, including how many reviewers screened each record and each report retrieved, whether they worked independently, and if applicable, details of automation tools used in the process. | Pg 5, line 4 |
| Data collection process | 9 | Specify the methods used to collect data from reports, including how many reviewers collected data from each report, whether they worked independently, any processes for obtaining or confirming data from study investigators, and if applicable, details of automation tools used in the process. | Pg 5, line 11 |
| Data items | 10a | List and define all outcomes for which data were sought. Specify whether all results that were compatible with each outcome domain in each study were sought (e.g. for all measures, time points, analyses), and if not, the methods used to decide which results to collect. | Pg 5, line 20 |
|  | 10b | List and define all other variables for which data were sought (e.g. participant and intervention characteristics, funding sources). Describe any assumptions made about any missing or unclear information. | Pg 5, line 13 |
| Study risk of bias assessment | 11 | Specify the methods used to assess risk of bias in the included studies, including details of the tool(s) used, how many reviewers assessed each study and whether they worked independently, and if applicable, details of automation tools used in the process. | Pg 5, line 16 |
| Effect measures | 12 | Specify for each outcome the effect measure(s) (e.g. risk ratio, mean difference) used in the synthesis or presentation of results. | NA |
| Synthesis methods | 13a | Describe the processes used to decide which studies were eligible for each synthesis (e.g. tabulating the study intervention characteristics and comparing against the planned groups for each synthesis (item #5)). | Pg 5, line 30 |
|  | 13b | Describe any methods required to prepare the data for presentation or synthesis, such as handling of missing summary statistics, or data conversions. | NA |
|  | 13c | Describe any methods used to tabulate or visually display results of individual studies and syntheses. | Pg 5, line 31 |
|  | 13d | Describe any methods used to synthesize results and provide a rationale for the choice(s). If meta-analysis was performed, describe the model(s), method(s) to identify the presence and extent of statistical heterogeneity, and software package(s) used. | NA |
|  | 13e | Describe any methods used to explore possible causes of heterogeneity among study results (e.g. subgroup analysis, meta-regression). | NA |
|  | 13f | Describe any sensitivity analyses conducted to assess robustness of the synthesized results. | NA |
| Reporting bias assessment | 14 | Describe any methods used to assess risk of bias due to missing results in a synthesis (arising from reporting biases). | NA |
| Certainty assessment | 15 | Describe any methods used to assess certainty (or confidence) in the body of evidence for an outcome. | NA |
| **RESULTS** | | |  |
| Study selection | 16a | Describe the results of the search and selection process, from the number of records identified in the search to the number of studies included in the review, ideally using a flow diagram. | Figure 1 |
|  | 16b | Cite studies that might appear to meet the inclusion criteria, but which were excluded, and explain why they were excluded. | Supp T S3 |
| Study characteristics | 17 | Cite each included study and present its characteristics. | Supp T S4 |
| Risk of bias in studies | 18 | Present assessments of risk of bias for each included study. | Supp T S5-S8 |
| Results of individual studies | 19 | For all outcomes, present, for each study: (a) summary statistics for each group (where appropriate) and (b) an effect estimate and its precision (e.g. confidence/credible interval), ideally using structured tables or plots. | Supp T S4 |
| Results of syntheses | 20a | For each synthesis, briefly summarise the characteristics and risk of bias among contributing studies. | Pg 6, line 18 |
|  | 20b | Present results of all statistical syntheses conducted. If meta-analysis was done, present for each the summary estimate and its precision (e.g. confidence/credible interval) and measures of statistical heterogeneity. If comparing groups, describe the direction of the effect. | Pg6-8 |
|  | 20c | Present results of all investigations of possible causes of heterogeneity among study results. | NA |
|  | 20d | Present results of all sensitivity analyses conducted to assess the robustness of the synthesized results. | NA |
| Reporting biases | 21 | Present assessments of risk of bias due to missing results (arising from reporting biases) for each synthesis assessed. | NA |
| Certainty of evidence | 22 | Present assessments of certainty (or confidence) in the body of evidence for each outcome assessed. | NA |
| **DISCUSSION** | | |  |
| Discussion | 23a | Provide a general interpretation of the results in the context of other evidence. | Pg 11, line 4 |
|  | 23b | Discuss any limitations of the evidence included in the review. | Pg 13, line 5 |
|  | 23c | Discuss any limitations of the review processes used. | Pg 13, line 10 |
|  | 23d | Discuss implications of the results for practice, policy, and future research. | Pg 13, line 19 |
| **OTHER INFORMATION** | | |  |
| Registration and protocol | 24a | Provide registration information for the review, including register name and registration number, or state that the review was not registered. | Pg 4, line 16 |
|  | 24b | Indicate where the review protocol can be accessed, or state that a protocol was not prepared. | NA |
|  | 24c | Describe and explain any amendments to information provided at registration or in the protocol. | NA |
| Support | 25 | Describe sources of financial or non-financial support for the review, and the role of the funders or sponsors in the review. | Pg 14 |
| Competing interests | 26 | Declare any competing interests of review authors. | Pg 14 |
| Availability of data, code and other materials | 27 | Report which of the following are publicly available and where they can be found: template data collection forms; data extracted from included studies; data used for all analyses; analytic code; any other materials used in the review. | NA |

*From:*  Page MJ, McKenzie JE, Bossuyt PM, Boutron I, Hoffmann TC, Mulrow CD, et al. The PRISMA 2020 statement: an updated guideline for reporting systematic reviews. BMJ 2021;372:n71. doi: 10.1136/bmj.n71

Supplementary table 2. Systematic search of the literature related to diabetic and hypertensive disorders following an early pregnancy loss by search engine.

| **Search engine** | **Search string** |
| --- | --- |
| CINAHL Plus | (MH ("Population Characteristics+" OR "Demography+" OR "Family Planning+" OR "Reproduction+" OR "Reproductive Control Agents" OR "Contraception+" OR "Contraceptive Devices+" OR "Socioeconomic Factors+" OR "Income+" OR "Behavioral Sciences+" OR "Behavior+" OR "Motivation+" OR "Psychology, Social+" OR "Social Sciences" OR "Family+" OR "Marital Status+" OR "Maternal Age+" OR "Paternal Age+" OR "Religion and Religions+" OR "Lifestyle+" OR "Health" OR "Health Facilities+" OR "Health Manpower+" OR "Health Promotion+" OR "Health Services+" OR "Health and Welfare Planning+" OR "Health Services Administration+" OR "Comorbidity+" OR "Morbidity" OR "Health Status+" OR "Education+") OR MH ("Population Characteristics+" OR "Demography+" OR "Family Planning+" OR "Reproduction+" OR "Reproductive Control Agents" OR "Contraception+" OR "Contraceptive Devices+" OR "Socioeconomic Factors+" OR "Income+" OR "Behavioral Sciences+" OR "Behavior+" OR "Motivation+" OR "Psychology, Social+" OR "Social Sciences" OR "Family+" OR "Marital Status+" OR "Maternal Age+" OR "Paternal Age+" OR "Religion and Religions+" OR "Lifestyle+" OR "Health" OR "Health Facilities+" OR "Health Manpower+" OR "Health Promotion+" OR "Health Services+" OR "Health and Welfare Planning+" OR "Health Services Administration+" OR "Comorbidity+" OR "Morbidity" OR "Health Status+" OR "Education+") OR AB ("Demograph* OR "Family Plan*" OR "Contracept*" OR "Birth control" OR "Socioeconomic" OR "Income" OR "Financ*" OR "Education*" OR "Academic" OR "Behavio*" OR "Psycholog*" OR "Lifestyle" OR )"Marital status" OR "Marriage" OR "Maternal" OR "Mother's" OR "Paternal" OR "Father's" OR "Parental" W0 "Age" OR "Religio*" OR "Lifestyle" OR "Comorbid*"OR "Co-morbid*" OR "Morbid*"OR "Determinant" OR "Factor*" OR "Influenc*"OR "Barrier" OR "Caus*" OR "Predict*" OR "Characteristic" OR "Enabl*" OR "Encourag*" OR "Motiv*" OR "Help*"OR "Indicat*"OR "Drive*"OR "Decision") ) AND ((MH "Birth Intervals+") OR TI (Birth or "Birth to birth" or "Birth to conception" or "Birth to delivery" or "Birth to pregnancy" or "Conception" or "Delivery" or "Delivery to conception" or "Delivery to delivery" or "Interbirth" or "Inter-birth" or "Inter birth" or "Interconception" or "Inter-conception" or "Inter conception" or "Interdelivery" or "Inter-delivery" or "Inter delivery" or "Interpregnancy" or "Inter-pregnancy" or "Inter pregnancy" or "Pregnancy" or "Time to birth") W0 ("Spacing" or "Interval")) OR AB ("Birth" or "Birth to birth" or "Birth to conception" or "Birth to delivery" or "Birth to pregnancy" or "Conception" or "Delivery" or "Delivery to conception" or "Delivery to delivery" or "Interbirth" or "Inter-birth" or "Inter birth" or "Interconception" or "Inter-conception" or "Inter conception" or "Interdelivery" or "Inter-delivery" or "Inter delivery" or "Interpregnancy" or "Inter-pregnancy" or "Inter pregnancy" or "Pregnancy" or "Time to birth") WO ("Spacing" or "Interval"))) AND ((MH "Developed Countries+") OR ((TI "High income") OR (TI "High-income") OR (TI "Developed") OR (TI "Industrialised") OR (TI "Industrialized") W0 (TI "Countr*") OR (TI "Nation")) OR ((AB "High income") OR (AB "High-income") OR (AB "Developed") OR (AB "Industrialised") OR (AB "Industrialized") W0 (AB "Countr*") OR (AB "Nation")) OR (TI ("Aruba" or "Andorra" or "United Arab Emirates" or "Emirates" or "Antigua and Barbuda" or "Antigua" or "Barbuda" or "Australia" or "Austria" or "Belgium" or "Bahrain" or "Bahamas" or "Bermuda" or "Barbados" or "Brunei" or "Canada" or "Switzerland" or "Channel Islands" or "Chile" or "Curaçao" or "Cayman Islands" or "Cyprus" or "Czech Republic" or "Germany" or "Denmark" or "Spain" or "Estonia" or "Finland" or "France" or "Faroe Islands" or "United Kingdom" or "Britain" or "Northern Ireland" or "England" or "Scotland" or "Wales" or "Gibraltar" or "Greece" or "Greenland" or "Guam" or "Hong Kong" or "Croatia" or "Hungary" or "Isle of Man" or "Ireland" or "Iceland" or "Israel" or "Italy" or "Japan" or "St. Kitts and Nevis" or "Saint Kitts and Nevis" or "St. Kitts and Nevis" or "Saint Kitts and Nevis" or "St. Kitts" or "Nevis" or "Republic of Korea" or "South Korea" or "Kuwait" or "Liechtenstein" or "Lithuania" or "Luxembourg" or "Latvia" or "Macao" or "St. Martin" or "Saint Martin" or "Saint-Martin" or "Monaco" or "Malta" or "Northern Mariana Islands" or "New Caledonia" or "Netherlands" or "Holland" or "Norway" or "Nauru" or "New Zealand" or "Oman" or "Palau" or "Poland" or "Puerto Rico" or "Portugal" or "French Polynesia" or "Qatar" or "Saudi Arabia" or "Singapore" or "San Marino" or "Slovak Republic" or "Slovenia" or "Sweden" or "Sint Maarten" or "Seychelles" or "Turks and Caicos Islands" or "Trinidad and Tobago" or "Trinidad" or "Tobago" or "Taiwan" or "Uruguay" or "United States" or "British Virgin Islands" or "Virgin Islands")) OR (AB ("Aruba" or "Andorra" or "United Arab Emirates" or "Emirates" or "Antigua and Barbuda" or "Antigua" or "Barbuda" or "Australia" or "Austria" or "Belgium" or "Bahrain" or "Bahamas" or "Bermuda" or "Barbados" or "Brunei" or "Canada" or "Switzerland" or "Channel Islands" or "Chile" or "Curaçao" or "Cayman Islands" or "Cyprus" or "Czech Republic" or "Germany" or "Denmark" or "Spain" or "Estonia" or "Finland" or "France" or "Faroe Islands" or "United Kingdom" or "Britain" or "Northern Ireland" or "England" or "Scotland" or "Wales" or "Gibraltar" or "Greece" or "Greenland" or "Guam" or "Hong Kong" or "Croatia" or "Hungary" or "Isle of Man" or "Ireland" or "Iceland" or "Israel" or "Italy" or "Japan" or "St. Kitts and Nevis" or "Saint Kitts and Nevis" or "St. Kitts and Nevis" or "Saint Kitts and Nevis" or "St. Kitts" or "Nevis" or "Republic of Korea" or "South Korea" or "Kuwait" or "Liechtenstein" or "Lithuania" or "Luxembourg" or "Latvia" or "Macao" or "St. Martin" or "Saint Martin" or "Saint-Martin" or "Monaco" or "Malta" or "Northern Mariana Islands" or "New Caledonia" or "Netherlands" or "Holland" or "Norway" or "Nauru" or "New Zealand" or "Oman" or "Palau" or "Poland" or "Puerto Rico" or "Portugal" or "French Polynesia" or "Qatar" or "Saudi Arabia" or "Singapore" or "San Marino" or "Slovak Republic" or "Slovenia" or "Sweden" or "Sint Maarten" or "Seychelles" or "Turks and Caicos Islands" or "Trinidad and Tobago" or "Taiwan" or "Uruguay" or "United States" or "British Virgin Islands" or "Virgin Islands"))) |
| Ovid (EMBASE) | (exp "Population"/ or exp "Demography"/ or "Demograph*".ti,ab. or exp "Family Planning"/ or "Family Plan*".ti,ab. Or exp "Reproduction"/ or exp "Birth Control" or ("Contracept*" or "Birth control").ti,ab. OR exp "Socioeconomics"/ or exp "Social Status"/ or exp "Income"/ or ("Socioeconomic" or "Income" or "Financ*").ti,ab. or exp "Education"/ or exp "Academic Achievement"/ or exp "Academic Success"/ or ("Education*" or "Academic").ti,ab. OR exp "Psychiatry" or exp "Psychology"/ or exp "Behavior"/ or exp "Behavioral Science"/ or exp "Sociology"/ OR exp "Marital Status"/ or exp "Maternal age"/ or exp "Paternal age"/ or exp "Parental age"/ or (("Maternal" or "Mother's" or "Paternal" or "Father's" or "Parental") adj ("Age")).ti,ab. OR exp "Religion"/ or exp "Lifestyle"/ or "Religion".ti,ab. or "Lifestyle".ti,ab. OR exp "Health"/ or exp "Health Care Utilization"/ or exp "Health Care Delivery"/ OR exp "Comorbidity"/ or exp "Morbidity"/ or ("Comorbid*" or Comorbid*" "Co-morbid*" or "Morbid*").ti,ab. OR ("Determinant" or "Factor*" or "Influenc*" or "Barrier" or "Caus*" or "Predict*" or "Characteristic" or "Enabl*" or "Encourag*" or "Motiv*" or "Help*" or "Indicat*" or "Drive*" or "Decision").ti,ab. ) AND ((("Birth" or "Birth to birth" or "Birth to conception" or "Birth to delivery" or "Birth to pregnancy" or "Conception" or "Delivery" or "Delivery to conception" or "Delivery to delivery" or "Interbirth" or "Inter-birth" or "Inter birth" or "Interconception" or "Inter-conception" or "Inter conception" or "Interdelivery" or "Inter-delivery" or "Inter delivery" or "Interpregnancy" or "Inter-pregnancy" or "Inter pregnancy" or "Pregnancy" or "Time to birth") adj ("Spacing" or "Interval")).ti,ab. OR ("Interval* between birth*" or "Repeat pregnanc*").ti,ab.) AND (exp "High Income Country"/ or exp "Developed Country"/ OR (("High income" or "High-income" or "Developed" or "Industrialised" or "Industrialized") adj ("Countr*" or "Nation")).ti,ab. OR ("Aruba" or "Andorra" or "United Arab Emirates" or "Emirates" or "Antigua and Barbuda" or "Antigua" or "Barbuda" or "Australia" or "Austria" or "Belgium" or "Bahrain" or "Bahamas" or "Bermuda" or "Barbados" or "Brunei" or "Canada" or "Switzerland" or "Channel Islands" or "Chile" or "Curaçao" or "Cayman Islands" or "Cyprus" or "Czech Republic" or "Germany" or "Denmark" or "Spain" or "Estonia" or "Finland" or "France" or "Faroe Islands" or "United Kingdom" or "Britain" or "Northern Ireland" or "England" or "Scotland" or "Wales" or "Gibraltar" or "Greece" or "Greenland" or "Guam" or "Hong Kong" or "Croatia" or "Hungary" or "Isle of Man" or "Ireland" or "Iceland" or "Israel" or "Italy" or "Japan" or "St. Kitts and Nevis" or "Saint Kitts and Nevis" or "St. Kitts and Nevis" or "Saint Kitts and Nevis" or "St. Kitts" or "Nevis" or "Republic of Korea" or "South Korea" or "Kuwait" or "Liechtenstein" or "Lithuania" or "Luxembourg" or "Latvia" or "Macao" or "St. Martin" or "Saint Martin" or "Saint-Martin" or "Monaco" or "Malta" or "Northern Mariana Islands" or "New Caledonia" or "Netherlands" or "Holland" or "Norway" or "Nauru" or "New Zealand" or "Oman" or "Palau" or "Poland" or "Puerto Rico" or "Portugal" or "French Polynesia" or "Qatar" or "Saudi Arabia" or "Singapore" or "San Marino" or "Slovak Republic" or "Slovenia" or "Sweden" or "Sint Maarten" or "Seychelles" or "Turks and Caicos Islands" or "Trinidad and Tobago" or "Trinidad" or "Tobago" or "Taiwan" or "Uruguay" or "United States" or "British Virgin Islands" or "Virgin Islands").ti,ab.) |
| Ovid (MEDLINE) | (exp "Population Characteristics"/ or exp "Demography"/ or exp "Family Planning Services"/ or ("Demograph*" or "Family Plan*").ti,ab. OR exp "Population Characteristics"/ or exp "Demography"/ or exp "Family Planning Services"/ or ("Demograph*" or "Family Plan*").ti,ab. OR exp "Socioeconomic Factors"/ or ("Socioeconomic" or "Income" or "Financ*").ti,ab. or exp "Education"/ or ("Education*" or "Academic").ti,ab. OR exp "Psychiatry and Psychology"/ or exp "Social Sciences"/ OR exp "Marital Status"/ or ("Marital status" or "Married").ti,ab. or (("Maternal" or "Mother's" or "Paternal" or "Father's" or "Parental") adj ("Age")).ti,ab. OR exp "Religion"/ or ("Religion" or "Lifestyle").ti,ab. OR exp "Health"/ or exp "Delivery of Health Care"/ OR exp "Comorbidity"/ or exp "Morbidity"/ or ("Comorbid*" or Comorbid*" "Co-morbid*" or "Morbid*").ti,ab. OR ("Determinant" or "Factor*" or "Influenc*" or "Barrier" or "Caus*" or "Predict*" or "Characteristic" or "Enabl*" or "Encourag*" or "Motiv*" or "Help*" or "Indicat*" or "Drive*" or "Decision").ti,ab.) AND (exp "Birth Intervals"/ OR (("Birth" or "Birth to birth" or "Birth to conception" or "Birth to delivery" or "Birth to pregnancy" or "Conception" or "Delivery" or "Delivery to conception" or "Delivery to delivery" or "Interbirth" or "Inter-birth" or "Inter birth" or "Interconception" or "Inter-conception" or "Inter conception" or "Interdelivery" or "Inter-delivery" or "Inter delivery" or "Interpregnancy" or "Inter-pregnancy" or "Inter pregnancy" or "Pregnancy" or "Time to birth") adj ("Spacing" or "Interval")).ti,ab. OR ("Interval* between birth*" or "Repeat pregnanc*").ti,ab.) AND ("Developed Countries"/ OR (("High income" or "High-income" or "Developed" or "Industrialised" or "Industrialized") adj ("Countr*" or "Nation")).ti,ab. OR ("Aruba" or "Andorra" or "United Arab Emirates" or "Emirates" or "Antigua and Barbuda" or "Antigua" or "Barbuda" or "Australia" or "Austria" or "Belgium" or "Bahrain" or "Bahamas" or "Bermuda" or "Barbados" or "Brunei" or "Canada" or "Switzerland" or "Channel Islands" or "Chile" or "Curaçao" or "Cayman Islands" or "Cyprus" or "Czech Republic" or "Germany" or "Denmark" or "Spain" or "Estonia" or "Finland" or "France" or "Faroe Islands" or "United Kingdom" or "Britain" or "Northern Ireland" or "England" or "Scotland" or "Wales" or "Gibraltar" or "Greece" or "Greenland" or "Guam" or "Hong Kong" or "Croatia" or "Hungary" or "Isle of Man" or "Ireland" or "Iceland" or "Israel" or "Italy" or "Japan" or "St. Kitts and Nevis" or "Saint Kitts and Nevis" or "St. Kitts and Nevis" or "Saint Kitts and Nevis" or "St. Kitts" or "Nevis" or "Republic of Korea" or "South Korea" or "Kuwait" or "Liechtenstein" or "Lithuania" or "Luxembourg" or "Latvia" or "Macao" or "St. Martin" or "Saint Martin" or "Saint-Martin" or "Monaco" or "Malta" or "Northern Mariana Islands" or "New Caledonia" or "Netherlands" or "Holland" or "Norway" or "Nauru" or "New Zealand" or "Oman" or "Palau" or "Poland" or "Puerto Rico" or "Portugal" or "French Polynesia" or "Qatar" or "Saudi Arabia" or "Singapore" or "San Marino" or "Slovak Republic" or "Slovenia" or "Sweden" or "Sint Maarten" or "Seychelles" or "Turks and Caicos Islands" or "Trinidad and Tobago" or "Trinidad" or "Tobago" or "Taiwan" or "Uruguay" or "United States" or "British Virgin Islands" or "Virgin Islands").ti,ab.) |
| Ovid (PsychINFO) | (exp "Population" or exp "Family Planning"/ or ("Demograph*" or "Family Plan*").ti,ab. OR exp "Sexual Reproduction"/ or exp "Reproductive Technology"/ or exp "Birth Control"/ or ("Contracept*" or "Birth control").ti,ab. OR exp "Social Sciences"/ or exp "Social Class"/ or exp "Social Equity"/ or exp "Social Equality"/ or exp "Social Disadvantage" or ("Socioeconomic" or "Income" or "Financ*").ti,ab. or exp "Education"/ or ("Education*" or "Academic").ti,ab. OR exp "Psychiatry" or exp "Psychology"/ or exp "Behavior"/ or exp "Behavioral Sciences"/ or exp "Social Sciences"/ OR exp "Parental Characteristics"/ or "Marital Status"/ or ("Marital status" or "Married").ti,ab. or (("Maternal" or "Mother's" or "Paternal" or "Father's" or "Parental") adj ("Age")).ti,ab. OR exp "Religion"/ or ("Religion" or "Lifestyle").ti,ab. OR exp "Health" or exp "Health Care Services"/ OR exp "Comorbidity"/ or exp "Morbidity"/ or ("Comorbid*" or Comorbid*" "Co-morbid*" or "Morbid*").ti,ab. OR ("Determinant" or "Factor*" or "Influenc*" or "Barrier" or "Caus*" or "Predict*" or "Characteristic" or "Enabl*" or "Encourag*" or "Motiv*" or "Help*" or "Indicat*" or "Drive*" or "Decision").ti,ab.) AND ((("Birth" or "Birth to birth" or "Birth to conception" or "Birth to delivery" or "Birth to pregnancy" or "Conception" or "Delivery" or "Delivery to conception" or "Delivery to delivery" or "Interbirth" or "Inter-birth" or "Inter birth" or "Interconception" or "Inter-conception" or "Inter conception" or "Interdelivery" or "Inter-delivery" or "Inter delivery" or "Interpregnancy" or "Inter-pregnancy" or "Inter pregnancy" or "Pregnancy" or "Time to birth") adj ("Spacing" or "Interval")).ti,ab. OR ("Interval* between birth*" or "Repeat pregnanc*").ti,ab.) AND ("Developed Countries"/ OR (("High income" or "High-income" or "Developed" or "Industrialised" or "Industrialized") adj ("Countr*" or "Nation")).ti,ab. OR ("Aruba" or "Andorra" or "United Arab Emirates" or "Emirates" or "Antigua and Barbuda" or "Antigua" or "Barbuda" or "Australia" or "Austria" or "Belgium" or "Bahrain" or "Bahamas" or "Bermuda" or "Barbados" or "Brunei" or "Canada" or "Switzerland" or "Channel Islands" or "Chile" or "Curaçao" or "Cayman Islands" or "Cyprus" or "Czech Republic" or "Germany" or "Denmark" or "Spain" or "Estonia" or "Finland" or "France" or "Faroe Islands" or "United Kingdom" or "Britain" or "Northern Ireland" or "England" or "Scotland" or "Wales" or "Gibraltar" or "Greece" or "Greenland" or "Guam" or "Hong Kong" or "Croatia" or "Hungary" or "Isle of Man" or "Ireland" or "Iceland" or "Israel" or "Italy" or "Japan" or "St. Kitts and Nevis" or "Saint Kitts and Nevis" or "St. Kitts and Nevis" or "Saint Kitts and Nevis" or "St. Kitts" or "Nevis" or "Republic of Korea" or "South Korea" or "Kuwait" or "Liechtenstein" or "Lithuania" or "Luxembourg" or "Latvia" or "Macao" or "St. Martin" or "Saint Martin" or "Saint-Martin" or "Monaco" or "Malta" or "Northern Mariana Islands" or "New Caledonia" or "Netherlands" or "Holland" or "Norway" or "Nauru" or "New Zealand" or "Oman" or "Palau" or "Poland" or "Puerto Rico" or "Portugal" or "French Polynesia" or "Qatar" or "Saudi Arabia" or "Singapore" or "San Marino" or "Slovak Republic" or "Slovenia" or "Sweden" or "Sint Maarten" or "Seychelles" or "Turks and Caicos Islands" or "Trinidad and Tobago" or "Trinidad" or "Tobago" or "Taiwan" or "Uruguay" or "United States" or "British Virgin Islands" or "Virgin Islands").ti,ab.) |
| Proquest | (MESH("Population" or "Demography" or "Family Planning") or TI,AB("Demograph*" or "Family Plan*") OR MESH("Reproduction" or "Reproductive Techniques" or "Contraception" or "Contraceptive Agents") or TI,AB("Contracept*") or TI,AB("Birth control") OR MESH("Socioeconomic Factors" or "Social Status" or "Social Class") or TI,AB("Socioeconomic" or "Income" or "Financ*") or MESH("Education" or "Educational Status" or "Academic" or "Academic Success") or TI,AB("Education*" or "Academic") OR MESH("Psychiatry" or "Psychology" or "Behavior" or "Behavioral Sciences" or "Sociology" or "Social Sciences") OR MESH("Marital Status") or TI,AB("Marital status" or "Married") or MESH("Maternal Age" or "Paternal Age") or TI,AB(("Maternal" or "Mother's" or "Paternal" or "Father's" or "Parental") NEAR/0 ("Age")) OR MESH("Religion") or TI,AB("Religion" or "Lifestyle") OR MESH("Health") OR MESH("Comorbidity" or "Morbidity") or TI,AB("Comorbid*" or "Comorbid*" or "Co-morbid*" or "Morbid*") OR TI,AB("Determinant" or "Factor*" or "Influenc*" or "Barrier" or "Caus*" or "Predict*" or "Characteristic" or "Enabl*" or "Encourag*" or "Motiv*" or "Help*" or "Indicat*" or "Drive*" or "Decision")) AND (MESH("Birth Intervals") OR TI,AB(("Birth" or "Birth to birth" or "Birth to conception" or "Birth to delivery" or "Birth to pregnancy" or "Conception" or "Delivery" or "Delivery to conception" or "Delivery to delivery" or "Interbirth" or "Inter-birth" or "Inter birth" or "Interconception" or "Inter-conception" or "Inter conception" or "Interdelivery" or "Inter-delivery" or "Inter delivery" or "Interpregnancy" or "Inter-pregnancy" or "Inter pregnancy" or "Pregnancy" or "Time to birth") NEAR/0 ("Spacing" or "Interval")) OR TI,AB("Interval* between birth*" or "Repeat pregnanc*")) AND (MESH("Developed Countries") OR TI,AB(("High income" or "High-income" or "Developed" or "Industrialised" or "Industrialized") NEAR/0 ("Countr*" or "Nation")) OR TI,AB("Aruba" or "Andorra" or "United Arab Emirates" or "Emirates" or "Antigua and Barbuda" or "Antigua" or "Barbuda" or "Australia" or "Austria" or "Belgium" or "Bahrain" or "Bahamas" or "Bermuda" or "Barbados" or "Brunei" or "Canada" or "Switzerland" or "Channel Islands" or "Chile" or "Curaçao" or "Cayman Islands" or "Cyprus" or "Czech Republic" or "Germany" or "Denmark" or "Spain" or "Estonia" or "Finland" or "France" or "Faroe Islands" or "United Kingdom" or "Britain" or "Northern Ireland" or "England" or "Scotland" or "Wales" or "Gibraltar" or "Greece" or "Greenland" or "Guam" or "Hong Kong" or "Croatia" or "Hungary" or "Isle of Man" or "Ireland" or "Iceland" or "Israel" or "Italy" or "Japan" or "St. Kitts and Nevis" or "Saint Kitts and Nevis" or "St. Kitts and Nevis" or "Saint Kitts and Nevis" or "St. Kitts" or "Nevis" or "Republic of Korea" or "South Korea" or "Kuwait" or "Liechtenstein" or "Lithuania" or "Luxembourg" or "Latvia" or "Macao" or "St. Martin" or "Saint Martin" or "Saint-Martin" or "Monaco" or "Malta" or "Northern Mariana Islands" or "New Caledonia" or "Netherlands" or "Holland" or "Norway" or "Nauru" or "New Zealand" or "Oman" or "Palau" or "Poland" or "Puerto Rico" or "Portugal" or "French Polynesia" or "Qatar" or "Saudi Arabia" or "Singapore" or "San Marino" or "Slovak Republic" or "Slovenia" or "Sweden" or "Sint Maarten" or "Seychelles" or "Turks and Caicos Islands" or "Trinidad and Tobago" or "Trinidad" or "Tobago" or "Taiwan" or "Uruguay" or "United States" or "British Virgin Islands" or "Virgin Islands")) |
| PubMed | (Population Characteristics[MeSH Terms] or "Population"[MeSH Terms] or "Demography"[MeSH Terms] or "Demograph*"[TiAb] or "Family Planning Services"[MeSH Terms] or "Family Plan*"[TiAb] OR "Reproduction"[MeSH Terms] or "Reproductive Techniques"[MeSH Terms] or "Contraception"[MeSH Terms] or "Contraceptive Agents"[MeSH Terms] or "Contracept*"[TiAb] or "Birth control"[TiAb] OR "Socioeconomic Factors"[MeSH Terms] or "Socioeconomic"[TiAb] or "Income"[TiAb] or "Financ*"[TiAb] or "Education"[MeSH Terms] or "Education*"[TiAb] or "Academic"[TiAb] OR "Psychiatry"[MeSH Terms] or "Psychology"[MeSH Terms] or "Behavior"[MeSH Terms] or "Behavioral Sciences"[MeSH Terms] or "Sociology"[MeSH Terms] or "Social Sciences"[MeSH Terms] OR "Marital Status"[MeSH Terms] or "Marital status"[TiAb] or "Married"[TiAb] or "Maternal Age"[MeSH Terms] or "Paternal Age"[MeSH Terms] or "Maternal age"[TiAb] or "Mother's age"[TiAb] or "Paternal age"[TiAb] or "Father's age"[TiAb] or "Parental age"[TiAb] OR "Religion"[MeSH Terms] or "Religion"[TiAb] or "Lifestyle"[TiAb] OR "Delivery of Health Care"[MeSH Terms] or "Health"[MeSH Terms] OR "Comorbidity"[MeSH Terms] or "Morbidity"[MeSH Terms] or "Comorbid*"[TiAb] or "Comorbid*"[TiAb] or "Co-morbid*"[TiAb] or "Morbid*"[TiAb] OR "Determinant"[TiAb] or "Factor*"[TiAb] or "Influenc*"[TiAb] or "Barrier"[TiAb] or "Caus*"[TiAb] or "Predict*"[TiAb] or "Characteristic"[TiAb] or "Enabl*"[TiAb] or "Encourag*"[TiAb] or "Motiv*"[TiAb] or "Help*"[TiAb] or "Indicat*"[TiAb] or "Drive*"[TiAb] or "Decision"[TiAb]) AND ("Birth spacing"[TiAb] or "Birth to birth spacing"[TiAb] or "Birth to conception spacing"[TiAb] or "Birth to delivery spacing"[TiAb] or "Birth to pregnancy spacing"[TiAb] or "Conception spacing"[TiAb] or "Delivery spacing"[TiAb] or "Delivery to conception spacing"[TiAb] or "Delivery to delivery spacing"[TiAb] or "Interbirth spacing"[TiAb] or "Inter-birth spacing"[TiAb] or "Inter birth spacing"[TiAb] or "Interconception spacing"[TiAb] or "Inter-conception spacing"[TiAb] or "Inter conception spacing"[TiAb] or "Interdelivery spacing"[TiAb] or "Inter-delivery spacing"[TiAb] or "Inter delivery spacing"[TiAb] or "Interpregnancy spacing"[TiAb] or "Inter-pregnancy spacing"[TiAb] or "Inter pregnancy spacing"[TiAb] or "Pregnancy spacing"[TiAb] or "Time to birth spacing"[TiAb] or Birth interval[TiAb] or "Birth to birth interval"[TiAb] or "Birth to conception interval"[TiAb] or "Birth to delivery interval"[TiAb] or "Birth to pregnancy interval"[TiAb] or "Conception interval"[TiAb] or "Delivery interval"[TiAb] or "Delivery to conception interval"[TiAb] or "Delivery to delivery interval"[TiAb] or "Interbirth interval"[TiAb] or "Inter-birth interval"[TiAb] or "Inter birth interval"[TiAb] or "Interconception interval"[TiAb] or "Inter-conception interval"[TiAb] or "Inter conception interval"[TiAb] or "Interdelivery interval"[TiAb] or "Inter-delivery interval"[TiAb] or "Inter delivery interval"[TiAb] or "Interpregnancy interval"[TiAb] or "Inter-pregnancy interval"[TiAb] or "Inter pregnancy interval"[TiAb] or "Pregnancy interval"[TiAb] or "Time to birth interval"[TiAb]) AND (MESH("Developed Countries") OR TI,AB(("High income" or "High-income" or "Developed" or "Industrialised" or "Industrialized") NEAR/0 ("Countr*" or "Nation")) OR TI,AB("Aruba" or "Andorra" or "United Arab Emirates" or "Emirates" or "Antigua and Barbuda" or "Antigua" or "Barbuda" or "Australia" or "Austria" or "Belgium" or "Bahrain" or "Bahamas" or "Bermuda" or "Barbados" or "Brunei" or "Canada" or "Switzerland" or "Channel Islands" or "Chile" or "Curaçao" or "Cayman Islands" or "Cyprus" or "Czech Republic" or "Germany" or "Denmark" or "Spain" or "Estonia" or "Finland" or "France" or "Faroe Islands" or "United Kingdom" or "Britain" or "Northern Ireland" or "England" or "Scotland" or "Wales" or "Gibraltar" or "Greece" or "Greenland" or "Guam" or "Hong Kong" or "Croatia" or "Hungary" or "Isle of Man" or "Ireland" or "Iceland" or "Israel" or "Italy" or "Japan" or "St. Kitts and Nevis" or "Saint Kitts and Nevis" or "St. Kitts and Nevis" or "Saint Kitts and Nevis" or "St. Kitts" or "Nevis" or "Republic of Korea" or "South Korea" or "Kuwait" or "Liechtenstein" or "Lithuania" or "Luxembourg" or "Latvia" or "Macao" or "St. Martin" or "Saint Martin" or "Saint-Martin" or "Monaco" or "Malta" or "Northern Mariana Islands" or "New Caledonia" or "Netherlands" or "Holland" or "Norway" or "Nauru" or "New Zealand" or "Oman" or "Palau" or "Poland" or "Puerto Rico" or "Portugal" or "French Polynesia" or "Qatar" or "Saudi Arabia" or "Singapore" or "San Marino" or "Slovak Republic" or "Slovenia" or "Sweden" or "Sint Maarten" or "Seychelles" or "Turks and Caicos Islands" or "Trinidad and Tobago" or "Trinidad" or "Tobago" or "Taiwan" or "Uruguay" or "United States" or "British Virgin Islands" or "Virgin Islands")) |
| Scopus | (KEY ( ( "Population" ) ) OR TITLE-ABS-KEY ( ( "Demograph*" OR "Family Plan*" ) ) OR KEY ( ( "Reproduction" ) ) OR TITLE-ABS-KEY ( ( "Contracept*" OR "Birth control" ) ) OR TITLE-ABS-KEY ( ( "Socioeconomic" OR "Income" OR "Financ*" OR "Education*" OR "Academic" ) ) OR KEY ( ( "Psychiatry" OR "Psychology" or "Behavior" OR "Behavioral Science" OR "Social Science" OR "Sociology" ) ) OR TITLE-ABS-KEY ( ( "Marital status" OR "Married" ) ) OR ( TITLE-ABS-KEY ( ( "Maternal" OR "Mother's" OR "Paternal" OR "Father's" OR "Parental" ) W/0 ( "Age" ) ) ) OR TITLE-ABS-KEY ( ( "Religion" OR "Lifestyle" ) ) OR TITLE-ABS-KEY ( ( "Health" ) )OR TITLE-ABS-KEY ( ( "Comorbidity" OR "Co-morbidity" OR "Morbidity" ) ) OR TITLE-ABS-KEY ( ( "Determinant" OR "Factor*" OR "Influenc*" OR "Barrier" OR "Caus*" OR "Predict*" OR "Characteristic" OR "Enabl*" OR "Encourag*" OR "Motiv*" OR "Help*" OR "Indicat*" OR "Drive*" OR "Decision" ) )) AND (TITLE-ABS-KEY ( ( "Birth" OR "Birth to birth" OR "Birth to conception" OR "Birth to delivery" OR "Birth to pregnancy" OR "Conception" OR "Delivery" OR "Delivery to conception" OR "Delivery to delivery" OR "Interbirth" OR "Inter-birth" OR "Inter birth" OR "Interconception" OR "Inter-conception" OR "Inter conception" OR "Interdelivery" OR "Inter-delivery" OR "Inter delivery" OR "Interpregnancy" OR "Inter-pregnancy" OR "Inter pregnancy" OR "Pregnancy" OR "Time to birth" ) w/0 ("Spacing" OR "Interval") ) OR TITLE-ABS-KEY ( ( "Interval* between birth*" or "Repeat pregnanc*" ) )) AND (TITLE-ABS-KEY ( ( "High income" OR "High-income" OR "Developed" OR "Industrialised" OR "Industrialized" ) W/0 ( "Countr*" OR "Nation" ) ) OR TITLE-ABS-KEY ( ( "Aruba" OR "Andorra" OR "United Arab Emirates" OR "Emirates" OR "Antigua and Barbuda" OR "Antigua" OR "Barbuda" OR "Australia" OR "Austria" OR "Belgium" OR "Bahrain" OR "Bahamas" OR "Bermuda" OR "Barbados" OR "Brunei" OR "Canada" OR "Switzerland" OR "Channel Islands" OR "Chile" OR "Curaçao" OR "Cayman Islands" OR "Cyprus" OR "Czech Republic" OR "Germany" OR "Denmark" OR "Spain" OR "Estonia" OR "Finland" OR "France" OR "Faroe Islands" OR "United Kingdom" OR "Britain" OR "Northern Ireland" OR "England" OR "Scotland" OR "Wales" OR "Gibraltar" OR "Greece" OR "Greenland" OR "Guam" OR "Hong Kong" OR "Croatia" OR "Hungary" OR "Isle of Man" OR "Ireland" OR "Iceland" OR "Israel" OR "Italy" OR "Japan" OR "St. Kitts and Nevis" OR "Saint Kitts and Nevis" OR "St. Kitts and Nevis" OR "Saint Kitts and Nevis" OR "St. Kitts" OR "Nevis" OR "Republic of Korea" OR "South Korea" OR "Kuwait" OR "Liechtenstein" OR "Lithuania" OR "Luxembourg" OR "Latvia" OR "Macao" OR "St. Martin" OR "Saint Martin" OR "Saint-Martin" OR "Monaco" OR "Malta" OR "Northern Mariana Islands" OR "New Caledonia" OR "Netherlands" OR "Holland" OR "Norway" OR "Nauru" OR "New Zealand" OR "Oman" OR "Palau" OR "Poland" OR "Puerto Rico" OR "Portugal" OR "French Polynesia" OR "Qatar" OR "Saudi Arabia" OR "Singapore" OR "San Marino" OR "Slovak Republic" OR "Slovenia" OR "Sweden" OR "Sint Maarten" OR "Seychelles" OR "Turks and Caicos Islands" OR "Trinidad and Tobago" OR "Trinidad" OR "Tobago" OR "Taiwan" OR "Uruguay" OR "United States" OR "British Virgin Islands" OR "Virgin Islands" ) )) |
| Web of Science | (TS=(("Birth" or "Birth to birth" or "Birth to conception" or "Birth to delivery" or "Birth to pregnancy" or "Conception" or "Delivery" or "Delivery to conception" or "Delivery to delivery" or "Interbirth" or "Inter-birth" or "Inter birth" or "Interconception" or "Inter-conception" or "Inter conception" or "Interdelivery" or "Inter-delivery" or "Inter delivery" or "Interpregnancy" or "Inter-pregnancy" or "Inter pregnancy" or "Pregnancy" or "Time to birth") NEAR/0 ("Spacing" or "Interval"))) OR (TS=(("Interval? between birth?" or "Repeat pregnanc*"))) AND ((TS=(("Birth" or "Birth to birth" or "Birth to conception" or "Birth to delivery" or "Birth to pregnancy" or "Conception" or "Delivery" or "Delivery to conception" or "Delivery to delivery" or "Interbirth" or "Inter-birth" or "Inter birth" or "Interconception" or "Inter-conception" or "Inter conception" or "Interdelivery" or "Inter-delivery" or "Inter delivery" or "Interpregnancy" or "Inter-pregnancy" or "Inter pregnancy" or "Pregnancy" or "Time to birth") NEAR/0 ("Spacing" or "Interval"))) OR (TS=(("Interval? between birth?" or "Repeat pregnanc*"))) AND ((TS=(("High income" or "High-income" or "Developed" or "Industrialised" or "Industrialized") NEAR/0 ("Countr*" or "Nation"))) OR (TS=(("Aruba" or "Andorra" or "United Arab Emirates" or "Emirates" or "Antigua and Barbuda" or "Antigua" or "Barbuda" or "Australia" or "Austria" or "Belgium" or "Bahrain" or "Bahamas" or "Bermuda" or "Barbados" or "Brunei" or "Canada" or "Switzerland" or "Channel Islands" or "Chile" or "Curaçao" or "Cayman Islands" or "Cyprus" or "Czech Republic" or "Germany" or "Denmark" or "Spain" or "Estonia" or "Finland" or "France" or "Faroe Islands" or "United Kingdom" or "Britain" or "Northern Ireland" or "England" or "Scotland" or "Wales" or "Gibraltar" or "Greece" or "Greenland" or "Guam" or "Hong Kong" or "Croatia" or "Hungary" or "Isle of Man" or "Ireland" or "Iceland" or "Israel" or "Italy" or "Japan" or "St. Kitts and Nevis" or "Saint Kitts and Nevis" or "St. Kitts and Nevis" or "Saint Kitts and Nevis" or "St. Kitts" or "Nevis" or "Republic of Korea" or "South Korea" or "Kuwait" or "Liechtenstein" or "Lithuania" or "Luxembourg" or "Latvia" or "Macao" or "St. Martin" or "Saint Martin" or "Saint-Martin" or "Monaco" or "Malta" or "Northern Mariana Islands" or "New Caledonia" or "Netherlands" or "Holland" or "Norway" or "Nauru" or "New Zealand" or "Oman" or "Palau" or "Poland" or "Puerto Rico" or "Portugal" or "French Polynesia" or "Qatar" or "Saudi Arabia" or "Singapore" or "San Marino" or "Slovak Republic" or "Slovenia" or "Sweden" or "Sint Maarten" or "Seychelles" or "Turks and Caicos Islands" or "Trinidad and Tobago" or "Trinidad" or "Tobago" or "Taiwan" or "Uruguay" or "United States" or "British Virgin Islands" or "Virgin Islands"))) |
| Google Scholar | (Determinant\|Factor\|Predictor\|Characteristic\|Barrier) and “Birth\|Conception\|Delivery\|Interpregnancy\|Pregnancy(Spacing)”\|“Birth\|Conception\|Delivery\|Interpregnancy\|Pregnancy(Interval)”) |

Supplementary table 3. Records excluded at full-text screening with reasons (n=170)

| **Item number** | **Study reference** | **Justification for exclusion** |
| --- | --- | --- |
| 1 | Houle & Vladimir. Regularities and peculiarities of birth schedules in industrialized countries: an analysis of FFS data. 2006 | Not a peer-reviewed journals. Working paper. |
| 2 | Lin. Ideal and actual intervals to first birth in Singapore. Asian Population Studies. 2022;18(1): 41-60 | Outcome was not pregnancy spacing. |
| 3 | Rodriguez et al. Association of Expanded Prenatal Care Coverage for Immigrant Women with Postpartum Contraception and Short Interpregnancy Interval Births. JAMA Network Open. 2021; 4(8):e2118912 | Did not report a suitable effect of association. |
| 4 | Luca et al. Evaluating teen options for preventing pregnancy: Impacts and mechanisms. 21. Journal of Health. 2021; 77:102459 | Did not report a suitable effect of association. |
| 5 | Jones et al. Fertility intentions and recent births among US abortion patients. Contraception. 2021; 103(2):75-79 | Did not report a suitable effect of association. |
| 6 | Reardon & Craver. Intervals and outcomes of first and second pregnancies in low-income women: A record- linkage longitudinal prospective cohort study. Medical Science Monitor. 2021; 25(27):e931596 | Did not report a suitable effect of association. |
| 7 | Sait et al. Knowledge, attitudes, and perception patterns of contraception methods: Cross-sectional study among Saudi males. Urology Annals. 2021; 13(3): 243–253 | Did not report a suitable effect of association. |
| 8 | Steenland et al. Medicaid payments for immediate postpartum long-acting reversible contraception: Evidence from South Carolina. Health Affairs. 2021; [40(2): 334–342.](https://www.ncbi.nlm.nih.gov/entrez/eutils/elink.fcgi?dbfrom=pubmed&retmode=ref&cmd=prlinks&id=33523747) | Did not report a suitable effect of association. |
| 9 | Willis et al. Post-partum interval and time to pregnancy in a prospective preconception cohort. Paediatric & Perinatal Epidemiology. 2021;35(3):271-280 | Pregnancy spacing was the exposure. |
| 10 | Rouse et al. Pregnancy and parenting among youth transitioning from foster care: A mixed methods study. Child & Youth Care Forum. 2021;50:167-197 | Outcome was not pregnancy spacing. |
| 11 | Luttges et al. Pregnant again? Perspectives of adolescent and young mothers who and do not experience a repeat pregnancy in adolescence. International Journal of Qualitative Studies on Health and Well-Being. 2021;16(1): 1898317. | Qualitative study |
| 12 | Tung et al. Risk of child maltreatment in Chinese teenage and young mothers with rapid repeat pregnancy: The moderating role of family cohesion and support from friends. Journal of Interpersonal Violence. 2021;36(23-24):NP13564-NP13581 | Not a high-income country. |
| 13 | Jones et al. Sex and female empowerment (SAFE): A randomized trial comparing sexual health interventions for women in treatment for opioid use disorder. Drug Alcohol Dependence. 2021;1(221);108634. | Outcome was not pregnancy spacing. |
| 14 | Ramage et al. Trends in adolescent rapid repeat pregnancy in Canada. Journal of Obstetrics and Gynaecology Canada. 2021;43(5):589-595 | Pregnancy spacing was the exposure. |
| 15 | Haldre et al. Trends in teenage delivery and abortion rates in Estonia over more than two decades: a nationwide register-based study. European Journal of Public Health. 2021;31(4):790-796 | Did not report a suitable effect of association. |
| 16 | Preis et al. Fertility intentions and the way they change following birth- a prospective longitudinal study. BMC pregnancy and childbirth. 2020;20:228 | Outcome was not pregnancy spacing. |
| 17 | Rendall et al. Intentionally or ambivalently risking a short interpregnancy interval: Reproductive-readiness factors in women’s postpartum non-use of contraception. Demography. 2020;57(3):821-841 | Outcome was not pregnancy spacing. |
| 18 | Šťastná et al. Parental leave policies and second births: A comparison of Czechia and Slovakia. Population Research and Policy Review. 2020;39:415-437 | Did not report a suitable effect of association. |
| 19 | Mönkediek. Patterns of spatial proximity and the timing and spacing of bearing children. Demographic Research. 2020:42(16):461-469. | Did not report a suitable effect of association. |
| 20 | Harrison et al. Postpartum contraceptive use among Denver-based adolescents and young adults: Association with subsequent repeat delivery. Journal of Pediatric and Adolescent Gynecology.2020;[33(4): 393–397.e1.](https://www.ncbi.nlm.nih.gov/entrez/eutils/elink.fcgi?dbfrom=pubmed&retmode=ref&cmd=prlinks&id=32251837) | Did not report a suitable effect of association. |
| 21 | Miranda. Recent trends in birth intervals in Sweden: A decline of the speed-premium effect? European Journal of Population. 2020;36(3):499-510 | Did not report a suitable effect of association. |
| 22 | Kim et al. The relationship between the ethnic composition of neighbourhood and fertility behaviours among immigrant wives in Taiwan. Asian Population Studies. 2020;16(2):201-219 | Did not report a suitable effect of association. |
| 23 | Gallagher et al. Antenatal contraception counselling and provision of contraception after delivery for first-time young mothers enrolled with a Family Nurse Partnership programme. BMJ Sexual & Reproductive Health. 2019; 45:243-248. | Outcome was not pregnancy spacing. |
| 24 | Aliaga et al. Association between affective disorders presenting before and during pregnancy and pre-term birth, considering socio-demographic factors, obstetric factors, health conditions, and use of medication. Aten Primaria. 2019;51(10):626-636 | Outcome was not pregnancy spacing. |
| 25 | Steenland et al. Association between South Carolina Medicaid's change in payment for immediate postpartum long-acting reversible contraception and birth intervals. JAMA. 2019;322(1):76-78 | Did not report a suitable effect of association. |
| 26 | Fuse. Gender and parenting difficulty of the first-born: Implications for parity progression among Japanese couples. Journal of Comparative Family Studies. 2019;50(2):116-138. | Outcome was not pregnancy spacing. |
| 27 | Upadhyay et al. Intended pregnancy after receiving vs. being denied a wanted abortion. Contraception. 2019: 99(1):42-47 | Outcome was not pregnancy spacing. |
| 28 | Yang et al. Interpregnancy intervals and women’s knowledge of the ideal timing between birth and conception. BMJ Sexual & Reproductive Health. 2019:45:249-254. | Did not report a suitable effect of association. |
| 29 | Lin et al. Long-term outcomes for teen mothers who participated in a mentoring program to prevent repeat teen pregnancy. Journal of the National Medical Association. 2019;111(3):296-301 | Did not report a suitable effect of association. |
| 30 | Frarey et al. Postpartum contraceptive counseling for first-time adolescent mothers: a randomized controlled trial. Archives of Gynecology and Obstetrics. 2019;299(2):361-369 | Did not report a suitable effect of association. |
| 31 | Ziuddeen et al. The duration of the interpregnancy interval in multiparous women and maternal weight gain between pregnancies: findings from a UK population-based cohort. Scientific Reports. 2019;9:9175 | Pregnancy spacing was the exposure. |
| 32 | Hubinont et al. A clinico-pathologic study of placenta percreta. International Journal of Gynaecology and Obstetric. 2018;140(3):365-369. | Outcome was not pregnancy spacing. |
| 33 | Haider et al. Adolescent contraception use after pregnancy, an opportunity for improvement. Journal of Pediatric and Adolescent Gynecology. 2018;31(4):388-393. | Outcome was not pregnancy spacing. |
| 34 | Alsaleem et al. Contraceptive use as limiters and spacers among women of reproductive age in southwestern, Saudi Arabia. Saudi Medical Journal. 2018;39(11):1109-1115. | Did not report a suitable effect of association. |
| 35 | Srinivasan et al. Delivering interconception care during well-child visits: An IMPLICIT network study. Journal of the American Board of Family Medicine. 2018;31(2):201-210 | Outcome was not pregnancy spacing. |
| 36 | Hubber et al. Factors associated with pregnancy intention among women who have experienced a short birth interval: findings from the 2009 to 2011 Mississippi and 2009 Tennessee Pregnancy Risk Assessment Monitoring System. Annals of Epidemiology. 2018;28(6):372-376 | Outcome was not pregnancy spacing. |
| 37 | Martinez et al. Fertility of Men and Women Aged 15–44 in the United States: National Survey of Family Growth, 2011–2015. National Health Statistics Reports. 2018;113:1-17 | Did not report a suitable effect of association. |
| 38 | Rojas et al. First and second births among immigrants and their descendants in Switzerland. Demographic Research. 2018;38(11):247-286 | Did not report a suitable effect of association. |
| 39 | Maralani et al. Intensive parenting: Fertility and breastfeeding duration in the United States. Demography. 2018;55(5):1681-1704 | Did not report a suitable effect of association. |
| 40 | Coo et al. Interpregnancy Intervals in a Contemporary Manitoba Cohort: Prevalence of So-Called Suboptimal Intervals and Associated Maternal Characteristics. Journal of obstetrics and gynaecology Canada. 2018;40(8):1024-1030 | Did not report a suitable effect of association. |
| 41 | Okoroh et al. Policy change is not enough: Engaging provider champions on immediate postpartum contraception. American Journal of Obstetrics and Gynecology. 2018; 218(6): 590.e1–590.e7. | Outcome was not pregnancy spacing. |
| 42 | Loree et al. Postpartum contraceptive use and rapid repeat pregnancy among women who use substances. Substance Use and Misuse. 2018;53(1):162-169 | Did not report a suitable effect of association. |
| 43 | Auger et al. Quantile regression analysis of language and interpregnancy interval in Quebec, Canada. Health Promotion and Chronic Disease Prevention in Canada: Research, Policy and Practice. 2018;38(5): 200–209 | Did not report a suitable effect of association. |
| 44 | Korjamo et al. Risk factors and the choice of long-acting reversible contraception following medical abortion: effect on subsequent induced abortion and unwanted pregnancy. European Journal of Contraception and Reproductive Health Care. 2018;23(2):89-96 | Outcome was not pregnancy spacing. |
| 45 | Conzuelo-Rodriguez & Naimi. The Impact of computing interpregnancy intervals without accounting for intervening pregnancy events. Paediatric and Perinatal Epidemiology. 2018;32(2):141-148 | Was not focused on determinants of pregnancy spacing. |
| 46 | Ahrens et al. Unintended pregnancy and interpregnancy interval by maternal age, National Survey of Family Growth. Contraception. 2018;[98(1): 52–55.](https://www.ncbi.nlm.nih.gov/entrez/eutils/elink.fcgi?dbfrom=pubmed&retmode=ref&cmd=prlinks&id=29501647) | Did not report a suitable effect of association. |
| 47 | Stevens et al. A randomized trial of motivational interviewing and facilitated contraceptive access to prevent rapid repeat pregnancy among adolescent mothers. American Journal of Obstetrics and Gynecology. 2017;217(4):423-423 | Did not report a suitable effect of association. |
| 48 | Gough. Birth spacing, human capital, and the motherhood penalty at midlife in the United States. Demographic Research. 2017;37(13):363-416. | Did not report a suitable effect of association. |
| 49 | Fortier & Foster. It was kind of like if it happens it happens. It wasn't planned, it wasn't intentional: Young mothers' experiences with subsequent pregnancy in Ottawa, Canada. FACETS. 2017;2(2) | Qualitative study |
| 50 | Van Gruting et al. Macroscopic and microscopic morphology of first trimester miscarriage and subsequent pregnancy outcome - An exploratory study. Placenta. 2017;53:16-22 | Did not report a suitable effect of association. |
| 51 | Kreyenfeld et al. Social policies, separation, and second birth spacing in Western Europe. Demographic Research. 2017;37(37):1245-1274 | Did not report a suitable effect of association. |
| 52 | Dee et al. Trends in repeat births and use of postpartum contraception among teens - United States, 2004-2015. Morbidity and mortality weekly report. 2107;28;66(16):422-426 | Did not report a suitable effect of association. |
| 53 | Raymond et al. Effects of depot medroxyprogesterone acetate injection timing on medical abortion efficacy and repeat pregnancy: A randomized controlled trial. Obstetrics and Gynecology. 2017;128(4):739-45 | Did not report a suitable effect of association. |
| 54 | Sakharkar et al. Impact of the 'Providing Access to Continued Education' programme on repeat teen pregnancy in The Bahamas. West Indian Medical Journal. 2016;65(2):332-336 | Did not report a suitable effect of association. |
| 55 | Holmlund et al. Induced abortion - impact on a subsequent pregnancy in first-time mothers: A registry-based study. BMC Pregnancy and Childbirth. 2016;16(1):325 | Pregnancy spacing was the exposure. |
| 56 | Heller et al. Postpartum contraception: a missed opportunity to prevent unintended pregnancy and short inter-pregnancy intervals. The Journal of Family Planning and Reproductive Health Care. 2016;42:93-98. | Did not report a suitable effect of association. |
| 57 | Ng & Steer. Prediction of an estimated delivery date should take into account both the length of a previous pregnancy and the interpregnancy interval. European Journal of Obstetrics and Gynecology and Reproductive Biology. 2016;201:101-107 | Pregnancy spacing was the exposure. |
| 58 | Thoma et al. Short interpregnancy intervals in 2014: Differences by maternal demographic characteristics. NCHS data brief. 2016 | Did not report a suitable effect of association. Data brief. |
| 59 | Bhandari et al. Superfertility is more prevalent in obese women with recurrent early pregnancy miscarriage. BJOG: An International Journal of Obstetrics and Gynaecology. 2016;123(2):217-22 | Did not report a suitable effect of association. |
| 60 | Lien & Wang. The timing of childbearing: The role of human capital and personal preferences. Journal of Macroeconomics. 2016; 49:247-264. | Did not report a suitable effect of association. Economics study. |
| 61 | Cohen et al. Twelve-month contraceptive continuation and repeat pregnancy among young mothers choosing postdelivery contraceptive implants or postplacental intrauterine devices. Contraception. 2016;98(2):178-183 | Did not report a suitable effect of association. |
| 62 | Anderson & Pierce. Depressive symptoms and violence exposure: Contributors to repeat pregnancies among adolescents. Journal of Perinatal Education. 2015;24(4): 225–238 | Outcome was not pregnancy spacing. |
| 63 | Copen et al. Interpregnancy intervals in the United States: Data from the birth certificate and the National Survey of Family Growth. National Vital Statistics Reports. 2015;64(4):1-11 | Did not report a suitable effect of association. |
| 64 | Elvander et al. Mode of delivery and the probability of subsequent childbearing: a population-based register study. BJOG : an international journal of obstetrics and gynaecology. 2015;122(12):1593-600 | Did not report a suitable effect of association. |
| 65 | Kim & Song. Neighborhood effects of ethnic composition on fertility among foreign wives in South Korea. Development and Society. 2015;44(3)389-410 | Did not report a suitable effect of association. |
| 66 | Rotering & Bras. With the Help of Kin?: Household Composition and Reproduction in The Netherlands, 1842–1920. Human Nature. 2015;26(1):102-121 | Did not report a suitable effect of association. |
| 67 | Tran et al. Change in smoking status during two consecutive pregnancies: A population-based cohort study. BJOG : an international journal of obstetrics and gynaecology. 2014;121(13):1611-1620 | Pregnancy spacing was the exposure. |
| 68 | Berg & Rotkirch. Faster Transition to the Second Child in late 20th Century Finland: A Study of Birth Intervals. Finnish Yearbook of Population Research. 2014;49:73-86 | Did not report a suitable effect of association. |
| 69 | Denzler et al. Latency after preterm prelabor rupture of the membranes: Increased risk for periventricular leukomalacia. Journal of Pregnancy. 2014;1;874894 | Outcome was not pregnancy spacing. |
| 70 | Gyorffy et al. Reproductive health and burn-out among female physicians: Nationwide, representative study from Hungary. BMC Womens Health. 2014;2(14):121 | Did not report a suitable effect of association. |
| 71 | Nenko & Jasienska. First birth interval, an indicator of energetic status, is a predictor of lifetime reproductive strategy. American Journal of Human Biology. 2013;25(1):78-82 | Pregnancy spacing was the exposure. |
| 72 | Scribano et al. The effects of intimate partner violence before, during, and after pregnancy in nurse visited first time mothers. Maternal and Child Health. 2013;17(2):307-318 | Did not report a suitable effect of association. |
| 73 | Cox et al. Evaluation of raising adolescent families together program: A medical home for adolescent mothers and their children. American Journal of Public Health. 2012;102(10):1879-1885 | Did not report a suitable effect of association. |
| 74 | Bohnert et al. Offspring sex preference in frontier America. Journal of Interdisciplinary History. 2012;42(2):519-541 | Did not report a suitable effect of association. |
| 75 | Teitler et al. Prenatal care and subsequent birth intervals. Perspectives on Sexual and Reproductive Health. 2012;44(1):13-21 | Did not report a suitable effect of association. |
| 76 | Carvajal et al. Repeat pregnancy prevention self-efficacy in adolescents: Associations with provider communication, provider type, and depression. Southern Medical Journal. 2012;105(11):591-597 | Outcome was not pregnancy spacing. |
| 77 | Palen et al. The use of reconsent in a national evaluation of adolescent reproductive health programs. Journal of Adolescent Health. 2012;51(2):184-189 | Did not report a suitable effect of association. |
| 78 | Kolk. Deliberate birth spacing in nineteenth century Northern Sweden. European Journal of Population. 2011;27:337-359 | Pregnancy spacing was the exposure. |
| 79 | Salihu et al. Effectiveness of a federal healthy start program in reducing primary and repeat teen pregnancies: Our experience over the decade. Journal of Pediatric and Adolescent Gynecology. 2011;24(3):153-160 | Did not report a suitable effect of association. |
| 80 | Holland & Thomson. Stepfamily childbearing in Sweden: Quantum and tempo effects, 1959-99. Population Studies. 2011;65(1):115-128. | Outcome was not pregnancy spacing. |
| 81 | Johow et al. The presence of a paternal grandmother lengthens interbirth interval following the birth of a granddaughter in Krummhorn (18th and 19th centuries). Evolution and Human Behaviour. 2011;32(5):315-325 | Did not report a suitable effect of association. |
| 82 | Robson & Smith. Twinning in humans: Maternal heterogeneity in reproduction and survival. Proceedings of the Royal Society B: Biological Sciences. 2011;278(1725):3755-3761. | Outcome was not pregnancy spacing. |
| 83 | Schreiber et al. A randomized controlled trial of the effect of advanced supply of emergency contraception in postpartum teens: a feasibility study. 2010;81(5):435-440 | Did not report a suitable effect of association. |
| 84 | Olds et al. Enduring effects of prenatal and infancy home visiting by nurses on maternal life course and government spending: Follow-up of a randomized trial among children at age 12 years. Archives of Pediatrics and Adolescent Medicine. 2010;164(5):419-424 | Outcome was not pregnancy spacing. |
| 85 | Kalberer et al. Birth records from Swiss married couples analyzed over the past 35 years reveal an aging of first-time mothers by 5.1 years while the interpregnancy interval has shortened. Fertility and Sterility. 2009;92(6):2072-2073. | Did not report a suitable effect of association. |
| 86 | Nabukera et al. Racial disparities in perinatal outcomes and pregnancy spacing among women delaying initiation of childbearing. Maternal and Child Health. 2009;13(1):81-89 | Did not report a suitable effect of association. |
| 87 | Madden & Westhoff. Rates of follow-up and repeat pregnancy in the 12 months after first-trimester induced abortion. Obstetrics and Gynecology. 2009;113(3):663-668 | Did not report a suitable effect of association. |
| 88 | Mohsin & Jalaludin. Influence of previous pregnancy outcomes and continued smoking on subsequent pregnancy outcomes: an exploratory study in Australia. BJOG : an international journal of obstetrics and gynaecology. 2008;115(11):1428-1435 | Outcome was not pregnancy spacing. |
| 89 | Eijsink et al. Pregnancy after caesarean section: Fewer or later?. Human Reproduction. 2008;23(3):543-547 | Did not report a suitable effect of association. |
| 90 | Pesonen et al. Reproductive traits following a parent-child separation trauma during childhood: a natural experiment during World War II. American Journal of Human Biology. 2008;20(3):345-351 | Outcome was not pregnancy spacing. |
| 91 | Huang. The personal tax exemption and married women's birth spacing in the United States. Public Finance Review. 2008;36(6):728-747 | Did not report a suitable effect of association. |
| 92 | El-Gihany & Hammad. Utilization of postnatal care in Al-Hassa, Saudi Arabia. Middle East Journal of Family Medicine. 2008;6(9):23-27 | Did not report a suitable effect of association. |
| 93 | Rasheed & Al-Daba. Birth interval: Perceptions and practices among urban-based Saudi Arabian women. Eastern Mediterranean Health Journal. 2007;13(4):881-892 | Did not report a suitable effect of association. |
| 94 | Zavascki et al. Predictors of repeat pregnancy among HIV-1-infected women: New epidemiology, old challenges. Journal of Acquired Immune Deficiency Syndromes. 2007;45(3):368-369 | Outcome was not pregnancy spacing. |
| 95 | Thurman et al. Preventing repeat teen pregnancy: Postpartum depot medroxyprogesterone acetate, oral contraceptive pills, or the patch? Journal of Pediatric and Adolescent Gynecology. 2007;20(2):61-65 | Did not report a suitable effect of association. |
| 96 | Kemkes. Does the sex of firstborn children influence subsequent fertility behavior? Evidence from family reconstitution. Journal of Family History. 2006;31(2):144-162 | Did not report a suitable effect of association. |
| 97 | Gray et al. Having the best intentions is necessary but not sufficient: What would increase the efficacy of home visiting for preventing second teen pregnancies? Prevention Science. 2006;7(4):389-395 | Did not report a suitable effect of association. |
| 98 | Tymicki. The Interplay between infant mortality and subsequent reproductive behaviour. Evidence for the replacement effect from historical population of Bejsce Parish, 18th-20th Centuries, Poland. 2005;30(3):240-264 | Did not report a suitable effect of association. |
| 99 | Tsay & Chu. The pattern of birth spacing during Taiwan's demographic transition. Journal of Population Economics. 2005;18(2):323-336 | Did not report a suitable effect of association. |
| 100 | Van Bavel & Kok. The role of religion in the Dutch fertility transition: Starting, spacing, and stopping in the heart of the Netherlands, 1845-1945. Continuity and Change. 2005;20(2):247-263 | Did not report a suitable effect of association. |
| 101 | Van Bavel & Kok. Birth spacing in the Netherlands. The effects of family composition, occupation and religion on birth intervals, 1820-1885. European Journal of Population. 2004;20(2):119-140 | Did not report a suitable effect of association. |
| 102 | Andersson. Childbearing developments in Denmark, Norway, and Sweden from the 1970s to the 1990s: A comparison. Demographic Research. 2004:7(3):155-176 | Outcome was not pregnancy spacing. |
| 103 | Van Bavel. Deliberate birth spacing before the fertility transition in Europe: evidence from nineteenth-century Belgium. Population Studies. 2004;58(1):95-107 | Did not report a suitable effect of association. |
| 104 | Van Bavel. Diffusion effects in the European fertility transition: Historical evidence from within a Belgian town (1846-1910). European Journal of Population. 2004;20(1):63-85 | Outcome was not pregnancy spacing. |
| 105 | Van Bavel. Birth spacing as a family strategy: Evidence from 19th century Leuven, Belgium. The History of the Family. 2003;8(4):585-604 | Did not report a suitable effect of association. |
| 106 | Al-Riyami & Afifi. Determinants of women's fertility in Oman. Saudi Medical Journal. 2003;24(7)748-753 | Outcome was not pregnancy spacing. |
| 107 | Shlay et al. Initiating contraception in sexually transmitted disease clinic setting: A randomized trial. American Journal of Obstetrics and Gynecology.2003;189(2):473-781 | Outcome was not pregnancy spacing. |
| 108 | Koniak-Griffin et al. Nurse visitation for adolescent mothers: two-year infant health and maternal outcomes. Nursing Research. 2003;52(2):127-136 | Did not report a suitable effect of association. |
| 109 | Philliber et al. Outcomes of teen parenting programs in New Mexico. Adolescence. 2003;38(151):535-553 | Did not report a suitable effect of association. |
| 110 | Kershaw et al. Short and long-term impact of adolescent pregnancy on postpartum contraceptive use: Implications for prevention of repeat pregnancy. Journal of Adolescent Health. 2003;33(5):359-368 | Outcome was not pregnancy spacing. |
| 111 | Al-Almaie. The pattern and factors associated with child spacing in eastern Saudi Arabia. The Journal of the Royal Society for the Promotion of Health. 2003;123(4):217-221 | Did not report a suitable effect of association. |
| 112 | Al-Riymani & Afif. Women empowerment and marital fertility in Oman. The Journal of the Egyptian Public Health Association. 2003;78(1-2):55-72 | Outcome was not pregnancy spacing. |
| 113 | Greksa. Population growth and fertility patterns in an Old Order Amish settlement. Annals of Human Biology. 2002;29(2):192-201 | Outcome was not pregnancy spacing. |
| 114 | Koniak-Griffin et al. Public health nursing care for adolescent mothers: Impact on infant health and selected maternal outcomes at 1 year postbirth. Journal of Adolescent Health. 2002;30(1):44-54 | Did not report a suitable effect of association. |
| 115 | Yip & Lee. The impact of the changing marital structure on fertility of Hong Kong SAR (Special Administrative Region). Social Science & Medicine. 2002;55(12):2159-2169 | Outcome was not pregnancy spacing. |
| 116 | Schellekens & Eisenbach. The predecline rise in Israeli moslem fertility. Economic Development and Cultural Change. 2002;50(3) | Did not report a suitable effect of association. |
| 117 | Doyle et al. Inter-pregnancy folate and iron status of women in an inner-city population. British Journal of Nutrition. 2001;86(1):81-87 | Outcome was not pregnancy spacing. |
| 118 | Ahn & Mira. Job bust, baby bust?: Evidence from Spain. Journal of Population Economics. 2001;14(3):505-521 | Did not report a suitable effect of association. |
| 119 | Skomsvoll et al. Number of births, interpregnancy interval, and subsequent pregnancy rate after a diagnosis of inflammatory rheumatic disease in Norwegian women. Journal of Rheumatology. 2001;28(1):2310-2314 | Did not report a suitable effect of association. |
| 120 | Smits et al. Season of birth and reproductive performance: an analysis of family reconstitutions of 800 women born in The Netherlands at the end of the 19th century. Chronobiology International. 2001;18(3):525-539 | Did not report a suitable effect of association. |
| 121 | Clegg. Starting, spacing and stopping in the reproductive histories of outer Hebridean families. Journal of Biosocial Science. 2001;33(3):405-426 | Did not report a suitable effect of association. |
| 122 | Koziel & Ulijaszek. Waiting for Trivers and Willard: Do the rich really favor sons? American Journal of Physical Anthropology. 2001;115(1):71-79 | Did not report a suitable effect of association. |
| 123 | Khattab. Cross-sectional study of a child health care programme at one family practice centre in Saudi Arabia. Eastern Mediterranean Health Journal. 2000;6(2-3):246-259 | Outcome was not pregnancy spacing. |
| 124 | Polo & Fuster. Determinants of birth interval in a rural Mediterranean population (La Alpujarra, Spain). Human Biology. 2000;72(5):877-890 | Did not report a suitable effect of association. |
| 125 | Symeonidou. Expected and actual family size in Greece: 1983-1997. European Journal of Population. 2000;16(4):335-352 | Outcome was not pregnancy spacing. |
| 126 | Smits et al. Fecundity of daughters born after short, intermediate, or long birth intervals. An analysis of family reconstitutions from The Netherlands, late 19th-early 20th century. Social Biology. 2000;47(1-2):18-33 | Did not report a suitable effect of association. |
| 127 | Korpelainen. Fitness, reproduction and longevity among European aristocratic and rural Finnish families in the 1700s and 1800s. Proceedings of the Royal Society. 2000;267(1454):1765-1770 | Did not report a suitable effect of association. |
| 128 | Lynn. New evidence for dysgenic fertility for intelligence in the United States. Social Biology. 1999;46(1-2):146-153 | Outcome was not pregnancy spacing. |
| 129 | Jacoby et al. Rapid repeat pregnancy and experiences of interpersonal violence among low-income adolescents. American Journal of Preventive Medicine. 1999;16(4):318-321 | Did not report a suitable effect of association. |
| 130 | Morabia & Costanza. International variability in ages at menarche, first livebirth, and menopause. World Health Organization Collaborative Study of Neoplasia and Steroid Contraceptives. American Journal of Epidemiology. 1998;148(12):1195-1205 | Outcome was not pregnancy spacing. |
| 131 | Field et al. Polydrug-using adolescent mothers and their infants receiving early intervention. Adolescence. 1998;33(129):117-143 | Outcome was not pregnancy spacing. |
| 132 | Zavattaro & Vercauteren. International migration and biodemographical behaviour: a study of Italians in Belgium. Journal of Biosocial Science. 1997;29(3):345-354 | Did not report a suitable effect of association. |
| 133 | Blanchard & Bogaert. The relation of closed birth intervals to the sex of the preceding child and the sexual orientation of the succeeding child. Journal of Biosocial Science. 1997;29(1):111-118 | Did not report a suitable effect of association. |
| 134 | Chi & Ping-Lung. Family structure and fertility behavior in Taiwan. Population Research and Policy Review. 1996;15;327-339 | Did not report a suitable effect of association. |
| 135 | Walker. Parental benefits, employment, and fertility dynamics. Research in Population Economics. 1996;8:125-172 | Did not report a suitable effect of association. |
| 136 | Ricketts. Repeat fertility and contraceptive implant use among Medicaid recipients in Colorado. Family Planning Perspectives. 1996;28(6):278-280 | Did not report a suitable effect of association. |
| 137 | Leadbetter. School outcomes for minority-group adolescent mothers at 28 to 36 months postpartum: a longitudinal follow-up. Journal of Research on Adolescence. 1996;6(4):629-648 | Did not report a suitable effect of association. |
| 138 | Glasier. Who gives advice about postpartum contraception? Contraception. 1996;53(4):217-220 | Did not report a suitable effect of association. |
| 139 | Sangi-Highpeykar et al. Characteristics of injectable contraceptive users in a low-income population in Texas. Family Planning Perspectives. 1995;27(5):208-211 | Outcome was not pregnancy spacing. |
| 140 | Lewis et al. Drugs, poverty, pregnancy, and foster care in Los Angeles, California, 1989 to 1991. Western Journal of Medicine. 1995;163(5):435-440 | Did not report a suitable effect of association. |
| 141 | Yamaguchi & Ferguson. The stopping and spacing of childbirths and their birth-history predictors: Rational-choice theory and event-history analysis. American Sociological Review. 1995;60(2):272–298. | Did not report a suitable effect of association. |
| 142 | Stevens-Simon et al. Which teen mothers choose Norplant? Journal of Adolescence Health. 1995;16(5):350-353 | Did not report a suitable effect of association. |
| 143 | Wood et al. A multistate model of fecundability and sterility. Demography. 1994;31(3):403-426 | Outcome was not pregnancy spacing. |
| 144 | Maynard & Rangarajan. Contraceptive use and repeat pregnancies among welfare-dependent teenage mothers. Family Planning Perspectives. 1994;26(5):198-205 | Did not report a suitable effect of association. |
| 145 | Covington et al. Factors affecting number of prenatal care visits during second pregnancy among adolescents having rapid repeat births. Journal of Adolescence Health. 1994;15(7):536-542 | Outcome was not pregnancy spacing. |
| 146 | Jones & Woody. Lessons for prevention and intervention in adolescent pregnancy: a five-year comparison of outcomes of two programs for school-aged pregnant adolescents. Journal of Pediatric Health Care. 1994;8(4):152-159 | Did not report a suitable effect of association. |
| 147 | Joffe & Li. Male and female factors in fertility. American Journal of Epidemiology. 1994;140(10):921-929 | Outcome was not pregnancy spacing. |
| 148 | Kalmuss & Namerow. Subsequent childbearing among teenage mothers: The determinants of a closely spaced second birth. Family Planning Perspectives. 1994;26(4):149-159 | Did not report a suitable effect of association. |
| 149 | Lillard & Waite. A joint model of marital childbearing and marital disruption. Demography. 1993;30(4):653-681 | Did not report a suitable effect of association. |
| 150 | Seitz & Apfel. Adolescent mothers and repeated childbearing: effects of a school-based intervention program. American Journal of Orthopsychiatry. 1993;63(4):572-581. | Did not report a suitable effect of association. |
| 151 | Russo et al. Childspacing intervals and abortion among blacks and whites: A brief report. Women Health. 1993;20(3):43-51 | Did not report a suitable effect of association. |
| 152 | Rahim & Bam. Emerging patterns of child-spacing in Canada. Journal of Biosocial Science. 1993;25(2):155-167 | Did not report a suitable effect of association. |
| 153 | Bam & Rahim. Enduring effects of women's early employment experiences on child-spacing: the Canadian evidence. A Journal of Demography. 1993;47(2):303-317 | Did not report a suitable effect of association. |
| 154 | Margulis et al. Sex-biased lactational duration in a human population and its reproductive costs. Behavioral Ecology and Sociobiology. 1993;32(1):41-45 | Did not report a suitable effect of association. |
| 155 | O’Sullivan & Jacobsen. A randomized trial of a health care program for first-time adolescent mothers and their infants. Nursing Research. 1992;41(4):210-215 | Did not report a suitable effect of association. |
| 156 | Suchindran & Koo. Age at last birth and its components. Demography. 1992;29(2):227-245 | Did not report a suitable effect of association. |
| 157 | Eggebeen. Changes in sibling configurations for American preschool children. Social Biology. 1992;31(1-2):27-44. | Did not report a suitable effect of association. |
| 158 | Blossfeld & Rose. Educational expansion and changes in entry into marriage and motherhood. The experience of Italian women. Genus. 1991;48(3-4):73-91 | Did not report a suitable effect of association. |
| 159 | Dao et al. Fertility in myotonic dystrophy in Saguenay-Lac-St-Jean: a historical perspective. Clinical Genetics. 1992;42(5):234-239 | Did not report a suitable effect of association. |
| 160 | Neumark. Interpreting demographic effects in duration analyses of first birth intervals. Journal of Population Economics. 1992;5(1):17-37 | Outcome was not pregnancy spacing. |
| 161 | Linares et al. Predictors of repeat pregnancy outcome among black and Puerto Rican adolescent mothers. Journal of Development Behaviours in Pediatrics. 1992;13(2):89-94 | Did not report a suitable effect of association. |
| 162 | Boyer & Fine. Sexual abuse as a factor in adolescent pregnancy and child maltreatment. Family Planning Perspectives. 1992;24(1):4-11 | Did not report a suitable effect of association. |
| 163 | Jamieson & Buescher. The effect of family planning participation on prenatal care use and low birth weight. Family Planning Perspectives. 1992;24(5):214-218 | Did not report a suitable effect of association. |
| 164 | Adam. Convergence on the two-child family norm in Australia. Journal of Australian Population Association. 1991;8:77-91 | Did not report a suitable effect of association. |
| 165 | Finnas. Fertility in Larsmo: The effect of laestadianism. Population Studies. 1992;45(2):339-351 | Outcome was not pregnancy spacing. |
| 166 | Loh & Bam. Delayed childbearing in Canada: trends and factors. Genus. 1990;46(1-2):147-161 | Did not report a suitable effect of association. |
| 167 | Schellekens. Socio-economic determinants of marital fertility in two eighteenth-century Dutch villages. European Journal of Population. 1990;6:51-68 | Did not report a suitable effect of association. |
| 168 | Heckman & Walker. The relationship between wages and income and the timing and spacing of births: evidence from Swedish longitudinal data. Econometrica. 1990;58(6):1411-1441 | Did not report a suitable effect of association. |
| 169 | Heckman & Walker. The third birth in Sweden. Journal of Population Economics. 1990;3(4):235-275 | Did not report a suitable effect of association. |
| 170 | Boldsen & Schaumberg. Time to pregnancy—a model and its application. Journal of Biosocial Science. 1990;22(2):255-262 | Did not report a suitable effect of association. |

Supplementary table 4. Summary of the characteristics of studies reporting determinants of interpregnancy intervals in high-income countries (n=55).

| First author (year), country | Study aims | Population | Sample size | Study period | Study design | Risk factors | Outcome measure | Results | Authors conclusion |
| --- | --- | --- | --- | --- | --- | --- | --- | --- | --- |
| Abdel-Fattah (2007), Saudi Arabia | This study aimed at identifying determinants of birth spacing and attitudes toward family planning among Saudi women attending a military hospital. | Women aged 15-49 years who had been married before or were still married. | 786 women | 1^st^ February 2005 – 31^st^ January 2006 | Cross-sectional | Education level  Working status  History of chronic diseases  Husband's beliefs regarding birth spacing  Husband's occupation  Number of living children  Male to female ratio of living children  Family income | IBI | Education level (reference is able to read and write):   - Completed primary school/Intermediate level aHR 0.96 (95% CI 0.77 to 1.18) - Completed secondary school/Obtained diploma aHR 0.72 (95% CI 0.55 to 0.93) - Completed University aHR 0.67 (95% CI 0.54 to 0.96)   Working vs. unemployed/housewife:   - aHR 0.61 (95% CI 0.42 to 0.97)   Chronic disease vs. no history of chronic disease:   - aHR 0.67 (95% CI 0.52 to 0.96)   Husband beliefs regarding birth spacing (reference is disagree strongly):   - Don’t mind aHR 0.64 (95% CI 0.51 to 0.81) - Encouraging aHR 0.53 (95% CI 0.41 to 0.68) - Unknown aHR 0.68 (95% CI 0.45 to 1.03)   Husband’s occupation (reference is military officer):   - Civilian employee aHR 0.50 (95% CI 0.33 to 0.76) - Other military personnel aHR 0.56 (95% CI 0.39 to 0.81) - Professional aHR 0.68 (95% CI 0.43 to 1.07) - Technician aHR 0.69 (95% CI 0.40 to 1.19) - Retired aHR 0.37 (95% CI 0.23 to 0.58)   Number of children (reference is 1 child):   - 2-4 children aHR 0.78 (95% CI 0.60 to 1.01) - >4 children aHR 1.43 (95% CI 0.67 to 1.31)   Sex ratio of living children (reference is 1 male: 1 female):   - 1 male aHR 1.50 (95% CI 1.15 to 1.96) - >1 male aHR 1.15 (95% CI 0.93 to 1.42) - Males only aHR 1.10 (95% CI 0.82 to 1.47) - Females only aHR 1.79 (95% CI 1.28 to 2.51)   Socio-economic status (reference is more than enough):   - Enough aHR 0.87 (95% CI 0.72 to 1.04) - Not enough aHR 1.43 (95% CI 1.06 to 1.93) | Woman’s education, work status, husband’s work status, a woman’s history of chronic diseases, and husband's encouragement of interbirth spacing were the only significant predictors of longer interbirth intervals.  Shorter interbirth intervals were independently predicted by lower family income, and presence of female offspring only or equal number of male and female offspring as opposed to presence of more males |
| Albrechtsen (1998), Norway | To assess subsequent pregnancy rates and recurrence of breech, as well as interpregnancy interval after a breech presentation | Maternal record linkage of sibships, comprising the first to the fourth birth of a mother | 1,329,992 births | 1967-1994 | Cohort | Birth cohort period  Previous death of a child  Previous mode of delivery  Previous Breech presentation  Previous preterm birth | IPI | Year of birth (reference is 1967 – 1975):   - 1976-1984 after 1^st^ birth aHR 0.87 (95% CI 0.86-0.88) - 1976-1984 after 2^nd^ birth aHR 0.95 (95% CI 0.93-0.96) - 1985-1994 after 1^st^ birth aHR 0.80 (95% CI 0.80-0.81) - 1985-1994 after 2^nd^ birth aHR 1.12 (95% CI 1.11-1.13)   Previous death of a child (reference is previous child survival):   - After 1^st^ birth aHR 2.41 (95% CI 2.35 to 2.47) - After 2^nd^ birth aHR 5.99 (95% CI 5.78 to 6.21)   Previous mode of delivery (reference is vaginal delivery):   - After 1^st^ birth aHR 0.84 (95% CI 0.83 to 0.85) - After 2^nd^ birth aHR 0.86 (95% CI 0.82 to 0.84)   Previous breech delivery (reference is non-breech presentation):   - After 1^st^ birth aHR 1.00 (95% CI 0.98 to 1.01) - After 2^nd^ birth aHR 0.99 (95% CI 0.95 to 1.02)   Previous preterm delivery (reference is term birth):   - After 1^st^ birth aHR 1.15 (95% CI 1.13 to 1.16) - After 2^nd^ birth aHR 0.99 (95% CI 0.95 to 1.02) | Interpregnancy interval from the first to the second birth was the same after breech and non-breech presentation. Interpregnancy interval was mostly influenced by perinatal loss and, to a lesser extent, by gestational age, year of birth, and delivery method |
| Ali (2020), United Arab Emirates | This study aims to investigate the effect of self-reported history of previous GDM on behaviors in a future pregnancy. | Pregnant women who participated in the Mutaba’ah Study, which is an ongoing prospective mother and child cohort study. | 5,738 women | May 2017 - March 2020 | Cohort | History of gestational diabetes mellitus | Short IPI (not defined) | aOR 0.88 (00.82 to 0.94) | Women with a history of previous GDM were more likely to have had shorter interpregnancy intervals between their previous child and current pregnancy (aOR: 0.88, 95% CI 0.82–0.94, per SD increase) |
| Al-Nahedh (1999), Saudi Arabia | To examine the effects of sociodemographic factors and nutritional habits on child-spacing in a rural area of Saudi Arabia. | Women of child-bearing age who were or ever had been married in Al-Oyaynah village | 251 women | April 1995 - May 1995 | Cross-sectional | Maternal age  Parity | IBI | Maternal age (reference is 20-24 years):   - 25-29 years OR 2.9 (95% CI 1.1 to 7.8) - 30-34 years OR 7.8 (95% CI 2.7 to 22) - 35-39 years OR 19 (95% CI 5.6 to 64)   Parity (reference is primiparous or multiparous (1-3 previous births):   - Multiparous (4-5 previous births) OR 1.2 (0.5-2.7) - Multiparous (≥6 previous births OR 0.4 (0.2 to 0.95) |  |
| Al-Rumhi (2023), Oman | This study examined short interpregnancy interval, its risk factors and its association with the prevalence of adverse pregnancy outcomes among Omani pregnant women. | All women of reproductive age who had at least two  births | 597 (199 cases; 398 controls) | January to December 2020 | Case-control | Maternal age  Age at first birth  Parity  Perinatal loss | Short IPI < 24 months | - Mother’s age (aRR 1.01 95% CI 1.03 – 1.17) - Age at first birth (aRR 0.87 (95% CI 0.81 – 0.93) - Parity (aRR 0.79 95% CI 0.67 – 0.93) - Perinatal loss (aRR 0.36 95% CI 0.14 – 0.91) | Significant risk factors for a short interpregnancy interval were advanced maternal age, young age at first birth, low parity and previous perinatal loss. |
| Arora (2018), United States | The study aimed to assess fulfillment of sterilization requests while accounting for the complex interplay between insurance, clinical and social factors in a contemporary context that included both inpatient and outpatient postpartum sterilization procedures | Women delivered at an urban, tertiary-care teaching hospital or beyond with sterilization as their documented contraceptive plan | 8,654 births | 1^st^ January 2012-31^st^ December 2014 | Cohort | Insurance status (Medicaid) | Short IPI <  12 months | RR 2.57 (95% CI 1.10 to 6.0) | Women with Medicaid are more likely than women with private insurance to have a short interval repeat pregnancy after an unfulfilled sterilization request. |
| Backley (2020), United States | The objective was to evaluate the association between depressive symptoms during pregnancy and IPI among a predominantly Puerto Rican population in Western Massachusetts from 2006 to 2016. | Women of Caribbean Islanders (Puerto Rican or Dominican Republic ancestry) who had prenatal care at Baystate medical centre in Western Massachusetts. | 1,262 women of Puerto Rican or Dominican Republic ancestry | 2006-2016 | Cohort | Edinburgh Postnatal Depression Scale | Short IPI <  18 months | At least probable minor depression aHR 1.09 (95% CI 0.92 to 1.29)  Probably major depression HR 1.12 (95% CI 0.93 to 1.34) | Prenatal depressive symptoms were common in this Puerto Rican population and were associated with a modest increase in odds of short IPI. |
| Bennett (2006), United States | The purpose of this study was to assess the contribution of depressive symptoms and poor contraceptive use early in the first postpartum year to the risk of unintended repeat pregnancy at the end of that year among adults with low educational status (<12th grade or equivalence). | Sexually active, low-income, inner-city adult women (age >19) who enrolled prenatally and were followed twice after delivery. | 643 women aged ≥19 years | February 2000 - October 2002 | Cohort | Contraceptive use  Depressive symptoms ≤3 months after delivery  Low education level | BTP interval <  12 months | Contraception use (reference is highly effect methods):   - Less effective methods aOR 2.32 (95% CI 1.08 to 4.96) - No contraception aOR 2.01 (95% CI 0.93 to 4.32   Less high school vs. high school or higher:   - aOR 2.28 (95% CI 1.21 to 4.27)   Depressive symptoms vs. no symptoms 3 months after delivery:   - aOR 1.08 (95% CI 0.56 to 2.08) | Low educational status and less effective contraceptive use were associated with unintended pregnancy. Neither depressive symptoms nor contraceptive use reduced the risk of pregnancy that was associated with low educational status. |
| Brown (2018), Canada | The study evaluated the risk for rapid repeat pregnancy among women with intellectual and developmental disabilities, with whom sharing of information about pregnancy planning and contraception may be inadequate. | Women who had a live birth between Apr. 1, 2002, and Mar. 31, 2013, who were followed for 12 months to ascertain the primary outcome. | 926,222 women | 1^st^ April 2002 – 31^st^ March 2013 | Cohort | Maternal age  Continuity of primary care  Urban/rural residence  Social assistance  Parity | BTP interval < 12 months  BTP interval < 18 months  BTP interval < 24 months | Maternal age < 25 years vs. ≥25 years:   - < 12 months aRR 2.75 (95% CI 2.69 to 2.82) - < 18 months aRR 1.93 (95% CI 1.91 to 1.96) - < 24 months aRR 1.46 (95% CI 1.43 to 1.44)   No continuity of primary care vs. moderate to high continuity:   - < 12 months aRR 1.16 (95% CI 1.14 to 1.19) - < 18 months aRR 1.09 (95% CI 1.08 to 1.06) - < 24 months aRR 1.03 (95% CI 1.02 to 1.04)   Urban vs. rural residence:   - < 12 months aRR 1.35 (95% CI 1.30 to 1.40) - < 18 months aRR 1.02 (95% CI 1.00 to 1.05) - < 24 months aRR 0.91 (95% CI 0.90 to 0.93)   Social assistance vs. no social assistance:   - < 12 months aRR 1.71 (95% CI 1.65 to 1.77) - < 18 months aRR 1.46 (95% CI 1.42 to 1.50) - < 24 months aRR 1.26 (95% CI 1.23 to 1.28)   Parity/multiparous vs. primiparous:   - < 12 months aRR 1.22 (95% CI 1.19 to 1.26) - < 18 months aRR 0.85 (95% CI 0.84 to 0.86) - < 24 months aRR 0.63 (95% CI 0.62 to 0.63) | Rapid repeat pregnancy,  which was more common among  women with intellectual and developmental  disabilities, may be explained by  social, health and health care disparities. |
| Brunson (2017), United States | The study aimed to determine the initiation trends and relative effectiveness of postpartum contraceptive methods, with typical use, on prevention of short delivery intervals (27 months) among women with access to universal healthcare, including coverage that entails no copayments and allows unlimited contraceptive method switching. | TRICARE Prime enrollees who were admitted to the hospital for childbirth, abortion, ectopic pregnancy, or miscarriage. | 373,840 women | 1^st^ October 2010 – 30^th^ March 2015 | Cohort | Postpartum contraceptive method within first six months after index delivery | Short IBI (≤27 months) | Type of contraception (reference is no prescription):   - Pill, patch or ring aHR 0.8 (95% CI 0.78 to 0.81) - Depot medroxyprogesterone aHR 0.39 (95% CI 0.36 to 0.42) - Etonogestrel implant aHR 0.21 (95% CI 0.19 to 0.23) - Intrauterine device aHR 0.19 (95% CI 0.18 to 0.20) - Tubal ligation aHR 0.01 (95% CI 0.01 to 0.01) - Partner vasectomy aHR 0.05 (95% CI 0.04 to 0.06) | Postpartum initiation of long-acting reversible contraception is highly effective at the prevention of short interdelivery intervals, whereas pill, patch, or ring methods are associated with rates of short interdelivery intervals similar to users of no prescription contraception. |
| Caldwell (2022), United States | The study aimed to determine whether delivery at a Catholic hospital is associated with shorter pregnancy intervals among patients insured by Medicaid in Illinois. | Women aged 15–45 years old with an index birth | 18,627 women | January 1st 2010- 31st December 2012 | Cohort | Hospital affiliation status (Catholic, religious non-Catholic, secular) | IPI < 6 months;12 months;18 months | Catholic hospital vs. non-Catholic hospital:   - < 6 months OR 1.14 (95% CI 1.07 to 1.21) - < 12 months OR 1.18 (95% CI 1.13 to 1.24) - < 18 months OR 1.16 (95% CI 1.10 to 1.22) | Delivery at a Catholic hospital is associated with an increased risk of short-interval pregnancy. |
| Cha (2016), United States | The study examined the association between couple pregnancy intentions and rapid repeat pregnancy among women in the United States | Multiparous women with history of at least 2 completed pregnancies aged 15-19 years who cohabited with 1 husband/partner before conception of second pregnancy. | 3,463 women aged 15-19 years | 2006 to 2010 | Cohort | Maternal and paternal pregnancy intentions | RRP < 24 months | Reference is M+P+ (both maternal and paternal pregnancy intended)   - M- P- (both pregnancy unintented) aOR 1.85 (95% CI 0.82 to 4.18) - M+P- (maternal pregnancy intended, paternal pregnancy unintended) aOR 0.77 (95% CI 0.70 to 0.85) - M-P+ (maternal pregnancy unintended, paternal pregnancy intended) aOR 2.51 (95% CI 1.45 to 4.35) | Study results suggest that RRP is strongly influenced by paternal rather than maternal pregnancy intentions. |
| Cheslack-Postova (2015), United States | The study aims were to determine which characteristics of women and their previous pregnancies are associated with the odds of short or long interpregnancy intervals, and to assess whether characteristics associated with the risk of short or long intervals differ by pregnancy intention. | Women aged 15-44 years with an index birth. | 10,326 pregnancies | 1995, 2002, and 2006-2010 | Cohort | Maternal age  Education level  Marital status  Place of birth  Previous caesarean section delivery  Income (% of poverty level) | Short IPI (<12 months) | Maternal age (reference is 20-29 years):   - < 20 years aOR 0.83 (95% CI 0.66 to 1.04) - 30-34 years aOR 1.26 (95% CI 0.99 to 1.60) - ≥ 35 years aOR 1.32 (95% CI 0.86 t0 2.02)   Education level (references is less than high school):   - high school aOR 0.69 (95% CI 0.56 to 0.86) - some college aOR 0.65 (95% CI 0.51 to 0.82) - college degree aOR 0.58 (95% CI 0.43 to 0.80)   Marital status (reference is married):   - Never married aOR 1.19 (95% CI 0.96 to 1.48) - Formerly married aOR 1.15 (95% CI 0.81 to 1.64)   Born outside of the US vs. born in United States:   - aOR 0.69 (95% CI 0.51 to 0.93)   Caesarean section delivery vs. no caesarean section delivery):   - aOR 0.81 (95% CI 0.67 to 0.99)   Socioeconomic status (reference is family income <200-299% of poverty level):   - Family income < 100% of poverty level aOR 1.76 (95% CI 1.36 to 2.27) - Family income < 100-199% of poverty level aOR 1.43 (95% CI 1.11 to 1.85) - Family income < 300-399% of poverty level aOR 0.89 (95% CI 0.64 to 1.23) - Family income ≥ 400% of poverty level aOR 0.87 (95% CI 0.60 to 1.26) | The odds of short interpregnancy intervals were higher among black mothers than among whites and were inversely associated with income. The odds of short intervals were significantly lower for women with at least a high school education than for those with less education and were reduced for women who were born outside of the United States, had experienced a pregnancy loss following the prior live birth or had delivered their prior infant by caesarean section delivery. |
| de Bocanegra (2014), United States | The purpose of this study was to determine the use of contraceptive methods, which was defined by effectiveness, length of coverage, and their association with short interpregnancy intervals, when controlling for provider type and client demographics. | Women who had second or higher order births and at least 1 Medicaid claim within 18 months after index birth. | 117,644 women | 2002-2008 | Cohort | Provision of contraception | Short IPI (<18 months) | Contraception (reference is barrier methods/supplies):   - Long-acting reversible contraception aHR 3.89 (95% CI 3.55 to 4.26) - User dependent hormonal methods aHR 1.89 (95% CI 1.80 to 1.98) - No contraception aHR 0.66 (95% CI 0.63 to 0.63) | The positive association of optimal birth intervals that corresponds to the method tier demonstrates the advantage of using methods with longer duration and lower rates of contraceptive failure. In the provision of user-dependent methods, an extended length of coverage also facilitates longer interpregnancy intervals. |
| de Bocanegra (2013), United States | To assess the extent to which women received contraceptive service within 90 days after birth at their first or subsequent visit and whether contraceptive provision was associated with optimal interpregnancy intervals. | Women who had second or higher order births and at least 1 Medicaid claim within 18 months after index birth. | 117,644 women | 2002-2008 | Cohort | Postpartum contraception  Family planning program | Short IPI (<6 months)  Sub-optimal IPI (<18 months) | Contraceptive service received vs. no contraceptive service received:  Short IPI (< 6 months)   - Within 90 days aOR 1.98 (95% CI 1.88 to 2.08) - Received at first postpartum visit aOR 1.63 (95% CI 1.49 to 1.80)   Sub-optimal IPI (< 18 months)   - Within 90 days aOR 1.60 (95% CI 1.55 to 1.65) - Received at first postpartum visit aOR 1.57 (95% CI 1.50 to 1.65)   Family planning service vs. Medi-Cal only:  Short IPI (< 6 months)   - Family PACT and Medi-Cal aOR 1.17 (95% CI 1.08 to 1.26) - Family PACT aOR 0.97 (95% CI 0.98 to 1.06)   Sub-optimal IPI (< 18 months)   - Family PACT and Medi-Cal aOR 1.06 (95% CI 1.02 to 1.10) - Family PACT aOR 0.99 (95% CI 0.94 to 1.04) | Receipt of a contraceptive method, receiving contraception at the first clinic visit, and being seen by Medi-Cal and its family planning expansion program were significantly associated with avoidance of short interpregnancy intervals. Receiving contraception at the first postpartum clinic visit had an additional independent effect on avoiding short interpregnancy intervals when controlling for the other variables. |
| Delara (2018), United States | To estimate rates of short IPI among Asian subgroups and Pacific Islanders and associated risk of preterm birth. | Women were included in this study if their first birth occurred in 1999–2000, and if their second birth occurred before 2005 | 189,931 women | 1999-2004 | Cohort | Race/ethnicity | IPI <6 months vs > 18 months  IPI 6-18 months vs > 18 months | Ethnicity (reference is white):  IPI < 6 months   - Black aOR 1.47 (95% CI 1.34 to 1.61) - Hispanic aOR 1.23 (95% CI 1.17 to 1.31) - Pacific Islander aOR 3.31 (95% CI 2.70 to 4.10) - East Asian aOR 1.65 (95% CI 1.48 to 1.84) - Southeast Asian aOR 1.93 (95% CI 1.73 to 2.10) - Filipina aOR 1.51 (95% CI 1.33 to 1.71) - Other Asian aOR 5.01 (95% CI 1.70 to 2.40)   IPI < 18 months   - Black aOR 0.94 (95% CI 0.89 to 0.99) - Hispanic aOR 0.90 (95% CI 0.88 to 0.93) - Pacific Islander aOR 1.35 (95% CI1.16 to 1.57) - East Asian aOR 1.03 (95% CI 0.98 to 1.08) - Southeast Asian aOR 1.08 (95% CI 1.01 to 1.15) - Filipina aOR 0.86 (95% CI 0.81 to 0.92) - Other Asian aOR 1.06 (95% CI 0.96 to 1.18) | Asian and Pacific Islander women have higher rates of IPI < 6 months. |
| El-Kamary (2004), United States | This study aims to assess the impact of home visiting in preventing rapid repeat pregnancies and its malleable determinants and assesses the influence of rapid repeat pregnancies on the mother and the index child. | An at-risk family was eligible for the study if the mother understood English well enough to be interviewed, the family was not already enrolled in the Home Starting Program for a prior birth, and the family was identified on a day when HSP intake was open. | 643 at-risk families | November 1994 - December 1995 | Cohort | Pregnancy intention  Access to family planning site  Contraceptive use  Home visiting program | IBI <24 months | Intended pregnancy vs. unintended pregnancy:   - All mothers aOR 2.86 (95% CI 1.57 to 5.22) - First time mothers are baseline aOR 2.27 (95% CI 0.91 to 5.68)   Lacked access to family planning facility vs. had access to family planning facility:   - All mothers aOR 1.61 (95% CI 0.93 to 2.79) - First time mothers are baseline aOR 0.75 (95% CI 0.32 to 1.79)   Did not use contraception vs. used contraception:   - All mothers aOR 1.67 (95% CI 0.98 to 2.82) - First time mothers are baseline aOR 1.04 (95% CI 0.45 to 2.37)   Enrolled in a home visiting program vs. not enrolled in home visiting program:   - All mothers aOR 1.05 (95% CI 0.69 to 1.58) - First time mothers are baseline aOR 1.23 (95% CI 0.68 to 2.25) | The Hawaii HSP did not reduce rapid repeat pregnancy or alter its malleable determinants. |
| French (2012), United Kingdom and Ireland | To explore the pattern of repeat pregnancies among diagnosed HIV-infected women in the United Kingdom and Ireland, estimate the rate of these sequential pregnancies, and investigate the demographic and clinical characteristics of women experiencing them. | HIV positive women | 14,096 pregnancies to 10,568 HIV-positive women | 1990-2009 | Cohort | Age at first reported pregnancy  Parity at first reported pregnancy  Place of birth | BTP interval <48 months | Age at first pregnancy (reference is < 25 years):   - 25-29 years aHR 0.86 (95% CI 0.77 to 0.97) - 30-34 years aHR 0.73 (95% CI 0.64 to 0.83) - ≥35 years aHR 0.44 (95% CI 0.37 to 0.53)   Parity at first pregnancy (reference is nulliparous):   - Primiparous aHR 0.83 (95% CI 0.75 to 0.93) - Multiparous (2 previous births) aHR 0.79 (95% CI 0.68 to 0.91) - Multiparous (≥3 previous births) aHR 0.65 (95% CI 0.53 to 0.80)   Place of birth (reference is United Kingdom and Ireland):   - Europe aHR 0.73 (95% CI 0.54 to 0.98) - Eastern Africa aHR 0.83 (95% CI 0.72 to 0.95) - Middle Africa aHR 1.29 (95% CI 1.07 to 1.55) - Western Africa aHR 1.36 (95% CI 1.00 to 1.36) - Southern Africa aHR 0.62 (95% CI 0.52 to 0.77) - Africa (unspecified) aHR 0.24 (95% CI 0.11 to 0.51) - Elsewhere aHR 0.85 (95% CI 0.69 to 1.06) | The probability of repeat pregnancy significantly declined with increasing age at first pregnancy. Parity was also inversely associated with repeat pregnancy. Compared with women born in the UK or Ireland, those from Europe, Eastern Africa, and Southern Africa were less likely to have a repeat pregnancy, while women from Middle Africa and Western Africa were more likely to. |
| Gemmill (2013), United States | To investigate the prevalence and correlates of short interpregnancy intervals in the United States. | Women aged 15-44 years | 12,279 women | 2006-2010 | Cohort | Age at most recent birth  Age at first birth  Ethnicity  Medicaid-funded delivery  Pregnancy intention  Education  Marital status  Previous births | Short IPI (< 18 months) | Age at conception of previous pregnancy (reference is 15-19 years)   - 20-29 years aOR 0.27 (95% CI 0.15 to 0.48) - 30-44 years aOR 0.12 (95% CI 0.06 to 0.23)   Age at first reported pregnancy (reference is 15-19 years)   - 20-29 years aOR 0.51 (95% CI 0.28 to 0.92) - 30-44 years aOR 0.41 (95% CI 0.25 to 0.68)   Ethnicity adjusted for age at conception of previous pregnancy (reference is Hispanic):   - Non-Hispanic white aOR 1.35 (95% CI 0.94 to 1.84) - Non-Hispanic black aOR 1.40 (95% CI 0.93 to 2.11) - Non-Hispanic other aOR 1.00 (95% CI 0.56 to 1.79)   Ethnicity adjusted for age at first birth (reference is Hispanic):   - Non-Hispanic white aOR 1.36 (95% CI 0.95 to 1.94) - Non-Hispanic black aOR 1.59 (95% CI 1.06 to 2.39) - Non-Hispanic other aOR 0.92 (95% CI 0.53 to 1.59)   Medicaid adjusted for age at conception of previous pregnancy (reference is non-Medicaid):   - aOR 1.14 (95% CI 0.84 to 1.56)   Medicaid adjusted for age at first birth (reference is non-Medicaid):   - aOR 1.41 (95% CI 1.04 to 1.91)   Pregnancy intention adjusted for age at conception of previous pregnancy (reference is intended pregnancy):   - Mistimed pregnancy aOR 4.43 (95% CI 3.07 to 6.39) - Unintended pregnancy aOR 4.78 (95% CI 3.36 to 6.86)   Pregnancy intention adjusted for age at first birth (reference is intended pregnancy):   - Mistimed pregnancy aOR 2.11 (95% CI 1.37 to 3.27) - Unintended pregnancy aOR 2.21 (95% CI 1.43 to 3.41)   Education at college level or beyond adjusted for age at conception of previous pregnancy (reference is less education):   - aOR 2.31 (95% CI 1.54 to 3.48)   Education at college level or beyond adjusted for age at first birth (reference is less education):   - aOR 1.42 (95% CI 0.94 to 2.41)   Marital status adjusted for age at conception of previous pregnancy (reference not married or cohabiting)   - Married aOR 2.13 (95% CI 1.42 to 3.19) - Cohabiting aOR 1.46 (95% CI 0.93 to 2.30)   Marital status adjusted for age at first birth (reference not married or cohabiting)   - Married aOR 1.85 (95% CI 1.23 to 2.78) - Cohabiting aOR 1.38 (0.90 to 2.13)   Previous births adjusted for age at conception of previous pregnancy (reference is 1 previous birth)   - 2 previous births aOR 1.11 (95% CI 0.79 to 1.57) - ≥3 previous births aOR 1.93 (95% CI 1.31 to 2.86)   Previous births adjusted for age at first birth (reference is 1 previous birth)   - 2 previous births aOR 0.99 (95% CI 0.72 to 1.36) - ≥3 previous births aOR 1.34 (95% CI 0.89 to 2.01) | Short interpregnancy intervals were more likely to be intended among more advantaged women (married, non-Hispanic white, college-educated, or non-Medicaid delivery). |
| Gifford (2021), United States | To evaluate the likelihood of a short interpregnancy interval resulting in a [birth](https://www.sciencedirect.com/topics/medicine-and-dentistry/childbirth) among women covered by Medicaid, as a function of postpartum contraceptive method type. | Women (age 15–44 years old) enrolled in Delaware's  [Medicaid program](https://www.sciencedirect.com/topics/medicine-and-dentistry/medicaid) who had a live [birth](https://www.sciencedirect.com/topics/medicine-and-dentistry/childbirth) during the study period. | 10,328 women | 2012-2014 | Cohort | Use of postpartum LARC | Short IPI (< 12 months) | Contraception use (reference is long-acting reversible contraception):   - No contraception aOR 4.98 (95% CI 3.05 to 8.13) - Effective contraception aOR 3.51 (95% CI 2.13 to 5.77) | Long-acting reversible contraception provision within 60 days postpartum had a strong protective effect against a short IPI, controlling for socio-demographic characteristic. |
| Gold (2004), United States | To examine the relationship between county-level income inequality and pregnancy spacing in a welfare-recipient cohort in Washington State. | Women who received cash assistance from Washington State and had a least one birth. | 20,028 welfare-recipient women | 1^st^ July 1992 – 31^st^ December 1999 | Cohort | Income equality | Short IPI < 18 months) | Total county-level household income (decile 10 vs decile 1):   - Mothers aged ≤25 years aHR 1.24 (95% CI 0.85 to 1.80) - Mothers aged > 25 years aHR 2.14 (95% CI 1.09 to 4.18) | Among women aged 26 or older, increased community income inequality was associated with elevated hazard of both pregnancy and birth. While income inequality is not the only community-level factor that may influence health behaviors, it appears to be associated with the hazard of a subsequent pregnancy even after controlling for other factors. |
| Gottvall (2002), Sweden | The study objective was to examine whether the birth center model of care during a woman’s first pregnancy affects whether or not she has a second baby, and on the spacing to the next birth. | Primiparas of low medical risk | 1,063 women | October 1989 - July 1993 | Randomized controlled trial | Birth centre care | Time to subsequent birth ≤24 months  Time to subsequent birth ≤36 months  Time to subsequent birth ≤72 months | Additional antenatal, intrapartum, and postpartum care vs. standard care from a medical doctor:   - Time to subsequent birth ≤24 months OR 1.1 (95% CI 0.8 to 1.5) - Time to subsequent birth ≤36 months OR 0 (95% CI 0 to 0) - Time to subsequent birth ≤72 months OR 0.8 (95% CI 0.6 to 1.1) | A woman’s model of care, such as birth center care, during her first pregnancy does not seem to be a sufficiently important factor to affect subsequent reproduction in Sweden. |
| Goyal (2017), United States | The objective of the study was to determine the association of home visiting with subsequent pregnancy outcomes. | Eligible participants must be first-time mothers with at least one of four characteristics: unmarried; low income (up to 300% of poverty level, receipt of Medicaid or reported financial concerns); o18 years of age; or suboptimal prenatal care (broadly defined with no specific gestational age cutoff). | 2,920 women | 2007-2009 | Cohort | Home visiting program | Short IPI (<18 months)  Very short IPI (<6 months) | Enrolled in home visiting program vs. not enrolled in home visiting program):  IPI <18 months   - All Latina mothers HR 0.86 (95% CI 0.75 to 0.99) - Mexican mothers HR 0.74 (95% CI 0.49 to 1.07) - Puerto Rican mothers HR 0.87 (95% CI 0.73 to 1.03) - Other Latina mothers HR 0.91 (95% CI 0.59 to 1.33) - Mothers aged ≤18 years HR 0.80 (95% CI 0.65 to 0.96) - Mothers aged >18 years HR 0.95 (95% CI 0.75 to 1.14)   IPI <6 months   - All Latina mothers HR 0.60 (95% CI 0.42 to 0.82) - Mexican mothers HR 0.25 (95% CI 0.00 to 0.72) - Puerto Rican mothers HR 0.62 (95% CI 0.43 to 0.89) - Other Latina mothers HR 0.76 (95% CI 0.30 to 1.62) - Mothers aged ≤18 years HR 0.61 (95% CI 0.38 to 0.92) - Mothers aged >18 years HR 0.59 (95% CI 0.33 to 0.92) | Among those enrolled, moderate vs low participants had reduced risk of repeat pregnancy over 18 months. |
| Gunst (2021), Finland | Testing whether more maternal sleep disturbance was associated with longer IBIs. | Women aged 18-60 years with at least two biological singleton children | 729 women | April 2020 | Cross-sectional | Maternal sleep disturbance  Postpartum depression symptoms | IBI | Maternal sleep disturbance vs. no sleep disturbance:  IBI between the first and second pregnancy   - Age of first child 0-1 years β -0.22 (-0.37 to -0.06) - Age of first child 1-3 years β -0.30 (-0.53 to -0.07)   IBI between the second and third pregnancy   - Age of first child 0-1 years β 0.70 (-0.17 to 0.30) - Age of first child 1-3 years β -0.25 (-0.63 to -0.12)   Postpartum depression symptoms vs. no symptoms:  IBI between the first and second pregnancy   - Age of first child 0-1 years β 0.23 (-0.08 to 0.38) - Age of first child 1-3 years β 0.28 (0.05 to 0.51)   IBI between the second and third pregnancy   - Age of first child 0-1 years β -0.05 (-0.29 to 0.200)   Age of first child 1-3 years β 0.27 (-0.09 to -0.64) | Contrary to our prediction, maternal sleep disturbance was, however, associated with shorter IBIs for the first child when including postpartum depression symptoms in the model. |
| Gupta (2019), Cananda | This study compared the risk for rapid repeat pregnancy in women with versus without schizophrenia. | Ontario women aged 18–49 years with a live birth during the study period | 926,222 women | 1^st^ April 2002 to 31^st^ March 2013. | Cohort | Diagnosis of schizophrenia, schizoaffective disorder, or psychotic disorder | BTP 3 to <12 months  BTP 3 to <24 months | Schizophrenia vs. no Schizophrenia:  BTP 3 to <12 months from previous pregnancy   - Pregnancy results in livebirth, pregnancy loss or abortion aOR 1.31 (95% CI 1.07 to 1.59) - Pregnancy resulting in livebirth aOR 1.85 (95% CI 1.26 to 2.72) - Pregnancy results in pregnancy loss aOR 1.50 (95% CI 0.99 to 2.29) - Pregnancy resulting in abortion aOR 1.07 (95% CI 0.81 to 1.42)   BTP 3 to <24 months from previous pregnancy   - Pregnancy results in livebirth, pregnancy loss or abortion aOR 0.95 (95% CI 0.85 to 1.06) - Pregnancy resulting in livebirth aOR 0.92 (95% CI 0.80 to 1.06) - Pregnancy results in pregnancy loss aOR 1.10 (95% CI 0.84 to 1.44) - Pregnancy resulting in abortion aOR 0.93 (95% CI 0.75 to 1.15) | Women with schizophrenia are at higher risk than their peers for rapid repeat pregnancy but use non-barrier contraception at similar rates. |
| Harney (2017), United States | The study identified risk factors for conception after a short IPI among postpartum women who plan long-acting reversible contraception (LARC). | Women were included if they delivered in the second or third trimester and indicated at time of hospital discharge that they wanted interval LARC placement (implant or intrauterine device). | 3,548 women | January 2005 -December 2010 | Cohort | Use of LARC | Short IPI (<18 months) | No long-acting reversible contraception within 6 months of previous birth vs. long-acting reversible contraception:   - aOR 4.8 (95% CI 3.5 to 6.7) | Women who receive postpartum LARC are less likely to become pregnant after a short IPI. Missing the postpartum visit, multiparity and plan for a contraceptive bridge may all contribute to a patient not receiving planned LARC. |
| Heaman (2018), Canada | The objective of this study was to examine the association of prenatal care utilization with maternal, fetal, and infant outcomes in Manitoba. | Singleton births in Manitoba. | 67,076 singleton births | 2004-2005 and 2008-2009 | Cohort | Inadequate prenatal care  Intensive prenatal care | Short IPI (<12 months) | Inadequate prenatal care vs. adequate prenatal care:   - aOR 1.33 (95% CI 1.25 to 1.43)   Intensive prenatal care vs. adequate prenatal care:   - aOR 0.92 (95% CI 0.81 to 1.06) | Inadequate prenatal care was associated with significantly increased odds of a short interpregnancy interval to the subsequent pregnancy and was used as an indicator of suboptimal pregnancy spacing. Intensive prenatal care was not significantly related to interpregnancy interval |
| Holowko (2018), Australia | To investigate the association of socioeconomic position (SEP) with reproductive outcomes among Australian women | Women born between 1973-1978 | 6,899 women | 1996, 2000, 2003, 2006, 2009, 2012, 2015 | Cohort | Maternal education  Financial stress (ability to manage own income)  Socioeconomic status  Grandparent's highest education | BTP interval <18 months  BTP interval 28-59 months  BTP interval >59 months | Maternal education (reference is high level):  BTP interval <18 months vs. BTP interval 18-27 months   - Intermediate level aOR 0.9 (95% CI 0.8 to 1.1) - Low level aOR 1.1 (95% CI 0.9 to 1.3)   BTP interval 28-59 months vs. BTP interval 18-27 months   - Intermediate level aOR 1.5 (95% CI 1.2 to 1.9) - Low level aOR 1.2 (95% CI 0.9 to 1.5)   BTP interval >59 months vs. BTP interval 18-27 months   - Intermediate level aOR 1.5 (95% CI 1.0 to 2.3) - Low level aOR 1.0 (95% CI 0.6 to 1.5)   Financial stress (reference is not too bad/easy):  BTP interval <18 months vs. BTP interval 18-27 months   - Sometimes difficult aOR 0.9 (95% CI 0.8 to 1.1) - Impossible/always difficult aOR 1.1 (95% CI 0.9 to 1.4)   BTP interval 28-59 months vs. BTP interval 18-27 months   - Sometimes difficult aOR 0.9 (95% CI 0.7 to 1.1) - Impossible/always difficult aOR 1.2 (95% CI 0.9 to 1.6)   BTP interval >59 months vs. BTP interval 18-27 months   - Sometimes difficult aOR 1.3 (95% CI 0.9 to 1.8) - Impossible/always difficult aOR 2.3 (95% CI 1.5 to 3.5)   Socioeconomic status (per 1 quintile):  BTP interval <18 months vs. BTP interval 18-27 months   - aOR 1.0 (95% CI 0.9 to 1.1)   BTP interval 28-59 months vs. BTP interval 18-27 months   - aOR 1.0 (95% CI 0.9 to 1.1)   BTP interval >59 months vs. BTP interval 18-27 months   - aOR 1.1 (95% CI 1.0 to 1.3)   Grandparent education (reference is completed college or more):  BTP interval <18 months vs. BTP interval 18-27 months   - Trade/certificate/diploma aOR 1.5 (95% CI 1.1 to 2.0) - ≤12 years aOR 1.6 (95% CI 1.1 to 2.3) - ≤10 years aOR 1.8 (95% CI 1.4 to 2.5) - Don’t know/NA aOR 4.5 (95% CI 3.2 to 6.4)   BTP interval 28-59 months vs. BTP interval 18-27 months   - Trade/certificate/diploma aOR 0.8 (95% CI 0.6 to 0.9) - ≤12 years aOR 0.8 (95% CI 0.7 to 1.1) - ≤10 years aOR 0.6 (95% CI 0.5 to 0.8) - Don’t know/NA aOR 0.6 (95% CI 0.4 to 0.8)   BTP interval >59 months vs. BTP interval 18-27 months   - Trade/certificate/diploma aOR 0.7 (95% CI 0.5 to 0.9) - ≤12 years aOR 0.7 (95% CI 0.5 to 0.9) - ≤10 years aOR 0.5 (95% CI 0.4 to 0.6) - Don’t know/NA aOR 0.5 (95% CI 0.3 to 0.8) | Low socio-economic position was associated with having a longer than recommended BTP interval. |
| Iacobelli (2014), France | To evaluate longitudinal care needs and health service access among mother–infant pairs after adolescent pregnancy | Adolescent and adult mother–infant pairs who delivered at the tertiary care maternity department of Saint Pierre, Reunion Island University Hospital, France | 952 mother-infant pairs (208 cases; 525 controls) | 1^st^ January 2004-31^st^ December 2006 | Case-control | Adolescent pregnancy | IBI <18 months | < 18 years at first singleton birth vs. 18-29 years:   - OR 2.9 (95% CI 1.5 to 5.6) | Adolescent mothers had significantly higher numbers of pregnancies and child-births, with higher rates of rapid repeat pregnancy and shorter times be-tween successive pregnancies. |
| Isquick (2017), United States | To determine the association of age at index birth with postpartum contraceptive use and optimal interpregnancy interval (IPI, defined as delivery to next pregnancy [18 months), controlling for provider type and client demographics among adolescent mothers who have repeat pregnancies. | Mothers accessing Medi-Cal and Family PACT services, whose index birth occurred as an adolescent and who had a subsequent birth within 6 years | 26,393 mothers | 2002-2008 | Cohort | Postpartum contraception | IPI >18 months | Contraception (reference is barrier methods/supplies):   - Long-acting reversible contraception aOR 4.25 (95% CI 3.47 to 5.21) - User-dependent hormonal methods aOR 2.10 (95% CI 1.89 to 2.32) - No contraception aOR 0.70 (95% CI 0.62 to 0.78) - Coverage of user-dependent hormonal methods aOR 1.1 (95% CI 1.09 to 1.11) | Compared to those using only barrier methods, adolescent women receiving highly effective contraceptive methods had a 4.25 times higher odds of having an optimal IPI than those receiving hormonal methods, or using no method. |
| Kaharuza (2001), Denmark | The study objective was to identify risk factors associated with short interpregnancy intervals in Denmark. | Pregnant women within the geographical area of Odense and Aalborg cities. | 11,288 women | April 1984 - April 1987 | Cohort | Maternal age  Maternal education  Marital status  Parity at index birth  Smoking during pregnancy  Menstrual regularity  Social class (based on mother's and father's occupational status)  Type of housing  Unplanned pregnancy | Short IPI (≤9 months) | Maternal age (reference is 26-30 years):   - < 20 years aOR 0.72 (95% CI 0.41 to 1.26) - 21-25 years aOR 0.75 (95% CI 0.54 to 1.03) - 31-49 years aOR 1.67 (95% CI 1.11 to 2.51)   Maternal education (reference is ≥13 years):   - < 10 years aOR 1.24 (95% CI 0.80 to 1.93) - 10-12 years aOR 1.13 (95% CI 0.82 to 1.57)   Marital status vs. single:   - aOR 1.14 (95% CI 0.84 – 1.54)   Parity at index birth (reference is 1 previous birth):   - 2 previous births aOR 1.18 (95% CI 0.75 to 0.93) - 3 previous births aOR 1.87 (95% CI 1.11 to 3.14)   Smoking during pregnancy (reference is did not smoke):   - Smoked 1-10 cigarettes/day aOR 1.00 (95% CI 0.67 to 1.48) - Smoked 11-20 cigarettes/day aOR 1.21 (95% CI 0.81 to 1.81) - Smoked > 20 cigarettes/day aOR 1.57 (95% CI 0.98 to 2.50)   Irregular menstrual cycle vs regular menstrual cycle:   - aOR 1.69 (95% CI 1.13 to 2.54)   Mothers and fathers occupational status (reference is management):   - Skilled/unskilled manual labor aOR 0.72 (95% CI 0.46 to 1.13) - Student aOR 1.14 (95% CI 0.26 to 4.97) - Unemployed aOR 1.83 (95% CI 1.33 to 2.54)   Type of housing (reference is bungalow):   - Semi-detached aOR 1.29 (95% CI 0.88 to 1.89) - Flat aOR 1.72 (95%CI 1.13 to 2.64) - Rural aOR 1.65 (95% CI 1.09 to 2.49) - Other aOR 1.25 (95% CI 0.550 to 3.14)   Unplanned pregnancy vs intended pregnancy:   - aOR 2.89 (95% CI 2.16 to 3.87) | Short interpregnancy intervals may be a marker for women at risk and these risk factors differ among populations. They also appear to be a result of choice (e.g. in older women). Biological factors also play a significant role in determining short interpregnancy intervals. |
| Kan (2012), United States | To evaluate the effectiveness of care demonstration projects supported by the Title XX Adolescent Family Life (AFL) program, which serves pregnant and parenting adolescents in an effort to mitigate the risks associated with adolescent childbearing. | Adolescent women aged ≤19 years attending twelve pregnant and parenting adolescent projects in 10 states | 1,038 adolescent women | August 2008 - April 2010 | Randomised controlled trial | Pregnant and parenting adolescent support program | IBI ≤12 months  IBI >12 months | Enrolment in adolescent pregnancy and parenting support program vs. not enrolled:   - IBI ≤12 months OR 0.39 (95% CI 0.16 to 0.98) - IBI >12 months OR 1.39 (95% CI 0.84 to 2.30) | Odds of a repeat pregnancy were lower among intervention group adolescents than among comparison group adolescents within 12 months of intake. |
| Katz (2011), United States | The objective of this study was to compare time to a repeat pregnancy between the intervention and usual care groups, and, secondarily, to determine whether treatment intensity influenced time to subsequent conception. | Primiparous pregnant teens ages 15–19, were recruited in Washington, DC | 249 teenage women aged 15-19 years | November 2005 - August 2009 | Randomised controlled trial | Phone-based counselling intervention  Intensity of intervention (increasing % of completed intervention sessions) | IBI ≤12 months | Phone-based counselling intervention vs. usual care:   - aHR 0.86 (95% CI 0.55 to 1.32)   Intensity of intervention vs. usual care:   - Aged 15-17 years at delivery aHR 0.99 (95% CI 0.98 to 1.00) - Aged ≥ 18 years aHR 1.01 (95% CI 0.99 to 1.02) | Participants who were aged 15–17 years at delivery showed a significant reduction in subsequent pregnancy with increased levels of intervention exposure, but not those ≥ 18 years. |
| Knutson (2022), United States | Aimed to evaluate factors associated with early resumption of sexual intercourse after first childbirth and assess whether early intercourse is associated with unprotected intercourse, subsequent pregnancy, and unintended pregnancy over 6 months | Eligibility criteria included having a singleton pregnancy; being nulliparous, aged 18–35 years, and English or Spanish speaking; and planning to deliver in a hospital in Pennsylvania. | 2,643 women | 2009-2011 | Cohort | Resumption of sexual intercourse | BTP interval <6 months | Resumption of sexual intercourse <12 months of previous birth vs later resumption of sexual intercourse ≥12 months of previous birth:   - All pregnancies aOR 3.03 (95% CI 1.48 to 6.20) - Intended pregnancies aOR 3.32 (95% CI 1.50 to 7.36) | Early resumption of intercourse was associated with a greater likelihood of unprotected intercourse and unintended pregnancy within 6 months of first childbirth. |
| Krans (2018), United States | The purpose of this study was to describe postpartum contraceptive utilization patterns among women with OUD and evaluate the relationship between postpartum contraceptive method choice and interpregnancy interval. | Women aged 15-45 years | 9,260 pregnancies to 7,805 women | 2008-2013 | Cohort | Contraceptive method  Alcohol consumption  Smoking during pregnancy  Drug use during pregnancy  Medication use during pregnancy  Parity  Race/ethnicity  Medical co-morbidities | Short IPI (≤18 months) | Contraception use (reference is no contraception):   - Female sterilisation OR 0.02 (95% CI 0.00 to 0.11) - Long-acting reversible contraception OR 0.37 (95% CI 0.19 to 0.75) - Effective contraception (oral contraception, injection, vaginal ring, patch) OR 0.95 (95% CI 0.80 to 1.14)   Alcohol consumption vs. no alcohol:   - OR 0.90 (95% CI 0.67 to 1.20)   Smoking during pregnancy vs. not smoking:   - OR 0.95 (95% CI 0.81 to 1.10)   Drug use during pregnancy vs. no drug use):   - OR 1.01 (95% CI 0.84 to 1.22)   Medication use during pregnancy (reference is no medication use):   - Methadone OR 0.95 (95% CI 0.80 to 1.12) - Buprenorphine OR 1.06 (95% CI 0.88 to 1.28)   ≥ 2 previous pregnancies vs. 1 pervious pregnancy:   - OR 1.96 (95% CI 1.62 to 2.36)   Race/ethnicity (reference is white):   - Black OR 1.25 (95% CI 0.97 to 1.61) - Hispanic OR 1.18 (95% CI 0.70 to 1.98) - Asian OR 1.46 (95% CI 0.38 to 5.64) - Other OR 0.98 (95% CI 0.57 to 1.68)   Maternal asthma vs. no asthma:   - OR 1.17 (95% CI 0.95 to 1.44)   Chronic hypertension vs. no chronic hypertension:   - OR 1.63 (95% CI 1.08 to 2.46)   Diabetes mellitus vs. diabetes mellitus:   - OR 1.38 (95% CI 0.77 to 2.47)   Gestational diabetes mellitus vs. no gestational diabetes mellitus):   - OR 1.16 (95% CI 0.85 to 1.58)   Gestational hypertension disorder vs. no gestational hypertension disorder:   - OR 0.76 (95% CI 0.58 to 1.01)   Hepatitis C vs. no Hepatitis C:   - OR 0.92 (95% CI 0.78 to 1.09)   HIV status vs no HIV status:   - OR 0.36 (95% CI 0.09 to 1.57)   Psychiatric disorder vs. no psychiatric disorder:   - OR 0.99 (95% CI 0.85 to 1.15)   Thyroid disorder vs. no thyroid disorder:   - OR 0.82 (95% CI 0.48 to 1.40) | Importantly, no significant differences were found in the time to next pregnancy among women who used an effective contraceptive method and women with no method observed during the first 3 months postpartum. The use of LARC methods in the postpartum period was significantly associated with a lower hazard of having a subsequent pregnancy compared to the use of an effective method or no method observed. |
| Lane (2008), United States | This study investigates the persistent relationships between childhood lead exposure, repeat teen pregnancy, and tobacco use in a sample of teenage females in Syracuse, NY. | Teenage White and African American women aged 15-19 years | 536 women | 1998-2002 | Cohort | Lead poisoning during childhood (≥20 μg/dl vs. 0-19 μg/dl) | RRP | aOR 1.59 (95% CI 1.04 to 2.43) | The mothers’ childhood lead exposure, controlling for race, age, and Medicaid status, was associated with repeat teen pregnancy and tobacco use. |
| Lewis (2010), Australia | To examine the determinants of pregnancy within 2 years of a teenager giving birth for the first time (rapid-repeat pregnancy [RRP]) and resumption of sexual intercourse after the birth. | Nulliparous, English-speaking teenagers aged 12- 18 years or younger who attended the adolescent antenatal clinic King Edward Memorial Hospital | 147 teenagers | June 2004 - September 2006 (first births) | Cohort | Contraceptive method  Pregnancy intention  Ongoing sexual intercourse >3 months  Indigenous status | BTP interval <24 months | Contraceptive use (reference is no contraception):   - Oral contraception aOR 0.91 (95% CI 0.39 to 2.21) - Long-acting contraception aOR 0.27 (95% CI 0.12 to 0.62)   Mistimed pregnancy vs. intended pregnancy):   - aOR 2.39 (95% CI 1.62 to 4.93)   Ongoing sexual intercourse >3 months after birth vs. No/less frequent sexual intercourse >3 months after birth:   - aOR 8.96 (95% CI 1.97 to 40.74)   Indigenous status vs. non-Indigenous):   - aOR 2.38 (95% CI 1.38 to 4.11) | Use of long-acting contraceptives reduces the incidence of rapid repeat pregnancy. |
| Liberty (2020), United States | To examine the impact of the Medicaid policy change on the initiation of long-acting and reversible contraception (immediate postpartum and postpartum) within key populations | Singleton births < 23 gestational weeks not covered by emergency Medicaid | 51,320 interpregnancy intervals | 1^st^ January 2010 – 31^st^ July 2017 | Cohort | Use of contraception | Short IPI (<18 months) | Contraception use (reference is no contraception):   - Immediate long-acting reversible contraception aOR 0.62 (95% CI 0.44 to 0.89) - Long-acting reversible contraception aOR 0.31 (95% CI 0.28 to 0.33) | Utilization of immediate postpartum long-acting and reversible contraception was associated with a decreased odds of a subsequent short interpregnancy interval. |
| Lindsay (1995), United States | To examine relationships among human immunodeficiency virus (HIV) serostatus, postpartum contraceptive choice, and the rate of repeat pregnancy within a short interval. | 83 seropositive and 218 seronegative women identified from an inner-city prenatal population undergoing routine voluntary HIV antibody screening. | 301 women | July 1987 - December 1989 | Cohort | HIV status | RRP | HIV vs non-HIV   - OR 54.7 (95% CI -6.30 to 115.6) | There was a relationship between the method of postpartum contraception and HIV serostatus, but no significant difference in repeat pregnancy rates associated with choice of method. |
| Liu (2022), United States | Aimed to evaluate if the expansion affected the risk of having a short interpregnancy interval (IPI), a preventable risk factor for adverse pregnancy outcomes | Parous women aged 19 or older (being adult at the time of conception) who had a singleton live birth with known gestational age and year and month of the prior birth and IPI >2 months | 14,873,995 births across 43 states and DCs | 2009-2018 | Cross-sectional | ACA Medicaid Expansion | Short IPI (<12 months) | Reported adjusted results with 95% CI for Difference-in-Differences   - All 1.24 (95% CI -1.64 to 4.12) | The expansion was not associated with a significant change in risk of having a short IPI. |
| Mishra (2022), Australia | The aim was to examine maternal factors associated with  birth intervals in Australia. | Women who were born in 1973-1978,  had two or more births,  and responded to regular surveys between 1996 and 2018. | 6,130 women | 1996-2018 | Cohort | Maternal age  Contraception usage  Alcohol consumption  Physical activity level  BMI  Education  Fertility problems  Financial stress  Marital status  Occupation  Place of residence  Smoking during pregnancy  Self-rated health  Self-rated mental health  History of miscarriage  History of abortion | IBI  *only reported results for first to second pregnancy | Maternal age (reference is 25-30 years):   - <25 years AF 0.84 (95% CI 0.77 to 0.90) - 30-35 years AF 1.19 (95% CI 1.14 to 1.23) - >35 years AF 1.42 (95% CI 1.36 to 1.48)   Contraception usage vs. no contraception:   - AF 0.94 (95% CI 0.91 to 0.98)   Alcohol consumption (reference is low risk drinker):   - Non-drinker AF 0.96 (95% CI 0.92 to 1.01) - Rarely drinks AF 1.04 (95% CI 1.01 to 1.08) - Risky and heavy drinker AF 1.07 (95% CI 0.95 to 1.21)   Physical activity level (reference is moderate):   - Nil/sedentary AF 1.01 (95% CI 0.95 to 1.07) - Low AF 0.94 (95% CI 0.91 to 0.98) - High AF 1.07 (95% CI 1.02 to 1.13)   BMI (reference is healthy 18.5 < 25 kg/m2):   - Underweight <18.5 AF 1.19 (95% CI 1.10 to 1.28) - Overweight 25-30 AF 1.02 (95% CI 0.98 to 1.06) - Obese > 30 AF 1.04 (95% CI 1.00 to 1.08)   Education (reference is ≤12 years):   - Trade/apprenticeship AF 0.98 (95% CI 0.65 to 1.24) - University/higher degree AF 0.87 (95% CI 0.83 to 0.91)   Fertility problems (reference is no fertility problems):   - Never tried to become pregnant AF 0.98 (95% CI 0.91 to 1.05) - Yes, and did not seek help AF 1.27 (95% CI 1.18 to 1.37) - Yes, but sought help/treatment AF 1.05 (95% CI 1.01 to 1.09)   Financial stress (reference is impossible/difficult to manage):   - Sometimes difficult AF 0.89 (95% CI 0.84 to 0.94) - Not bad AF 0.86 (95% CI 0.82 to 0.91) - Easy AF 0.80 (95% CI 0.76 to 0.85)   Marital status (reference is married):   - De facto AF 1.13 (95% CI 1.08 to 1.17) - Separated/divorced/widowed AF 2.01 (95% CI 1.79 to 2.27) - Single AF 1.32 (95% CI 1.22 to 1.43)   Occupation (reference is manager/professional):   - Trade/associate professional/intermediate AF 0.99 (95% CI 0.83 to 1.05) - Elementary /laborer AF 1.11 (95% CI 1.05 to 1.16) - No paid job AF 0.91 (95% CI 0.88 to 0.95)   Place of residence (reference is major cities):   - Inner regional AF 1.05 (95% CI 1.02 to 1.08) - Outer regional AF 1.06 (95% 1.02 to 1.10) - Remote/very remote AF 1.01 (95% CI 0.93 to 1.09)   Smoking during pregnancy (reference is never):   - Former AF 1.00 (95% CI 0.96 to 1.03) - Current AF 1.15 (95% CI 1.11 to 1.20)   Self-rated health (reference is excellent):   - Very good AF 1.07 (95% CI 1.03 to 1.12) - Good AF 1.15 (95% CI 1.10 to 1.21) - Fair AF 1.17 (95% CI 1.09 to 1.26)   Self-rated mental health using the SF26 (reference is ≥52):   - <52 AF 1.05 (95% CI 1.00 to 1.10)   History of miscarriage (reference is no miscarriage):   - Yes AF 1.07 (95% CI 1.04 to 1.11)   History of abortion (reference is no abortion):   - Yes AF 1.07 (95% CI 1.03 to 1.12) | Shorter IBI associated with  higher socioeconomic status (eg, university education), less income  stress, and longer IBI associated with age over 35, fair/poor health, untreated fertility problems, miscarriage, or abortion. |
| Olorunsaiye (2023), United States | The purpose of this study was to examine the associations between multiple immigration-related variables and interbirth intervals among reproductive -aged immigrant and refugee women living in the United States. | Eligibility for the online survey was based on self-identification as a woman, aged 18–44 years, English proficiency, and residency in the United States. | 653 women | Unclear | Cross-sectional | Immigration pathway  Length of time since immigration  Birthplace | IBI (≤18 months) | Immigration pathway (reference is US-born):   - Conflict/security aOR 0.86 (95% CI 0.23 to 3.20) - Education aOR 4.57 (95% CI 1.57 to 9.58) - Employment aOR 2.27 (95% CI 1.07 to 5.31) - Family-based aOR 0.36 (95% CI 0.03 to 3.74)   Length of time since immigration (reference is US-born):   - ≤5 years aOR 0.50 (95% CI 0.23 to 1.01) - 6-10 years aOR 0.34 (95% CI 0.03 to 3.77) - >10 years aOR 0.41 (95% CI 0.03 to 4.74)   Birthplace (reference is US-born)   - Africa aOR 0.79 (95% CI 0.02 to 0.98) - Asia aOR 3.18 (95% CI 0.27 to 37.73) - Other Western countries aOR 2.72 (95% CI 0.21 to 34.55) - South and Central America aOR 3.40 (95% CI 0.32 to 7.07) - Caribbean aOR 1.52 (95% CI 0.15 to 15.70) | Women who immigrated to the United States for educational or employment opportunities had higher odds of reporting a longer interbirth interval (19–35 or ≥36 months) than women born in the United States. Women born in an African country had 0.79 times the odds of being in a higher category of interbirth interval. |
| Patel (2021), United States | To assess the influence of Medicaid on short interpregnancy intervals. | Multiparous women who had live births during the study period. | 3,349,768 births | 2012-2016 | Cohort | Coverage of contraception | Short IPI (<12 months) | Implementing US states in 2016 (reference is Non-implementing US states in 2016):   - aRR 0.94 (95% CI 0.97 to 0.98)   Implementing US states in 2012 (reference is Implementing US states in 2016):   - aRR 1.02 (95% CI 1.01 to 1.03)   Non-implementing US states in 2012 (reference is non-implementing US states in 2016)   - aRR 1.03 (95% CI 1.03 to 1.04) | The risk of short nterpregnancy intervals decreased in the Medicaid expansion states even after adjusting for risk factors. Moreover, the short nterpregnancy interval rates increased in non-expansion states but decreased in Medicaid expansion states. |
| Plana-Ripoll (2018), Denmark and Sweden | To examine women’s reproductive trajectory following the death of a child. | Women who were born in Denmark and Sweden | 1,979,958 women: 902,098 (Denmark) and 1,077,860 (Sweden) | 1978-2004 (Denmark) and 1973-2002 (Sweden) | Cohort | Loss of previous child (including stillbirth) | IPI < 6 months | aHR 5.5 (95% CI 5.41 to 5.59) | Compared with unexposed women, exposed women had a shorter interpregnancy interval and, consequently, a higher rate of conception leading to a birth. |
| Raneri (2007), United States | To evaluate the incidence of repeat pregnancy within 24 months of delivery in a large, multiethnic sample of adolescent mothers who were followed prospectively for four years and to identify multilevel predictors of subsequent pregnancy. | Caucasian, Black, and Mexican-American adolescent mothers aged 12-18 years | 932 adolescent mothers | December 8, 1993 - February 28, 1996 | Cohort | Pregnancy intention ≤5 years  Not enrolled in school ≤3 months of previous birth  Relationship status with father of first child ≤3 months of previous birth  >3 years older than the biological mother of the child  Contraceptive method  Domestic violence by boyfriend/husband ≤3 months of previous birth  ≥50% of friends were teenage mothers at delivery | BTP interval <24 months | Intended pregnancy ≤5 years from previous birth vs. unintended pregnancy:   - OR 1.55 (95% CI 1.03 to 2.34)   Not enrolled in school <3 months after previous birth vs. Enrolled in school <3 months after previous birth   - OR 1.75 (95% CI 1.20 to 2.55)   Not in a relationship with biological father of first child >3 months after birth vs. Partnered   - OR 2.04 (95% CI 1.37 to 3.05)   >3 years older than the biological mother of the child vs. ≤3 years older than the biological mother of the child  - OR 1.60 (95% CI 1.10 to 2.35)  Pre-pregnancy intimate partner violence <3 months after the previous birth vs. No violence   - OR 1.85 (95% CI 1.18 to 2.88)   ≥50% of friends were adolescent mothers at previous birth vs. <50% of friends were adolescent mothers at previous birth   - OR 1.52 (95%CI 1.03 to 2.26) | Failure to initiate a long-acting contraceptive m within three months of delivery was the strongest predictor of rapid repeat pregnancy. |
| Reese (2017), United States | To examine distal factors—school, family, peers, and public/private religious ties—and their associations with RRP among adolescent mothers. | Adolescents in grades 7–12 | 1,158 adolescent women | 1994-1995 | Cohort | Attachment to family and peers  Attachment to church  Frequency of participation in youth activities | BTP interval <24 months | Attachment to family and peers:   - Parent-adolescent relationship quality aOR 0.83 (95% CI 0.71 to 0.99) - Adolescent perceived maternal disapproval of sex OR 1.06 (95% CI 0.88 to 1.26) - Anticipated social consequences of sex OR 1.18 (95% CI 1.02 to 1.35)   Attachment to Church:   - Prayed once a week vs. Prayed once a day or more OR 0.47 (95% CI 0.18 to 1.24) - Prayed once a month vs. Prayed once a day or more OR 0.79 (95% CI 0.49 to 1.29) - Prayed less than once a month vs. Prayed once a day or more OR 1.33 (95% CI 0.88 to 2.02) - Never prayed vs. Prayed once a day or more OR 1.47 (95% CI 1.10 to 1.96)   Frequency of participation in youth activities:   - Once a month or more vs. Once a week or more OR 0.96 (95% CI 0.94 to 1.02) - Less than once a month vs. Once a week or more OR 1.08 (95% CI 0.71 to 1.89) - Never vs. Once a week or more OR 1.04 (95% CI 1.01 to 1.07) | Attachments to conventional institutions were associated with lower likelihood of RRP. |
| Rozario (2021), United States | This study examines the relationship between pre-pregnancy IPV and short IBI. | Multiparous women who provided a valid response to all three key variables—IPV, IBI, and insurance status—either through birth certificate data or on the survey questionnaire. | 13,675 women | 2009-2011 | Cross-sectional | Pre-pregnancy intimate partner violence | Short IBI (<36 months) | Pre-pregnancy intimate partner violence after the previous birth vs. No violence:   - aOR 2.37 (95% CI 1.33 to 4.23) - Privately insured women aOR 1.34 (95% CI 0.85 to 2.40) - Women on Medicaid/public insurance aOR 2.50 (95% CI 1.04 to 5.92) - Uninsured women aOR 3.36 (95% CI 1.02 to 8.02) | Findings from this study strengthen the evidence that women who experience IPV before pregnancy are significantly more likely to have short IBI compared to women who do not experience pre-pregnancy IPV. Furthermore, the odds of short IBI is highest among women experiencing pre-pregnancy IPV who are uninsured or on Medicaid/public insurance. |
| Rubin (2011), United States | To examine, following statewide dissemination, the influence of an evidence-based home visitation program for first-time mothers on reductions of subsequent pregnancies across time and different locations. | Women who o delivered a first-born singleton infant between January 1, 2000, and December 31, 2005, and who received any form of welfare assistance from the Commonwealth of Pennsylvania within 12 months prior to the infant’s birth | 14,782 women | 1^st^ January 2000 – 31^st^ December 2007 | Cohort | Prenatal home visitation program  Time period of enrolment (later vs. earlier clients) | IPI <15 months | Time period: 2000-2003  Enrolment in prenatal home visitation program vs. No enrolment   - aOR 1.09 (95% CI 0.95 to 1.26)   Time period: 2004-2005  Enrolment in prenatal home visitation program vs. No enrolment   - aOR 0.87 (95% CI 0.80 to 0.96)   2004-2005 enrolment vs. 2000-2003 enrolment   - aOR 0.80 (95% CI 0.66 to 0.96) | Program effects on pregnancy planning emerged after an implementation period of 3 years in both urban and rural locations, but they were particularly strong in rural locations and among younger mothers. |
| Smith (2006), United Kingdom | To determine whether cesarean delivery is independently associated with later subfertility. | Women who had first singleton births between 1980-1984 | 79.304 women | 1980-1999 | Cohort | Mode of delivery | IPI – definition of shorter and longer IPI not declared | Vaginal breech vs. Spontaneous vertex delivery   - aDID 54.7 (95% CI -6.3 to 115.6)   Assisted vaginal vs. Spontaneous vertex delivery   - aDID 28.7 (95% CI 18.5 to 39.0)   Elective caesarean section for breech vs. Spontaneous vertex delivery   - aDID112.7 (95% CI 77.2 to 148.2)   All other pre-labour caesarean section vs. Spontaneous vertex delivery   - aDID 89.5 (95% CI 62.2 to 116.7)   Emergency caesarean section vs. Spontaneous vertex delivery   - aDID 68.4 (95% 51.3 to 85.6) | It is unlikely that delivering by cesarean section in a first pregnancy decreases a woman’s likelihood of having a second viable pregnancy. |
| Steenland (2022), United States | To examine the association between South Carolina’s policy change and infant health. | All index births that were paid for by Medicaid between January 2009 and December 2015 | 186,953 births | January 2009 - December 2019 | Cohort | Reproductive factors | IBI <4 years  Short IBI (≤15 months) | IBI <4 years % point   - all -1.6 (-2.8 to -0.3) - Non-Hispanic black -2.7 (-5.6 to 0.3) - Non-Hispanic white -1.1 (-2.5 to 0.3) - Non-Hispanic Black vs. Non-Hispanic White -1.54 (-5.03 to 1.95)   IBI <15 months % point   - all -0.2 (-0.7 to 0.2) - Non-Hispanic black -0.6 (-1.2 to -0.1) - Non-Hispanic white -0.1 (-0.7 to -0.5) - Non-Hispanic Black vs. Non-Hispanic White –0.55 (-1.09 to 0) | The policy was associated with a 0.6–percentage point (95% CI, −1.2 to −0.1) decrease in the probability of short-interval birth and a 27-day (95% CI, 11-44) increase in days to next birth among non-Hispanic Black individuals. |
| Stevens-Simon (1999), United States | To assess whether adoption of the contraceptive implant would lower the rate of repeat pregnancy, contraceptive use and pregnancy outcomes | Adolescent women aged 13-18 years who received care through the Colorado Adolescent Maternity Program | 309 adolescents | 1992-1993 | Cohort | Early postpartum contraceptive implant | BTP interval <12 months  BTP interval <24 months | No or other contraceptive method vs. Contraceptive implant within 6 months of previous birth   - BTP interval <12 months aOR 35.2 (95% CI 4.48 to 276.4) - BTP interval <24 months aOR 8.58 (95% CI 4.31 to 17.06) | Although early implant insertion significantly decreased the rate of rapid, repeat adolescent pregnancies, the rates of removal and of pregnancy by the end of the second year postpartum were high. |
| Waynforth (2015), United Kingdom | To test whether birth intervals were shorter following the birth of a child with a long-term health problem. | 42-year old women from the BCS70 sample | 4,543 births | 1988-2012 | Cohort | Birth order >3  Marital break-up in interval  Plurality  Family size  Serious health problem | IBI | Birth order >3 vs. Birth order ≤3   - β -1.93 (-2.11 to -1.75)   Marital or relationship dissolution vs. No dissolution   - β -0.54 (-0.068 to -0.4)   Plural birth vs. Singleton birth   - β -1.17 (-1.59 to -0.76)   Larger family size (>4 children) vs. Smaller family size (≤3 children)   - β 1.07 (0.87 to 1.26)   Serious medical condition diagnosed <1 year of age vs. No diagnosis   - β 0.03 (0.1 to 1.41) | Birth intervals were shorter following the birth of a child diagnosed by a medical professional with a severe but non-fatal medical condition |
| White (2015), United States | To investigate women's patterns of contraceptive use after delivery and the association between method use and risk of pregnancy within 18 months | Women aged 15-44 years who had delivered a live-born singleton | 3,005 births | 2006-2010 (first birth) | Cohort | Educational attainment  Marital status  Ethnicity  Parity  Payment for delivery    Contraceptive method  Maternal age | Short IPI (<18 months) | Educational attainment (reference is high school or some college):   - Less than high school aHR 1.36 (95% CI 1.01 to 1.83) - College degree aHR 1.12 (95% CI 0.75 to 1.66)   Married or cohabiting vs. Not married   - aHR 0.73 (95% CI 0.54 to 0.98)   Ethnicity (reference is white):   - Black aHR 0.96 (95% CI 0.68 to 1.3) - Latina aHR 0.84 (95% CI 0.58 to 1.21) - Other aHR 0.80 (95% CI 0.48 to 1.33)   Number of living children (reference is 2 children):   - 1 child aHR 1.30 (95% CI 0.87 to 1.95) - ≥3 children aHR 1.17 (95% CI 0.73 to 1.87)   Medicaid vs. Private   - aHR 1.18 (95% CI 0.83 to 1.69)   Contraceptive method (reference is long-acting reversible contraception)   - Hormonal aHR 21.2 (95% CI 6.17 to 72.8) - Less effective contraception aHR 34.8 (95% CI 9.26 to 131) - None aHR 43.2 (95% CI 12.3 to 152)   Maternal age (reference is 30-34 years)   - 15-24 years aHR 2.37 (95% CI 1.42 to 3.96) - 25-29 years aHR 1.86 (95% CI 1.20 to 22.90) - 35-44 years aHR 0.98 (95% CI 0.56 to 1.71) | Few women use long-acting reversible contraceptives after delivery, and those using less-effective methods have an increased risk of unintended pregnancy. |
| Woolner (2019), United Kingdom | This study aimed to investigate the reproductive impact of a third- or fourth-degree tear in primigravid women. | Women who had a singleton birth with a record or perineal tear or vaginal trauma | 182,445 women | 1997-2010 | Cohort | Fourth-degree perineal tears  Third- or fourth-degree perineal tears | IPI | Fourth-degree perineal tear during first pregnancy vs. No or third-degree perineal tear during first pregnancy   - aHR 0.98 (95% CI 0.76 – 1.28)   Third- or fourth-degree perineal tear during first pregnancy vs. No or third-degree perineal tear during first pregnancy   - aHR 1.01 (95% CI 0.95 to 1.08) | Women do not delay or avoid childbirth after initial third- or fourth-degree tear. |
| Yun (2014), United States | To examine the impact of a maternal–child home visitation program on birth spacing for first-time Latina mothers, focusing on adolescents and women who identified as Mexican or Puerto Rican. |  | 2,000 Latina women | 200-2007 | Cohort | Home visiting program  Ethnicity | Short IPI (≤18 months)  Very short IPI (≤6 months) | Short IPI (≤6 months)   - all HR 0.60 (95% CI 0.42 to 0.82) - Mexican aHR 0.25 (95% CI 0.00 to 0.72) - Puerto Rican aHR 0.62 (95% CI 0.43 to 0.89) - Other Latina aHR 0.76 (95% CI 0.30 to 0.92) - ≤18 years aHR 0.61 (95% CI 0.38 to 0.92) - >18 years aHR 0.59 (95% CI 0.33 to 0.92)   Short IPI (≤18 months)   - all HR 0.86 (95% CI 0.75 to 0.99) - Mexican aHR 0.74 (95% CI 0.49 to 1.07) - Puerto Rican aHR 0.87 (95% CI 0.73 to 1.03) - Other Latina aHR 0.91 (95% CI 0.59 to 1.33) - ≤18 years aHR 0.80 (95% CI 0.65 to 0.96) - >18 years aHR 0.95 (95% CI 0.75 to 1.17) | Home visitation was associated with a small decrease in the risk of a short interpregnancy interval (< 18 months) among Latina women. |

Supplementary table 5. JBI Risk of bias assessment for cross-sectional studies (n=6)

| **First author (year)** | **1. Were the criteria for inclusion in the sample clearly defined?** | **2. Were the study subjects and the setting described in detail?** | **3. Was the exposure measured in a valid and reliable way?** | **4. Were objective, standard criteria used for measurement of the condition?** | **5. Were confounding factors identified?** | **6. Were strategies to deal with confounding factors stated?** | **7. Were the outcomes measured in a valid and reliable way?** | **8. Was appropriate statistical analysis used?** | **Overall appraisal** |
| --- | --- | --- | --- | --- | --- | --- | --- | --- | --- |
| Abdel-Fattah (2007 | Yes | Yes | Yes | Yes | Yes | Yes | Yes | Yes | Low risk |
| Al-Nahedh (1999) | Yes | Yes | Yes | Yes | Yes | Yes | Yes | Yes | Low risk |
| Gunst (2021) | Yes | Yes | Yes | Unclear | No | No | Yes | Yes | Moderate risk |
| Liu (2022) | Yes | Yes | Yes | Yes | Yes | Yes | Yes | Yes | Low risk |
| Olorunsaiye (2023) | Yes | Yes | Yes | Yes | Yes | Yes | No | Yes | Low risk |
| Rozario (2021) | Yes | Yes | Yes | Yes | Yes | Yes | Yes | Yes | Low risk |

Supplementary table 6. JBI Risk of bias assessment for case-control studies (n=2)

| **First author (year)** | **1. Were the groups comparable other than the presence of disease in cases or the absence of disease in controls?** | **2. Were cases and controls matched appropriately?** | **3. Were the same criteria used for identification of cases and controls?** | **4. Was exposure measured in a standard, valid and reliable way?** | **5. Was exposure measured in the same way for cases and controls?** | **6. Were confounding factors identified?** | **7. Were strategies to deal with confounding factors stated?** | **8. Were outcomes assessed in a standard, valid and reliable way for cases and controls?** | **9. Was the exposure period of interest long enough to be meaningful?** | **10. Was appropriate statistical analysis used?** | **Overall appraisal** |
| --- | --- | --- | --- | --- | --- | --- | --- | --- | --- | --- | --- |
| Al-Rumhi (2023) | Yes | Yes | Yes | Yes | Yes | Yes | Yes | Yes | Yes | Yes | Low risk |
| Iacobelli (2014) | Yes | Yes | Yes | Yes | Yes | Unclear | Unclear | Yes | Unclear | Yes | Moderate risk |

Supplementary table 7. JBI Risk of bias assessment for cohort studies (n=44)

| **First author (year)** | **1. Were the two groups similar and recruited from the same population?** | **2. Were the exposures measured similarly to assign people to both exposed an unexposed groups?** | **3. Was the exposure measured in a valid and reliable way?** | **4. Were confounding factors identified?** | **5. Were strategies to deal with confounding factors stated?** | **6. Were the groups/participants free of the outcome at the start of the study (or at the moment of exposure)?** | **7. Were the outcomes measured in a valid and reliable way?** | **8. Was the follow up time reported and sufficient to be long enough for outcomes to occur?** | **Was follow up complete, and if not, were the reasons to loss to follow up described and explored?** | **9. Were strategies to address incomplete follow up utilized?** | **10. Was appropriate statistical analysis used?** | **Overall Appraisal** |
| --- | --- | --- | --- | --- | --- | --- | --- | --- | --- | --- | --- | --- |
| Albrechtson (1998) | Yes | Yes | Yes | Yes | Yes | Yes | Yes | Yes | Yes | Yes | Yes | Low risk |
| Ali (2007) | Yes | Yes | Yes | Yes | Yes | Yes | Yes | Yes | Yes | Yes | Yes | Low risk |
| Arora (2018) | Yes | Yes | Yes | Yes | Yes | Yes | Yes | Yes | Yes | Yes | Yes | Low risk |
| Backley (2020) | Yes | Yes | Yes | Yes | Yes | Yes | Yes | Yes | Yes | Yes | Yes | Low risk |
| Bennett (2006) | Yes | Yes | Yes | Yes | Yes | Yes | Yes | Yes | Yes | Yes | Yes | Low risk |
| Brown (2018) | Yes | Yes | Yes | Yes | Yes | Yes | Yes | Yes | Yes | Yes | Yes | Low risk |
| Brunson (2016) | Yes | Yes | Yes | Yes | Yes | Yes | Yes | Yes | Yes | Yes | Yes | Low risk |
| Caldwell (2022) | Yes | Yes | Yes | Yes | Yes | Yes | Yes | Yes | Yes | Yes | Yes | Low risk |
| Cha (2016) | Yes | Yes | Yes | Yes | Yes | Yes | Yes | Yes | Yes | Yes | Yes | Low risk |
| Cheslack-Postova (2015) | Yes | Yes | Yes | Yes | Yes | Yes | Yes | Yes | Yes | Yes | Yes | Low risk |
| de Bocanegra (2013) | Yes | Yes | Yes | Yes | Yes | Yes | Yes | Yes | Yes | Yes | Yes | Low risk |
| de Bocanegra (2013) | Yes | Yes | Yes | Yes | Yes | Yes | Yes | Yes | Yes | Yes | Yes | Low risk |
| Delara (2018) | Yes | Yes | Yes | Yes | Yes | Yes | Yes | Yes | Yes | Yes | Yes | Low risk |
| El-Kamary (2004) | Yes | Yes | Yes | Yes | Yes | Yes | Yes | Yes | Yes | Yes | Yes | Low risk |
| French (2012) | Yes | Yes | Yes | Yes | Yes | Yes | Yes | Yes | Yes | Yes | Yes | Low risk |
| Gemmill (2013) | Yes | Yes | Yes | Yes | Yes | Yes | Yes | Yes | Yes | Yes | Yes | Low risk |
| Gifford (2021) | Yes | Yes | Yes | Yes | Yes | Yes | Yes | Yes | Yes | Yes | Yes | Low risk |
| Gold (2004) | Yes | Yes | Yes | Yes | Yes | Yes | Yes | Yes | Unclear | Yes | Yes | Low risk |
| Goyal (2017) | Yes | Yes | Yes | Yes | Yes | Yes | Yes | Yes | Yes | Yes | Yes | Low risk |
| Gupta (2019) | Yes | Yes | Yes | Yes | Yes | Yes | Yes | Yes | Yes | Yes | Yes | Low risk |
| Harney (2017) | Yes | Yes | Yes | Yes | Yes | Yes | Yes | Yes | Yes | Yes | Yes | Low risk |
| Heaman (2018) | Yes | Yes | Yes | Yes | Yes | Yes | Yes | Yes | Yes | Yes | Yes | Low risk |
| Holowko (2018) | Yes | Yes | Yes | Yes | Yes | Yes | Yes | Yes | Yes | Yes | Yes | Low risk |
| Isquick (2017) | Yes | Yes | Yes | Yes | Yes | Yes | Yes | Yes | Yes | Yes | Yes | Low risk |
| Kaharuza (2001) | Yes | Yes | Yes | Yes | Yes | Yes | Yes | Yes | Yes | Yes | Yes | Low risk |
| Knutson (2022) | Yes | Yes | Yes | Yes | Yes | Yes | Yes | Yes | Yes | Yes | Yes | Low risk |
| Krans (2018) | Yes | Yes | Yes | Yes | Yes | Yes | Unclear | Unclear | Yes | Yes | Yes | Low risk |
| Lane (2008) | Yes | Yes | Yes | Yes | Yes | Yes | Yes | Yes | Yes | Yes | Yes | Low risk |
| Lewis (2010) | Yes | Yes | Yes | Yes | Yes | Yes | Yes | Yes | Yes | Yes | Yes | Low risk |
| Liberty (2020) | Yes | Yes | Yes | Yes | Yes | Yes | Yes | Yes | Yes | Yes | Yes | Low risk |
| Lindsay (1995) | Yes | Yes | Yes | Unclear | Yes | Yes | Unclear | Unclear | Yes | Yes | Yes | Moderate risk |
| Mishra (2022) | Yes | Yes | Yes | Yes | Yes | Yes | Yes | Yes | Yes | Yes | Yes | Low risk |
| Patel (2021) | Yes | Yes | Yes | Yes | Yes | Yes | Yes | Yes | Yes | Yes | Yes | Low risk |
| Plana-Ripoll (2018) | Yes | Yes | Yes | Yes | Yes | Yes | Yes | Yes | Yes | Yes | Yes | Low risk |
| Raneri (2007) | Yes | Yes | Yes | Yes | Yes | Yes | Yes | Yes | Yes | Yes | Yes | Low risk |
| Reese (2017) | Yes | Yes | Yes | Yes | Yes | Yes | Yes | Yes | Yes | Yes | Yes | Low risk |
| Rubin (2011) | Yes | Yes | Yes | Unclear | Yes | Yes | Yes | Yes | Yes | Yes | Yes | Low risk |
| Smith (2006) | Yes | Yes | Yes | Yes | Yes | Yes | Yes | Yes | Yes | Yes | Yes | Low risk |
| Steenland (2022) | Yes | Yes | Yes | Yes | Yes | Yes | Yes | Yes | Yes | Yes | Yes | Low risk |
| Stevens-Simon (1999) | Yes | Yes | Yes | Yes | Yes | Yes | Yes | Yes | Yes | Yes | Yes | Low risk |
| Waynforth (2015) | Yes | Yes | Yes | Yes | Yes | Yes | Unclear | Unclear | Yes | Yes | Yes | Low risk |
| White (2015) | Yes | Yes | Yes | Yes | Yes | Yes | Yes | Yes | Yes | Yes | Yes | Low risk |
| Woolner (2019) | Yes | Yes | Yes | Yes | Yes | Yes | Yes | Yes | Yes | Yes | Yes | Low risk |
| Yun (2014) | Yes | Yes | Yes | Yes | Yes | Yes | Unclear | Unclear | Yes | Yes | Yes | Low risk |

**Supplementary table 8.** Cochrane risk of bias tool assessment for intervention studies (n=3)

| **First author (year)** | **1. Is it clear in the study what is the cause’ and what is the ‘effect’ (i.e. there is no confusion about it)?** | **2. Were the participants included in any comparisons similar?** | **3. Were the participants included in any comparisons receiving similar treatment/care, other than the exposure or intervention?** | **4. Was there a control group?** | **5. Was there multiple measurement of outcomes both pre and post the intervention/exposure?** | **6. Was follow-up complete and if not, were differences between groups in terms of their follow up adequately described?** | **7. Were the outcomes of the participants included in any comparison measured in the same way?** | **8. Were outcomes measured in a reliable way?** | **9. Was appropriate statistical analysis used?** | **Overall appraisal** |
| --- | --- | --- | --- | --- | --- | --- | --- | --- | --- | --- |
| Gottvall (2002) | Yes | Yes | Yes | Yes | Yes | Unclear | Yes | Yes | Yes | Low risk |
| Kan (2012) | Yes | Yes | Yes | Yes | Yes | Unclear | Yes | Yes | Yes | Low risk |
| Katz (2011) | Yes | Yes | Yes | Yes | Yes | Unclear | Yes | Yes | Yes | Low risk |
